# Global Analysis of Lysine Acetylation Suggests the Involvement of Protein Acetylation in Diverse Biological Processes in Rice (*Oryza sativa*)

**DOI:** 10.1371/journal.pone.0089283

**Published:** 2014-02-20

**Authors:** Babi Ramesh Reddy Nallamilli, Mariola J. Edelmann, Xiaoxian Zhong, Feng Tan, Hana Mujahid, Jian Zhang, Bindu Nanduri, Zhaohua Peng

**Affiliations:** 1 Department of Biochemistry and Molecular Biology, Mississippi State University, Starkville, Mississippi, United States of America; 2 Institute of Genomics, Biocomputing and Biotechnology, Mississippi Agricultural and Forestry Experimental Station, Mississippi State University, Starkville, Mississippi, United States of America; 3 College of Veterinary Medicine, Mississippi State University, Starkville, Mississippi, United States of America; Instituto de Biotecnología, Universidad Nacional Autónoma de México, Mexico

## Abstract

Lysine acetylation is a reversible, dynamic protein modification regulated by lysine acetyltransferases and deacetylases. Recent advances in high-throughput proteomics have greatly contributed to the success of global analysis of lysine acetylation. A large number of proteins of diverse biological functions have been shown to be acetylated in several reports in human cells, *E.coli*, and dicot plants. However, the extent of lysine acetylation in non-histone proteins remains largely unknown in monocots, particularly in the cereal crops. Here we report the mass spectrometric examination of lysine acetylation in rice (*Oryza sativa*). We identified 60 lysine acetylated sites on 44 proteins of diverse biological functions. Immunoblot studies further validated the presence of a large number of acetylated non-histone proteins. Examination of the amino acid composition revealed substantial amino acid bias around the acetylation sites and the amino acid preference is conserved among different organisms. Gene ontology analysis demonstrates that lysine acetylation occurs in diverse cytoplasmic, chloroplast and mitochondrial proteins in addition to the histone modifications. Our results suggest that lysine acetylation might constitute a regulatory mechanism for many proteins, including both histones and non-histone proteins of diverse biological functions.

## Introduction

Protein acetylation is a highly conserved posttranslational modification (PTM) in prokaryotes and eukaryotes, that is probably more conserved than protein phosphorylation [1], although it is considered to be less common than phosphorylation or ubiquitination [1], [2]. Acetylation is a covalent modification, where an acetyl group is transferred from acetyl-coenzyme A by acetyltransferases, and it might either affect the α-amino group of a protein N-terminus, or the ε-amino group of a lysine residue. The N-terminal acetylation, catalyzed by N-terminal acetyltransferases (NATs) is an irreversible modification, which occurs during the protein synthesis. Conversely, lysine acetylation at the ε-amino group is a reversible, dynamic modification regulated by lysine acetyltransferases (KATs) and lysine deacetylases (KDACs), and is much less common than widespread N-terminal acetylation. It has been originally identified as a post-translational modification of histones by Allfrey *et al*., 1964 [3] and since then it has been found to be highly conserved in both prokaryotes and eukaryotes [4].

Lysine acetylation in histones plays a central role in epigenetic control of gene expression by regulation of the chromatin structure. Acetylation neutralizes the positive charges of lysine residues and decreases histone affinity to negatively-charged DNA molecules thereby increasing the accessibility of DNA to transcription factors [5], [6]. Furthermore, transcriptional co-regulators and chromatin remodeling factors can also recognize the acetylated lysine residues. Histones can be modified by acetylation on multiple residues, and the most important acetylation targets include K9, K14, K18, K23, and K27 of histone H3, K5, K8, K12, K16, and K20 of histone H4, K5, K9, and K13 of H2A, as well as K5, K12, K15, and K20 of H2B [7], [8]. Histone acetylation on lysine residues results in relaxed state of chromatin structure, which is often associated with increased gene activity, whereas deacetylation leads to a compact state of chromatin structure and thus transcriptional repression. Acetyltransferases, which catalyze the lysine acetylation, are classified as type A or B, depending on their subcellular distribution. Type A acetyltransferases are involved in the acetylation of nuclear histones, whereas type B acetyltransferases are cytoplasmic proteins involved in acetylation of histones in cytoplasm [9].

Recent advances in high-throughput mass spectrometry-based proteomics have made substantial contributions to the global analysis of lysine acetylation and a large number of acetylated non-histone proteins of diverse biological function have been identified. For example, 195 acetylated proteins on 388 sites have been identified in HeLa cells and mouse liver mitochondria with the help of acetyllysine antibody and high-throughput mass spectrometry [10]. Similar methodology used in combination with high-resolution mass spectrometric analysis yielded identification of 3600 lysine acetylation sites on 1750 proteins in human cells [1]. Interestingly, a large number of acetylation sites are also shown in prokaryotes, such as *Escherichia coli* and *Salmonella* [11], [12]. For instance 138 acetylation sites on 91 proteins have been identified in the global analysis of *Escherichia coli’*s acetylome [11].

Plant proteins can also be acetylated, highlighted by the fact that multiple acetyltransferases and deacetylases regulating this modification have been identified in the genome of various plant species. The *Arabidopsis* genome contains at least twelve histone acetyltransferases (HAT) and eighteen histone deacetylases (HDAC) [13]. The rice genome contains 19 HDAC genes [14] and using the chromatin database, seven HAT genes were identified in rice genome (http://www.chromdb.org/). It has been shown that histone acetylation plays a significant role in the regulation of cell cycle, development, flowering time, and hormone signal transduction in plants [8]. Dynamic and reversible changes in histone H3 acetylation is observed at two submergence-inducible genes, alcohol dehydrogenase 1 (ADH1) and pyruvate decarboxylase 1 (PDC1) in rice [15]. It has been shown that H3K9 and H4K12 acetylation status is elevated in euchromatic regions in rice [16]. Increased H3K9 acetylation at the *RFT1* locus (RICE FLOWERING LOCUS T 1) is correlated with the activation of *RFT1* transcription, which encodes a mobile flowering signal and promotes floral transition under short-day conditions in rice [17], [18]. Tan *et al*., 2011 [19] carried out large-scale analysis of histone modifications in response to cell wall removal and regeneration in rice. They found that differential H3K18 and H3K23 acetylation is closely associated with cell wall removal. Acetylation on H3K18 and H3K23 were identified and quantified using isotope labeling assisted mass spectrometry-based approach.

Despite the extensive studies on lysine acetylation in histones, the extent of lysine acetylation in non-histone proteins in plants has remained largely unknown until recently. In 2011, two large-scale analyses of lysine acetylation in *Arabidopsis* were reported. One study identified 91 acetylated sites on 74 proteins of diverse functional classes, and another one identified 64 acetylated sites on 57 proteins [20], [21]. These results indicated that lysine acetylation is important in the regulation of key metabolic enzymes in *Arabidopsis*. The identified acetylated proteins included photosynthesis-related proteins, such as Photo system II (PSII) subunits, light-harvesting chlorophyll a/b-binding proteins, RuBisCO large and small subunits, and chloroplastic ATP synthase (β-subunit) [21]. Interestingly in 2012, one more study related to lysine acetylation in Grapevine (*Vitis vinifera*) was reported with the identification of 138 lysine acetylated sites [22]. Rice serves as the staple food for over half of the world’s population and it is a model plant for plant biological studies of monocots, particularly the cereal crops. The presence of a large number of acetyltranferases and deacetylases in the rice genome suggests that acetylation of non-histone proteins may also play an essential role in rice development and metabolism. Here we report a proteomics study of lysine acetylation in rice. We identified 60 lysine acetylated sites on 44 proteins controlling diverse biological functions in various cellular components. Gene ontology analysis clearly demonstrated that lysine acetylation in rice is not limited to histones, but occurs in diverse proteins localized in compartments such as cytoplasm, nucleus, chloroplasts and mitochondria.

## Materials and Methods

### Plant Materials and Growth Conditions

The growth conditions of rice (*Oryza sativa*, cultivar Nipponbare) were the same as in our previous study [23], [24]. Plants were grown in the greenhouse of the Department of Biochemistry and Molecular Biology, Mississippi State University, MS, USA.

### Suspension Cell Culture

Rice (*Oryza sativa*) NB2P suspension cell cultures were maintained as reported [25], [26]. Briefly, suspension cells were grown at 24°C with constant shaking on a gyratory shaker at 150 rpm in B5 liquid medium (pH 5.7) containing 20 g/L sucrose, 0.5 g/L MES, 2.0 mg/L 2,4-dichlorophenoxyacetic acid (2,4-D), 2 g/L casein enzymatic hydrolysate, and 0.005% (w/v) pectinase. Suspension cells were subcultured weekly. Healthy looking suspension cells were harvested three days after subculture for protein extraction.

### Protein Extraction and Tryptic Digestion

Proteins were isolated using phenol extraction method [27], [28] from three biological replicate samples. Ten grams of exponentially growing cell suspension culture was harvested three days after subculture and ground in liquid nitrogen. Protein extracts were prepared in an extraction buffer (0.9 M sucrose, 0.5 M Tris-HCl pH 8.7, 0.05 M EDTA, 0.1 M KCl, and 2% β-mercaptoethanol added freshly), mixed with an equal volume of saturated phenol (pH 8.0) and then homogenized for 10 minutes. The homogenate was centrifuged at 2500×*g* for 10 minutes, the phenol phase was recovered and the phenol extraction was repeated three times. The final collection of phenol was mixed with five volumes of precipitation buffer (methanol with 0.1 M ammonium acetate and 1% β -mercaptoethanol). Precipitation was carried out at −70°C overnight. The precipitant was recovered by centrifugation at 13400×*g* for 10 minutes and the pellet was washed three times with cold precipitation buffer and then three times with ice cold 70% ethanol. The protein pellet was lyophilized to powder in a speed vacuum (LABCONCO, model LYPH-LOCK 6) and stored at −70°C for further analysis. The triplicates protein samples were digested with trypsin at pH 7.8 using a trypsin:substrate ratio of 1∶40, followed by peptide purification by C18 Sep-Pak columns (#WAT020515, Waters, USA) exactly as reported [29].

### Immunoaffinity Purification of Lysine-acetylated Peptides

Triplicate of samples were used for affinity purification of the lysine-acetylated peptides. The volume of peptide samples was concentrated using vacuum centrifugation and dissolved in 400 µl MOPS immunoprecipitation buffer (50 mM MOPS pH 7.2, 10 mM NaH_2_PO_4_, 50 mM NaCl). Peptides were incubated with 30 µl anti-acetyl-lysine antibody conjugated to agarose (ICP0388, Immunechem, USA) for 12 hours at 4°C as it has been previously published [30]. In the next step, agarose beads were washed four times at 4°C with the MOPS immunoprecipitation buffer and once with deionized water. The acetylated peptides were eluted from the resin using 50 µl 0.1% formic acid, which was incubated with the beads for 15 minutes at room temperature with gentle shaking. The elution step was repeated twice. The samples were further purified by reversed-phase C18 columns, and after the volume was concentrated on vacuum centrifuge, the sample volume was adjusted to 40 µl in solvent containing 2% acetonitrile and 0.1% formic acid.

### Protein Identification by Mass Spectrometry

Peptides were separated by using 75-µm i.d. ×15 cm reversed-phase column (fused-silica C18 column, Thermo) controlled by an Ultimate 3000 nanoflow HPLC (Dionex). Peptides were eluted using a 180-minute gradient (2%–95% solvent B containing 95% acetonitrile and 0.1% formic acid) at a flow rate of 0.5 µl/min, and introduced into an Orbitrap Velos mass spectrometer (Thermo Fisher). The Orbitrap was operated in the data-dependent mode, where the full scan MS spectra (300–2,000 amu) were acquired with a resolution of 100,000 and analyzed by an FT-MS analyzer. The five most intense ions were then selected for collision-induced (CID) fragmentation in the OrbiTrap at normalized collision energy of 35% and activation time of 40 ms.

The acquired data were analyzed by Proteome Discoverer 1.3 (Thermo) using Sequest, with minimum precursor mass of 350 Da and maximum 5000 Da. The signal to noise threshold used for FT was 1.5. The searches were done against the NCBI reference genome database for *Oryza sativa* (August 2011) using only full tryptic peptides with maximum missed cleavage site of four, precursor mass tolerance of 10 ppm and fragment mass tolerance of 0.8 Da. The protein modifications allowed in the search included N-terminal acetylation (+42.011 Da), carbamidomethyl on cysteine (+57.021 Da), oxidation on methionine (+15.995 Da), acetylation on lysine (+42.011 Da), and trimethylation on lysine (+42.047 Da). The maximum number of peptides considered was 500 and the fragment ion cutoff percentage was 0.1. The peptides were grouped by mass and sequence, and the proteins were also subjected to protein grouping, taking into account only the PSMs with confidence at least medium and delta Cn>0.15. The Peptide Validator option was used, where the reverse decoy database search was performed. The following cut-off values were used: the results were filtered similarly as reported Kelly *et al*., 2009 [31], using the maximum Cn value of 0.1, the normalized XCorr values of 1.5, 2, 2.5, 3 and 4 for charge states of 1+, 2+, 3+, 4+, 5, and the peptide rank of 1 [32].

### Gene Ontology Analysis

Functional classification of proteins was carried out according to the gene ontology (GO) rules using the AgBase at http://www.agbase.msstate.edu/ [33]. Three independent gene ontologies were used to describe the function of gene products such as cellular component (CC), molecular function (MF) and biological process (BP). GO annotations were obtained from GORetriever, a tool available at AgBase [33]. GOSlimViewer tool was used to obtain the summary data to generate GO pie charts (AgBase). Proteins without annotations at AgBase were searched in other databases, including NCBI, UniProt, Gramene and TIGR (Rice Genome Annotation Resource) [34].

### Western Blot Analysis

Protein samples were isolated from four different rice tissues such as suspension cells, endosperm (5 days after anthesis), flower and leaf. Proteins samples were separated on SDS-polyacrylamide gel and transferred to PVDF (Millipore) membrane. Acetylated Lys antibody (ImmuneChem) was used in a 1∶1000 dilution as reported [20].

### Protein Secondary Structure Prediction

Protein structural analysis was carried out according to the secondary structure prediction rules using PROTEUS Structure Prediction Server 2.0 at http://www.proteus2.ca/proteus2/index.jsp [35].

## Results

### Detection of Lysine Acetylation in Rice

**Figure 1:**
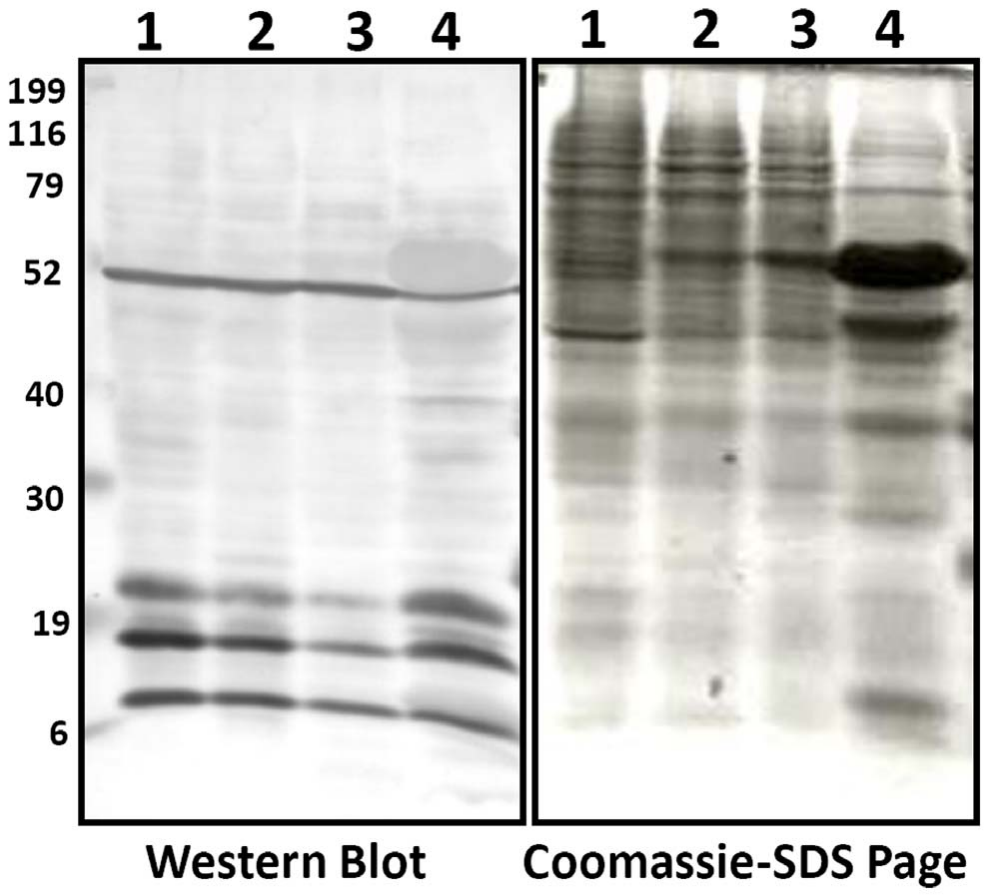
Lysine acetylation status is analyzed by using SDS-PAGE and Western blotting. Protein samples were collected from different tissues: 1. Suspension cells, 2. Endosperm (5 days after anthesis), 3. Flower, and 4. Leaf. Equal amount of protein samples were loaded for western blot analysis. Proteins samples were separated on SDS-polyacrylamide gel and transferred to PVDF (Millipore) membrane. Acetylated Lys antibody (ImmuneChem) was used in a 1∶1000 dilution as previously described Finkemeier *et al*., 2011 [20].

Antibodies specifically recognizing acetylated lysine have been successfully used to detect the lysine acetylated proteins in different organisms such as *Arabidopsis*, human cells and *E. coli* [1], [10], [12], [20], [21]. To detect the status of lysine acetylation in rice proteome, proteins from four different tissues, including suspension cells, endosperm, flower and leaf, were examined by Western blotting using antibodies specific for acetylated lysine. Multiple major protein bands with molecular weight higher than histones were detected (**Fig. 1**), indicating that lysine acetylation is probably not limited to histones but also occurs in other rice proteins. Most clearly, four distinct protein bands were co-migrated with 52, 25, 19, and 6 kD standard protein markers (**Fig. 1**). In addition, multiple weaker bands plus smears were observed, which were different from the patterns revealed by Coomassie blue stain.

### Global Mapping of Acetylated Peptides by Immunoaffinity Purification Coupled with LC-MS/MS

**Figure 2:**
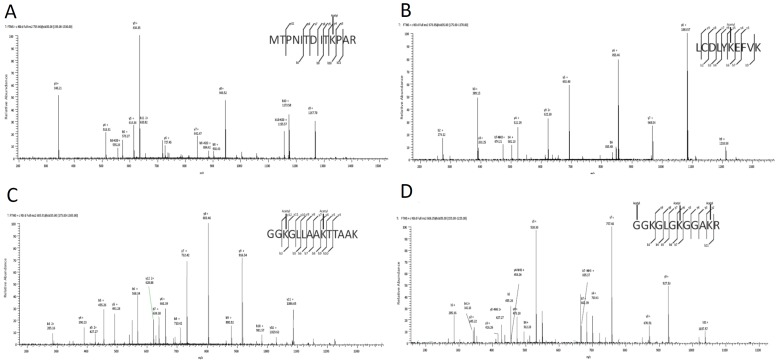
Representative fragmentation spectra of acetylated peptides in rice. The detected b- and y- fragment ion series are shown. The CID fragmentation spectrum of acetylated peptide unique to (A) Dihydroorotate dihydrogenase, (B) Enolase, (C) Histone H2A, and (D) Histone H4.

To map lysine acetylation on a global scale, we used immunoaffinity enrichment combined with high-resolution mass spectrometry. Rice cell suspension cultures were selected as experimental materials because they provide good amount of protein extracts of actively dividing cells and are easy to maintain and collect. Proteins extracted from exponentially growing cells were digested with trypsin and the generated peptides were subjected to affinity enrichment with antibodies specific for acetyllysine. Antibody based immunoaffinity purifications were highly recommended for the enrichment of specific modified peptides because antibodies can easily distinguish proteins with different modifications such as acetylation, methylation and trimethylation. The enriched peptides were analyzed by a high-resolution Orbitrap mass spectrometer operated in the fourier transform (FT) mode. High-resolution Orbitrap mass spectrometer was successfully used to distinguish different protein modifications like acetylation and trimethylation in different organisms. The mass difference of the two modifications is 0.03 Da. Our mass spectrometer was operated at the resolving power of 100,000 on the orbitrap, which was sufficient to distinguish between these two modifications [36]. The criteria we used for identifying acetylated peptide included an accurate measurement of precursor and product ions and a mass difference of m/z 170 between two “y” or “b” ions to establish an acetylated lysine. In our previous studies [19], we included another criterion, a unique immonium ion at m/z 126.1 of acetylated lysine, which is a further fragment ion induced by the loss of NH_3_ from the acetylated lysine immonium ions at *m*/*z* 143.1, typical of an acetylated lysine residue. Due to the substantial improvement of mass spectrometory resolution, we did not observe inconsistencies between accurate ion measurements and immonium ion detection in our preliminary studies. More importantly, the samples we used for mass analysis were enriched with antibodies specific for acetylated lysine. Although the acetyl group and the trimethyl group are very close in mass, they are highly different in structure and property. The antibodies can easily distinguish these two groups. The anti-acetyl-lysine antibody (ImmuneChem) we used is highly specific for the acetyl group and has been successfully used to global identification of lysine acetylated proteins in different organisms including *Arabidopsis,* human cells and *E. coli* [1], [10], [12], [20], [21]. In the acetyl group affinity enriched sample, the trimethylated peptides are largely eliminated. The chance for the acetylation modification to be confused by trimethylation is small, if not impossible. In addition, we noted that immonium ions were not used as a criterion in the identification of acetylated lysine in multiple publications using advanced mass spectrometry analysis [1], [21], [36]. Therefore, we did not include immonium ion data here. Using this approach followed by data analysis and manual examination of the mass spectra, we identified 74 lysine acetylated sites from 52 different proteins of diverse biological functions. Among them, 8 peptides had an acetylation at the C-terminals and 6 peptides might have acetylaton at both interior and C-terminal sites. All the other peptides have interior acetylated lysine. C-terminal lysine acetylation has been observed in other acetylome studies, where trypsin was used for protein digestion [37], [38]. The proposed explanation is that the C-terminal lysine could be digested by cellular proteinases, and in this case these lysines could be in the C-terminus prior to the tryptic digestion [37]. Alternatively, pre-existing C-terminal acetylation may have critical biological functions in many proteins [38]. Plant cells have a big vacuole which occupies up to 90% of the cell volume. The vacuole contains a large amount of various proteinases which are released during sample grinding. The detection of C-terminal lysine acetylation is highly possible. Given that trypsin does not cleave after modified acetyl lysine, however, the C-terminal lysine acetylation sites were not included in our further analyses. Apart from the C-terminal acetylated lysine sites, we had identified 60 lysine acetylated sites from 44 different proteins with high confidence (**Table 1**, **Table S1** and **Figure S1**). Some examples of the mass spectra are shown in **Fig. 2** and all the other spectra are shown in **Figure 1**. Many non-histone proteins, which had not been previously shown to be modified by lysine acetylation, were found to be acetylated.

**Table 1:** List of lysine acetylated proteins identified in rice.

Accession^a^	Protein Description^b^	Peptide^c^	Position^d^
A2YDW3	Hypothetical protein	NNKTMAVC**K(ac)**NAKGTATGCLK	k349
Q7XLN2	Transposon protein, putative, CACTA,En/Spm subclass	VNCEMIAKYPQATEDNLVHLLKEQHFKTPAESNVYDLMD**K(ac)**K	k722
Q7XHY6	Proline-rich family protein expressed	ELAM**K(ac)**FEKGLNTATLLSNEVK	k82
Q7XUC9	Histone H4, putative, expressed	GGKGLG**K(ac)**GGA**K(ac)**R	k13, k17
Q6AUA8	Hypothetical protein	CTTP**K(ac)**TLKWDEITLPEK	k102
Q6ASW7	E3 SUMO-protein ligase SIZ2	QGR**K(ac)**QELVDK	k41
Q84T08	BHLH transcription factor, putative	RF**K(ac)**AS**K(ac)**SSGDNSSLR	k93, k96
Q0J8A8	Lectin-like receptor kinase 1, putative	LP**K(ac)**NASSSGLGLTN**K(ac)**SYTNVSTGEDR	k128, k140
Q109E6	Hypothetical protein	RI**K(ac)**PESKPLKELK	k62
Q8L4Q4	cytochrome P450 72A1, putative, expressed	DLTNPYFAHLLGKGLVLIDGDEW**K(ac)**RHYKVVHPAFDMDK	k151
Q8LNC1	Transposon protein, putative, Mutator sub-class	A**K(ac)**VIAEPTATD**K(ac)**GK	k729, k739
Q0JD76	Os04g0423600 SET domain containing protein	A**K(ac)**DLLECINHVQ	k631
Q2R286	Retrotransposon protein, Ty3-gypsy subclass	ISPTRDVYCPIQ**K(ac)**TKNHDLSSCKVFLSAMK	k157
Q0D9B8	Os06g0728200 Hypothetical protein	EGNMEEFLEEV**K(ac)**ERL**K(ac)**KELK	K381, k385
C7J3B8	Os06g0515301 protein	FAGGSRDTCAKLSGC**K(ac)**IVDGNC**K(ac)**PPYVHHTLHPEAGK	k60, k67
Q6F2N5	ZOS5-08 - C2H2 zinc finger protein, expressed	KHGAKPFACRRCAKPFAV**K(ac)**GDWR	k310
Q339E8	Retrotransposon protein, Ty1-copia subclass	LCHVNFGCMTSLANMSLIPKFTLVKGS**K(ac)**CHTCVQSK	k495
Q0JMV9	AIG1 family protein, expressed	ESDDMKLC**K(ac)**EDCISDCFAMEEDDMIK	k285
Q84R47	Putative gypsy-type retrotransposon protein	MQAA**K(ac)**ISQLE**K(ac)**QIR	k556, k562
Q6Z744	Dihydroorotate dihydrogenase protein	MTPNITDIT**K(ac)**PAR	k227
Q8GSZ9	Armadillo repeat-containing protein	ILMATAIS**K(ac)**MFLSEPMKSSLGEDGAVEPLVEMF**K(ac)**SGNLEAK	k202, k227
Q7FAH2	Glyceraldehyde-3-phosphate dehydrogenase	TVDGPSS**K(ac)**DWR	k196
Q5Z579	Hypothetical protein [Oryza sativa Japonica Group]	CKLGSLG**K(ac)**PNEPSR	k220
Q6AV27	Hypothetical protein	QTGQQ**K(ac)**GGASRKAR	k51
Q7GBK0	Histone H2B.7	LPAG**K(ac)**GE**K(ac)**GSGEGK	k43, k46
Q94JJ4	Core histone H2A/H2B/H3/H4 domain containing	LPAG**K(ac)**AE**K(ac)**GSGEGK	k43, k46
Q0JQP0	Core histone H2A/H2B/H3/H4 domain containing	KPAA**K(ac)**KPAEEEPAAEKAE**K(ac)**APAG**K(ac)**KPK	k12, k26, k31
B8AV15	Hypothetical protein	AGFL**K(ac)**HNLWVTSYK	k599
Q6Z0V3	Aminotransferase-like protein	TPPE**K(ac)**SWITWYK	k108
B8AJU1	Histone H2A	GG**K(ac)**GLLAA**K(ac)**TTAAK	k7, k13
Q6F362	Core histone H2A/H2B/H3/H4 domain containing	AE**K(ac)**KPAA**K(ac)**KPAEEEPAAE**K(ac)**APAAGKKPK	k7, k12, k23
A2ZVA8	Acetyltransferase, GNAT family, putative, expressed	YY**K(ac)**NITPPDCYVLTK	k158
Q6L4P8	Hypothetical protein [Oryza sativa Japonica Group]	TI**K(ac)**ILLR	k7
Q7XDV6	Hypothetical protein	E**K(ac)**NFWARHSTSCSPMPGK	k40
Q7XXQ5	Basic helix-loop-helix DND-binding domain containing	LNERFLELGAVLEPG**K(ac)**TPKMDK	k123
Q10QA8	Somatic embryogenesis related protein, putative	HDLYEQTNRSPTP**K(ac)**TEEEQIAK	k348
B9EY36	Hypothetical protein OsJ_02631	ELEEVVEYL**K(ac)**NPSK	k348
Q2QT84	Transposable element protein, putative, MuDR	**K(ac)**HAVQECVLKVDGGCSCTCM**K(ac)**PK	k523, k543
Q6K674	Translation initiation factor IF-3-like	**K(ac)**QAIELLR	k112
Q6H708	Hypothetical protein	**K(ac)**YFILFK	k59
Q2R0G5	Retrotransposon protein, putative, unclassified	TVDGVLLKCLGPEEA**K(ac)**TVMSEVHEGICGTHQSAHKMK	k1731
Q2QLX6	GATA zinc finger family protein, expressed	RCTHCLSY**K(ac)**TPQWR	k246
Q2QRJ1	Transposon protein, putative, CACTA,En/Spm subclass	QHTCIPYYKF**K(ac)**GGEQTRTREK	k529
B9G3A0	Enolase, putative, expressed	LCDLY**K(ac)**EFVK	k313

a Uniprot Accession number,

b Protein name (Proteins without annotations at NCBI were searched in other databases such as UniProt, TIGR (Rice Genome Annotation Resource) and Gramene),

c Peptide sequence (Acetylated lysine is marked with “ac”),

d Site of acetylated lysine in protein.

### The Acetylation Sites Display Substantial Sequence Bias

**Figure 3:**
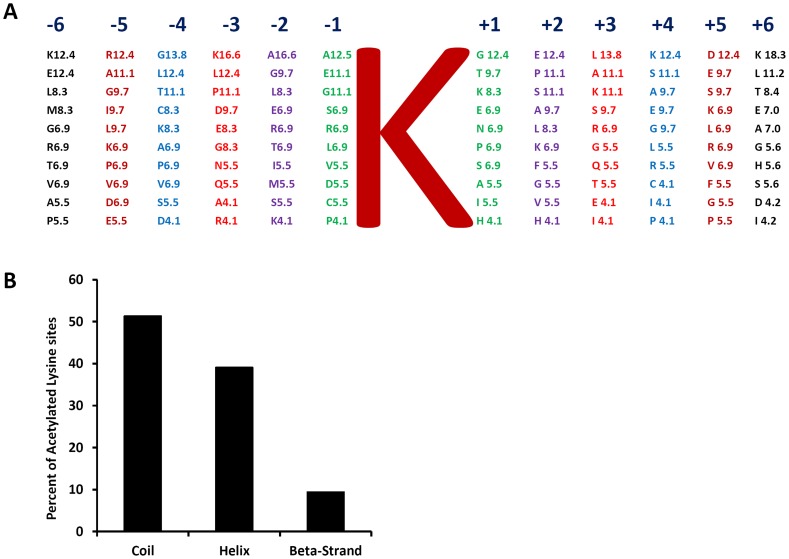
Characteristics of acetylated lysine sites in rice. (A) Amino acid frequency percentages for ±6 amino acids from lysine acetylated site. Letter K in the center represents the acetylated lysine. Amino acid frequency percentages are calculated by considering all identified lysine acetylated sites on different proteins in rice. (B) Different secondary structural regions of acetylated lysines identified in rice.

To understand the regulation of protein acetylation and identify a possible consensus motif, we examined the occupancy frequency of amino acids in positions surrounding the identified acetylated lysine residues. Substantial bias in amino acid distribution was observed from position −20 to +20 around the modified lysine although a well-defined consensus sequence was not detected. For example, charged amino acid K and nonpolar amino acids A, G and L presented in much higher frequency than the average of the 20 amino acids in these positions (**Table S2**). In contrast, charged amino acid H and the aromatic amino acids (W, Y, and F) were presented in much lower frequency. The amino acids in positions −2 to + 2 around the lysine acetylation sites were examined in *Arabidopsis* [20]. We found that rice and *Arabidopsis* shared high sequence similarity in these positions. For example, the preferred top five amino acids in −2 position were A, G, L, E, and R in rice (**Fig. 3A**) and G, L, A, E, and T in *Arabidopsis* in the presented order. The preferred top five amino acids in the −1 position were A, E, G, S, and R in rice (**Fig. 3A**) and G, A, D, S, and E in *Arabidopsis*. The amino acids from −6 to + 6 were examined in human cells [1]. Plants and human cells share little sequence similarity in positions from −3 to +2 around the acetylation sites. Interestingly, the amino acids share moderate degree of similarity in positions −6 to −4 and +3 to +6. For example, the charged amino acids K, R, and E presented in high frequency in these positions in both rice and human cells (**Table S2** and **Fig. 3A**). Secondary structure analysis showed that the acetylation sites distribution was about 52% in coil, 39% in helix, and 9% in beta-strand (**Fig. 3B** and **Table S3**), which was highly similar to the distribution in human cells [1].

### Gene Ontology Analysis of the Acetylated Proteins

**Figure 4:**
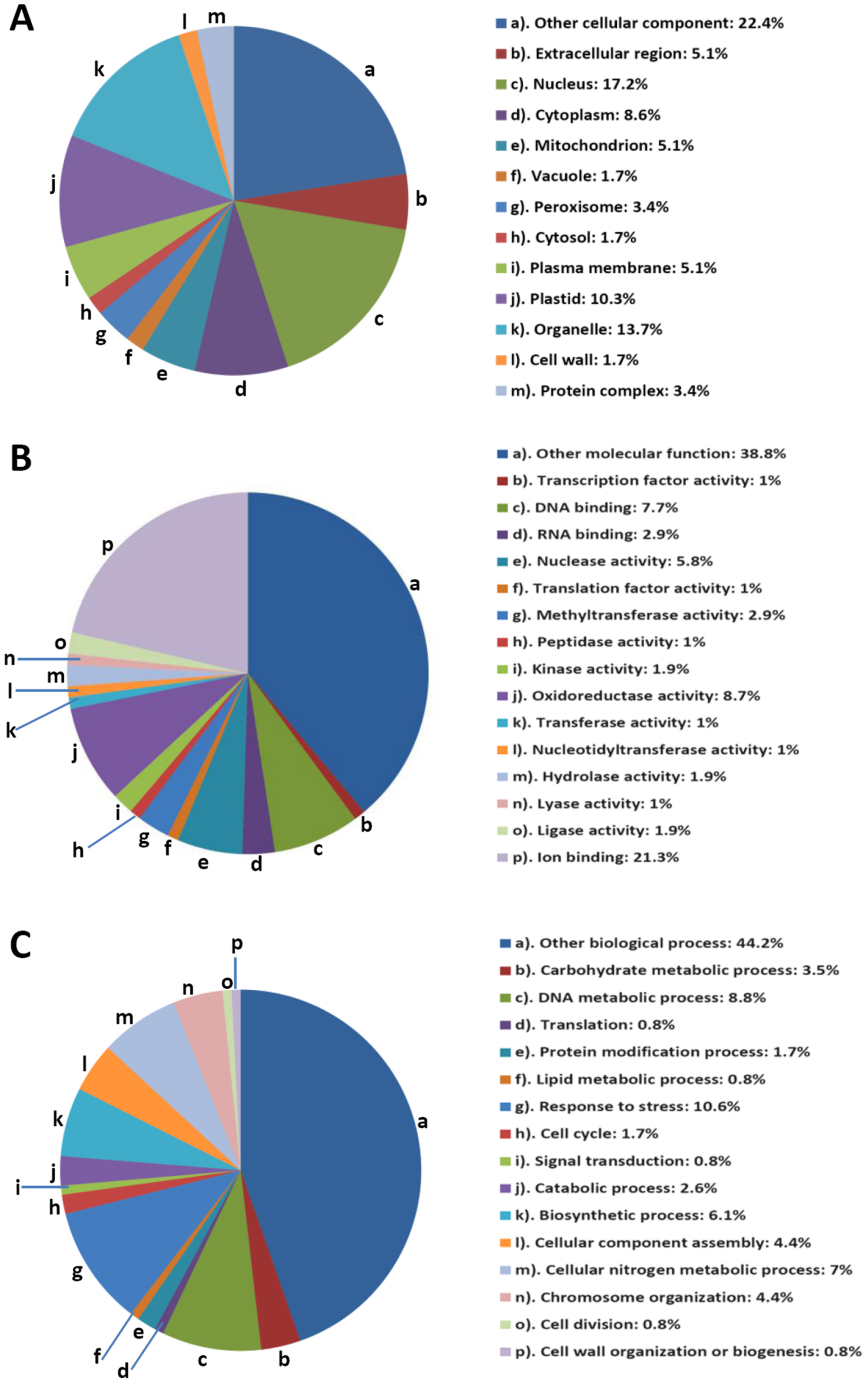
Distribution of the identified lysine acetylated proteins among different Gene Ontology categories. The pie chart was generated using the analysis results of the GOSlimViewer tool at AgBase. Percentage distribution of the unique proteins was used to make the pie charts. Gene distribution was grouped on the basis of Cellular Components (A), Molecular Functions (B) and Biological Processes (C).

**Figure 5:**
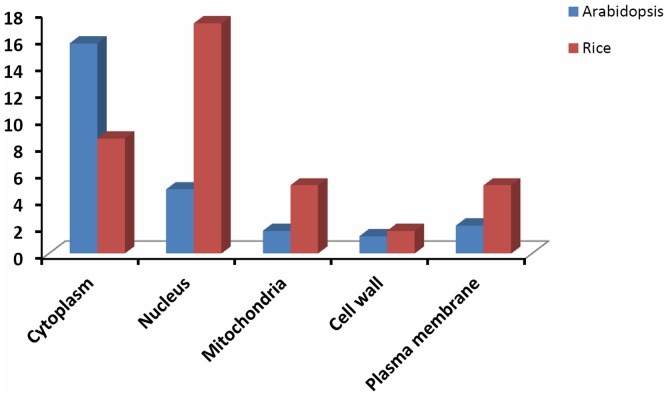
Comparison or subcellular distribution of lysine acetylated proteins in *Arabidopsis* and rice. Cellular components of identified rice acetylated proteins were compared with the recently reported *Arabidopsis* acetylated proteins data [21].

For a comprehensive analysis of the distribution and function of the acetylated proteins, we carried out GO analysis using the AgBase at http://www.agbase.msstate.edu/ [33]. GO annotations of identified proteins were obtained from different databases such as AgBase, NCBI, UniProt, Gramene and TIGR (Rice Genome Annotation Resource) [34]. Three independent categories of gene ontologies were used to describe the function of gene products, which were cellular component, molecular function and biological process in which the gene product participates. Analysis of protein distribution within the cellular component (**Fig. 4A** ) indicated that the majority of identified lysine acetylated proteins were localized in nucleus (17.2%), followed by organelle (13.7%), plastid (10.3%), cytoplasm (8.6%), mitochondria (5.1%), plasma membrane (5.1%), peroxisome (3.4%), vacuole (1.7%), cell wall (1.7%), etc. Total nuclear proteins occupy a substantial fraction of the acetylated proteins in rice. Compared with the subcellular distribution profile of *Arabidopsis* acetylated proteins, we identified more proteins in nucleus, mitochondria, and plasma membrane. In contrast, the percentage of proteins in cytoplasm was smaller (**Fig. 5**).

Molecular function analysis showed that proteins with ion binding (21.3%), oxidoreductase activity (8.7%), DNA binding (7.7%), nuclease activity (5.8%), RNA binding (2.9%), methyltransferase activity (2.9%), kinase activity (1.9%), and hydrolase activity(1.9%) account for over half of all the identified proteins (**Fig. 4B**). Other molecular functions included: ligase activity (1.9%), lyase activity (1%), nucleotidyltransferase activity (1%), peptidase activity (1%), transcription factor activity (1%), translation factor activity (1%) and transferase activity (1%). Biological process analysis showed that response to stress (10.6%), DNA metabolic processes (8.8%), cellular nitrogen metabolic process (7%), biosynthetic process (6.1%), chromosome organization (4.4%), cellular component assembly (4.4%), carbohydrate metabolic process (3.5%) and catabolic process (2.6%) were the major biological processes in which the acetylated proteins are involved (**Fig. 4C**).

### Selected Examples of Acetylated Proteins

#### 1) Lysine acetylation of nuclear proteins

Histones are well known for their modification by lysine acetylation and methylation. In the present study, we identified six histone proteins with 14 lysine acetylation sites (**Table 1**). K13 and K17 of histone H4; K43 and K46 of histone H2B.7; K7 and K13 of histone H2A are found to be lysine acetylated. In addition to these core histones, we found that three core histone H2A/H2B/H3/H4-domain containing proteins (Q94JJ4 (Histone H2B.4), Q6F362 (Histone H2B.9) and Q0JQP0 (Os01g0149400 protein)) were also acetylated. Transcription factors such as BHLH transcription factor, GATA zinc finger family protein, and basic helix-loop-helix DNA-binding protein are also acetylated. Transposon proteins and retrotransposon proteins were the biggest group of acetylated nuclear proteins with 9 members being acetylated (**Table 1**). Two lysine acetylated sites were identified in two transposon proteins which belonged to CACTA, En/Spm sub-class (Q7XLN2 and Q2QRJ1). Four other lysine acetylated sites were identified in two mutator sub-class transposon proteins. We also identified five retrotransposon proteins with acetylated lysine sites in six positions. Out of the five acetylated retrotransposon proteins, one protein belong to Ty3-gypsy subclass, one protein belongs to Ty1-copia subclass and three proteins belong to an unclassified subclass (**Table 1**).

#### 2) Lysine acetylation of protein kinases

**Figure 6:**
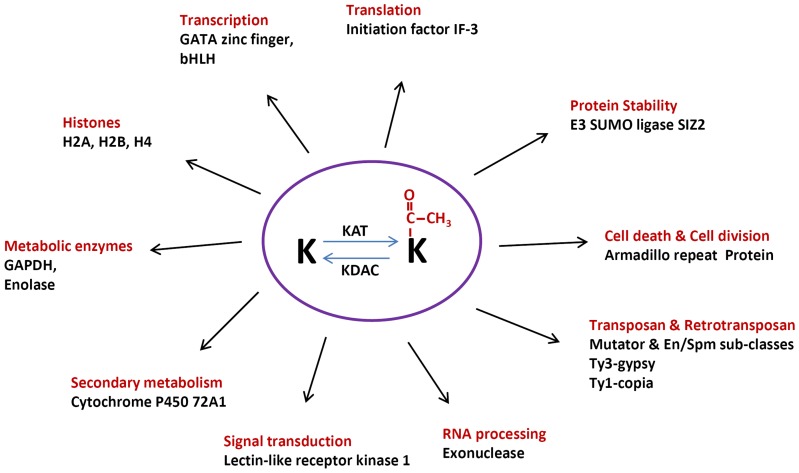
Diversified functions of lysine acetylation in various cellular processes in rice. K represent lysine residue, KAT represents lysine acetyltransferase, KDAC represents lysine deacetylase.

We identified one rice protein kinase to be lysine acetylated and its function is presently unknown. Two lysine acetylation sites (K128 and K140) were identified as acetylation sites in lectin-like receptor kinase 1 (**Table 1** and **Fig. 6**). In plants, lectin-like receptor kinases have diverse role in plant microorganism interaction and oligosaccharide signal transduction.

#### 3) Lysine acetylation of metabolic proteins

Several important metabolic proteins (**Table 1** and **Fig. 6**) were found to be lysine acetylated including glyceraldehyde-3-phosphate dehydrogenase (GAPDH, Q7FAH2), enolase (B9G3A0), cytochrome P450 72A1 (Q8L4Q4), and dihydroorotate dehydrogenase (Q6Z744). Previous studies already indicated that nuclear translocation of GAPDH is mediated by acetylation of three lysine residues at position117, 227 and 251 in human cells [39]. Lysine acetylome studies have indicated that protein acetylation plays a critical role in the regulation of metabolism in *Arabidopsis*, *Escherichia coli* and human cells [1], [11], [20], and our results are consistent with these preceding reports.

#### 4) Lysine acetylation of acetyltransferase

Acetyltransferases are responsible for addition of an acetyl moiety to the lysine residue of substrate proteins. We identified one lysine acetylated site (K158) in the acetyltransferase protein (A2ZVA8) (**Table 1**), which is not yet characterized. Previously, it has been shown that P/CAF, a histone acetyltransferase enzyme, is self-acetylated in humans [40]. Acetylation of P/CAF enhances its acetyltransferase activity, suggesting that acetylation of acetyltrasferases may also regulate the acetylation activity in rice.

#### 5) Lysine acetylation of hypothetical function unknown proteins

Among the lysine acetylated proteins, the hypothetical proteins with unknown function were a major group, including 10 proteins with 12 lysine-acetylated sites (**Table 1**). Further investigations on the molecular functions of these proteins are required to fully appreciate the function of protein acetylation in the control of cellular activities.

## Discussion

### 1. The Size of Rice Acetylome

Lysine acetylation has been shown to be a widespread protein modification from *E. coli* to human cells [1], [10]–[12]. It was first described in histones, but we now know that it targets a wide range of proteins, particularly the proteins involved in metabolism and protein complexes [1]. In spite of the genome-wide examination of acetylomes in multiple organisms, the precise acetylome size and the regulatory mechanism of protein acetylation are still largely unknown for non-histone proteins, particularly in plants. Wu *et al*., 2011 [21] identified sixty-four lysine modification sites in 57 proteins in *Arabidopsis*. Meanwhile, Finkermeier *et al*., 2011 [20] identified 91 lysine acetylation sites on 74 proteins. Melo-Braga identified 138 acetylation sites in grapewine [22]. In our studies in rice, we identified 60 lysine acetylated sites on 44 proteins. The identification of the acetylated sites and proteins has substantially advanced our understanding on protein acetylation in plants and their role in cellular activities. It is evident that protein acetylation plays a much broader role than merely regulation of histone functions. Interestingly, we found that only three out of the 44 acetylated proteins in our list were in common with those reported in *Arabidopsis* studies. These three proteins were cytochrome P450, an armadillo repeat-containing protein, and a glyceraldehyde-3-phosphate dehydrogenase protein. Nuclear proteins were the largest group in our identified proteins. However, we identified very few photosynthesis proteins probably because we used dark grown suspension cells as protein source. In contrast, proteins involved in photosynthesis represented a significant proportion of the total observed acetylated proteins in the two reports in *Arabidopsis* [20], [21]. These studies suggested that each of the three reports identified only a small fraction of a large plant acetylome. Analysis of the acetylated histone peptides identified in these studies strongly supports this conclusion. In *Arabidopsis*, about 15 acetylation sites have been reported in the four core histones [41]. Finkemeier *et al*., 2011 [20] identified two acetylation sites in H2B, one site on H3 and three sites on H4. Wu *et al*., 2011 [21] identified one acetylation site on H3 and two sites on H4. In this study, we identified two acetylation sites on H4, two sites on H2B, and two sites on H2A. The low coverage of the core histone modification sites in these three studies clearly suggests that a large portion of the plant acetylome has not been discovered. In human cells, 3600 lysine acetylation sites on 1750 proteins were identified [1], including all the known acetylated histone sites. In this study, they further separated the peptides from immunoaffinity purification by means of isoelectric focusing the peptides into 12 fractions and made use of diverse lysine deacetylase inhibitors. These methods can also be used to further investigate the plant acetylome. In addition, mutants of the lysine deacetylase genes can be very helpful as well. Meanwhile, it is worth to note that protein biochemistry studies in plants have always been a challenge compared with other organisms. For example, tandem affinity protein purification has become a very successful routine protein purification method in many organisms. In plants, however, proteins successfully purified with this approach are still very limited [42]–[51]. This could be due to cell wall effect on protein extraction or the plant secondary compounds that may interfere with affinity purification.

To overcome the problem in plants, different approaches should be used. Further separation of peptides from immunoaffinity purification by means of isoelectric focusing into multiple fractions and make use of diverse lysine deacetylase inhibitors and mutants may lead to a better coverage of the lysine acetylome in plants [1].

### 2. Comparison of Lysine Acetylation in Different Organisms

Even though lysine acetylation first identified for histone proteins, now it is considered as widespread modification for different non histone proteins. Recent global proteomic analysis studies further extended the scope of lysine acetylation in different organisms such as E. coli, salmonella, mouse, human, and Drosophila [1], [10]–[12], [20]–[22], [52]–[57]. Most of the large scale acetylation studies are reported in bacteria, mouse and human cell lines. In plants, only Arabidopsis and grape vine have been studied [20]–[22]. Little is known about the lysine acetylation beyond the histone proteins in major crop plants such as rice, maize, wheat and sorghum. Our results present an example of protein acetylation in monocots. We summarized all the published protein acetylation data and our rice results in Table S4 for the convenience of comparison.

### 3. The Features of the Acetylation Sites in Rice

We examined the protein sequence 20 amino acids upstream and 20 amino acids downstream of the acetylation sites in details (**Table S2** and **Fig. 3A**). Although a clear consensus sequence was not discovered, sequence bias around the acetylation site was evident. Interestingly, the amino acid bias shares common features with *Arabidopsis* and human cells to a certain degree. In *Arabidopsis*, only the amino acids in four positions around the acetylation sites were examined [20]. In the −1 position, the top five amino acids in terms of frequency of occurrence were G, A, D, S, and E. In rice, this order was the following: A, E, G, S, and R. In the −2 position, the top five amino acid presence order was G, L, A, E, and T and in rice it was the following: A, G, L, E, and R. The results clearly indicate that rice and Arabidopsis share high degree of similarity in amino acid preference. Compared with human cells [1], the rice acetylation sites share little similarity in positions from −3 to +2. Interestingly, however, from position −4 to −6 and +3 to +6 both human and rice cells have similar preference to charged amino acids K, R, and D. Our further analysis showed that the bias to these three charged amino acids extend to −20 and +20 in both directions (**Table S2**). Other preferred amino acids include A, G, L, S, and T. Interestingly, the charged amino acid histidine is well underrepresented in sites around the acetylated lysine. Other underrepresented amino acids include W, Y, and F. The distribution of the acetylated sites among local secondary structure was about 52% in coil, 39% in helix, and 9% in beta-strand (**Fig. 3B** and **Table S3**), which was similar to the distribution in human cells [1]. Once more comprehensive analysis of the amino acid sequences of acetylated proteins is completed, the feature of the acetylation sites will be revealed.

### 4. Diverse Function of the Acetylated Proteins

The acetylated proteins identified in our studies are involved in diverse molecular processes with wide biological functions. Nuclear proteins represent 17.2% of the acetylated proteins. These proteins regulate genome stability (transposon and retrotransposon proteins), transcription (transcription factors and histone modifications), and genome reverse transcription (gag-pol protein). Protein involved in translational activity is also subjected to lysine acetylation, which include translation initiation factor IF-3-like protein (Q6K674).

Another important group of acetylated proteins we detected are metabolic proteins, which includes glyceraldehyde-3-phosphate dehydrogenase (Q7FAH2), enolase (B9G3A0), cytochrome P450 72A1 (Q8L4Q4), and dihydroorotate dihydrogenase (Q6Z744) (**Table 1** and **Fig. 6**). Glyceraldehyde 3-phosphate dehydrogenase (GAPDH) is a key enzyme in glycolysis pathway. This enzyme is also lysine acetylated in *Arabidopsis* and *E. coli* [11], [20]. Enolase is another glycolysis pathway enzyme that was shown to be lysine acetylated in both our study and *E. coli* [11]. The fact that these and other glycolytic enzymes were shown to be acetylated in bacteria, mammals and plants suggests that functional regulation of acetylation in glycolysis might be conserved. Cytochrome P450 proteins are heme-thiolate proteins that play a key role in biosynthesis of lignins, terpenoids, alkaloids, sterols, and fatty acids. They are also involved in the herbicide detoxification, phytoalexin synthesis, pigment biosynthesis, and phytohormone synthesis in plants [58]. In *Arabidopsis*, cytochrome P450 (At5g45340) is also lysine acetylated, suggesting that cytochrome acetylation is conserved in plants. We identified two lysine acetylation sites in armadillo repeat-containing protein. Armadillo family proteins are involved in many functions like cell death, cell division and cytoskeleton organization in many plants. An *Arabidopsis* protein from this group, armadillo repeat-containing kinesin related protein, has also been shown to be acetylated, indicating that acetylation may play a role in the regulation of this protein group [20].

Lysine acetylation is known to have a negative crosstalk with ubiquitination and sumoylation, therefore constituting a regulatory switch controlling protein stability and function [59], [60]. Effects of acetylation on E3 ubiquitin ligase activity in human and animals have already been documented, such as acetylation-mediated inhibition of Mdm2, a ubiquitin E3 ligase and an important negative regulator of p53 [61]. Similar to the ubiquitination, SUMO (Small Ubiquitin-like Modifier) proteins modify their targets in the process called sumoylation, regulating their stability, localization and transcription. SUMO proteins are first activated by an E1 enzyme, followed by conjugation via E2 enzyme, and finally they are ligated to the ε-amino group of lysine residue in the target protein by the E3 ligase [62]. In this study, we found that E3 SUMO-protein ligase SIZ2 was lysine acetylated (**Table 1** and **Fig. 6**). Our results provide a basis for further mechanistic studies of the regulatory mechanism of SUMO E3 ligase activity in rice. Interestingly, we also found that a protein exosome complex exonuclease is lysine acetylated in rice. Exoribonuclease complex is involved in the degradation of unstable mRNAs containing AU-rich elements (AREs) within their 3′-untranslated regions, implying a possible role of lysine acetylation in the degradation of mRNAs as well.

In summary, our global proteomic study of lysine acetylation has revealed that this modification is much more abundant in rice than previously anticipated, and it targets a large group of non-histone proteins with broad biological functions. Several of the acetylated proteins identified in our studies are also acetylated in *Arabidopsis* or other organisms. Our results suggest that lysine acetylation is a highly conserved modification, constituting a common regulatory mechanism in the control of cellular activities.

## Supporting Information

Figure S1 **Fragmentation spectra of lysine acetylated peptides identified in rice.**
(PDF)

Table S1 **Summary of lysine acetylated peptides identified in rice.**
(XLS)

Table S2 **Amino acid frequency percentages for ±20 amino acids from lysine acetylated site.**
(XLS)

Table S3 **Detailed secondary structural characteristics of acetylated lysines identified in rice.**
(XLS)

Table S4 **Comparison of lysine acetylated sites (Ac sites) and acetylated proteins (Ac proteins) in different organisms.**
(PDF)
