# Supplementary material for: Global Analysis of Lysine Acetylation Suggests the Involvement of Protein Acetylation in Diverse Biological Processes in Rice (Oryza sativa)
Source: PLoS One. 2014 Feb 20;9(2):e89283. doi: 10.1371/journal.pone.0089283 (PMC3930695; doi:10.1371/journal.pone.0089283)
Supplement: Figure S1 — Fragmentation spectra of lysine acetylated peptides identified in rice. (PDF) [file pone.0089283.s001.pdf]

Supplementary Figure 1

AEK(ac)KPAAK(ac)KPAEEEPAAEK(ac)APAAGKKPK

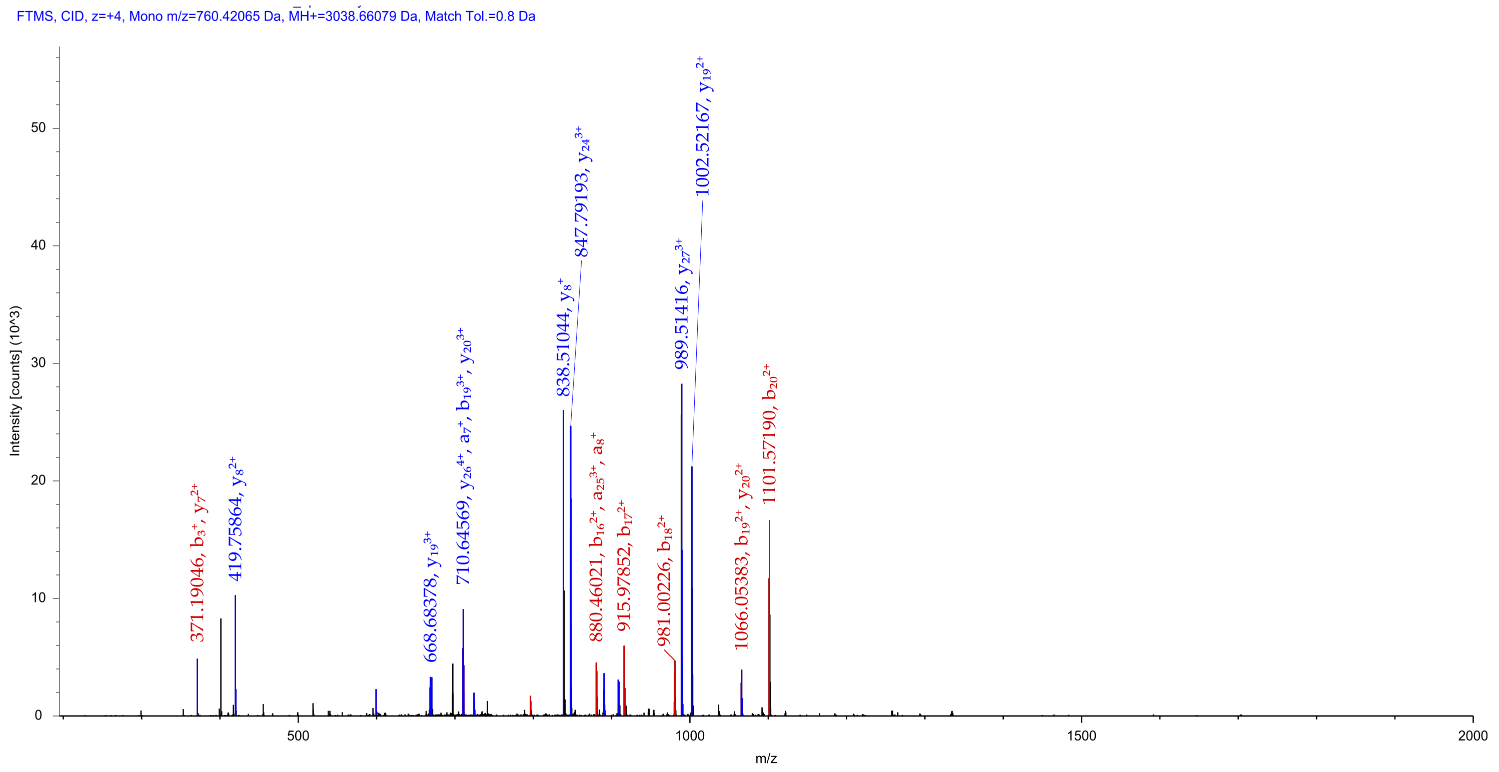

M/Z	Intensity	Matches
796.415	1717.3	b (14) (2+)
880.4602	4551.4	b (16) (2+), a (25) (3+), a (8) (1+)
915.9785	5980.6	b (17) (2+)
981.0023	4695.6	b (18) (2+)
1101.572	16667	b (20) (2+)
890.4898	3633.4	b (25) (3+), b (8) (1+)-H2O, y (25) (3+)
668.3492	2431.8	b (25) (4+), y (25) (4+)
908.5147	3091	b (8) (1+), y (17) (2+)-H2O
890.8243	3030.9	b (8) (1+)-NH3
724.412	1973	y (14) (2+), a (20) (3+), y (7) (1+)-NH3, b (27) (4+)
1002.522	21232.9	y (19) (2+)
668.6838	3328.7	y (19) (3+)
1066.054	3942.6	y (20) (2+), b (19) (2+)
847.7919	24673	y (24) (3+)
710.6457	9080.4	y (26) (4+), y (20) (3+), b (19) (3+), a (7) (1+)
989.5142	28262.3	y (27) (3+)
670.4211	3294.2	y (6) (1+)
371.1905	4876.4	y (7) (2+), b (3) (1+)
838.5104	26031.6	y (8) (1+)
419.7586	10277.3	y (8) (2+)
909.5458	2913.3	y (9) (1+), y (17) (2+)-NH3

AGFLK(ac)HNLWVTSYK

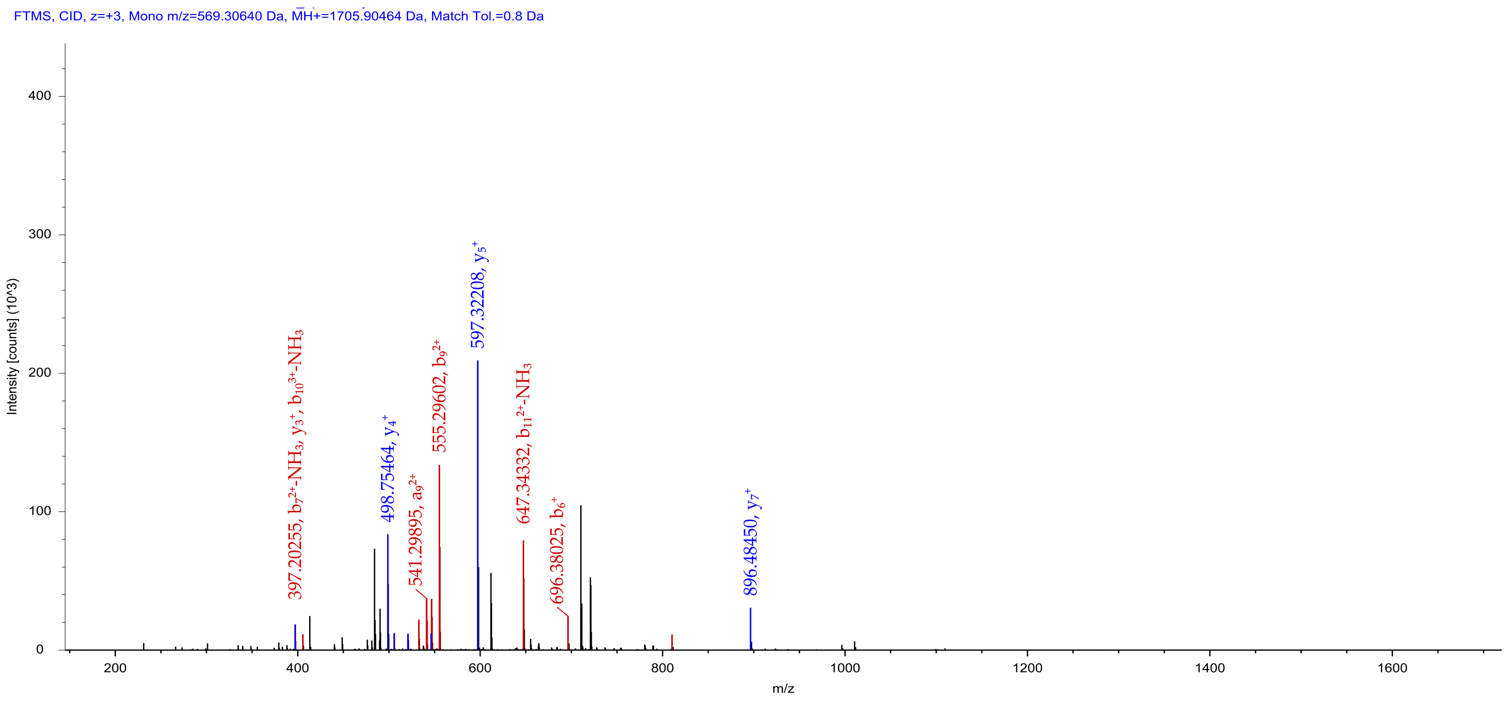

M/Z	Intensity	Matches
647.3433	79308.3	b (11) (2+)-NH3
520.9315	11768.2	b (13) (3+), y (12) (3+)-H2O, y (12) (3+)-NH3
541.8004	21451	b (5) (1+)-NH3
696.3803	24464.1	b (6) (1+)
810.4227	11236.5	b (7) (1+)
405.7151	11435.7	b (7) (2+)
555.296	133718.7	b (9) (2+)
546.7835	37018.6	b (9) (2+)-NH3
546.2912	11982.8	y (13) (3+)
397.2026	18453.6	y (3) (1+), b (10) (3+)-NH3, b (7) (2+)-NH3
498.7546	83703.2	y (4) (1+)
597.3221	209178.5	y (5) (1+)
896.4845	30549.4	y (7) (1+)

AK(ac)DLLECINHVQ

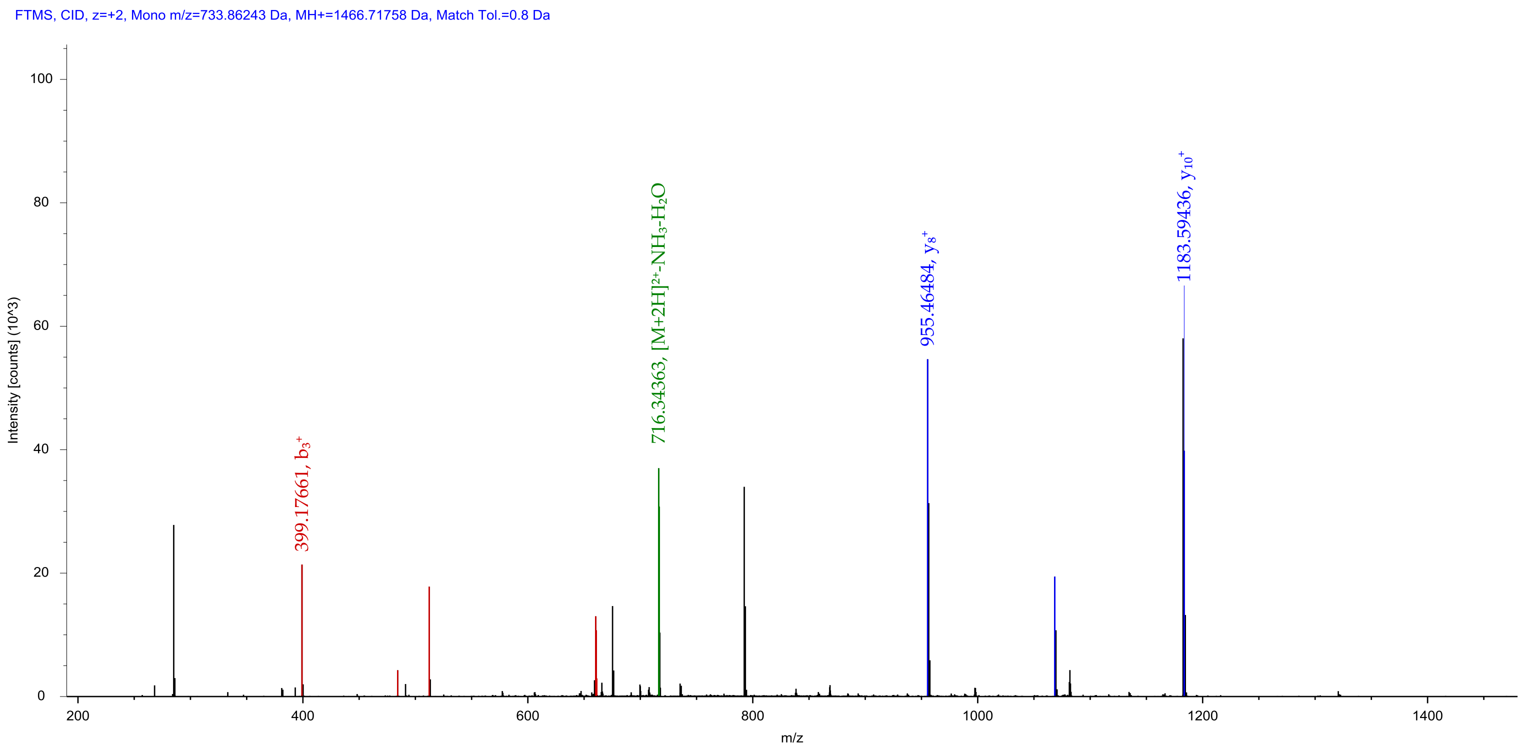

M/Z	Intensity	Matches
660.3288	13002.2	b (11) (2+)
399.1766	21385.5	b (3) (1+)
512.2603	17797.3	b (4) (1+)
1183.594	39828.5	y (10) (1+)
955.4648	54656.8	y (8) (1+)
1068.549	19436.4	y (9) (1+)

AK(ac)VIAEPTATDK(ac)GK

FTMS, CID, z=+3, Mono m/z=518.94690 Da, MH+=1554.82614 Da, Match Tol.=0.8 Da

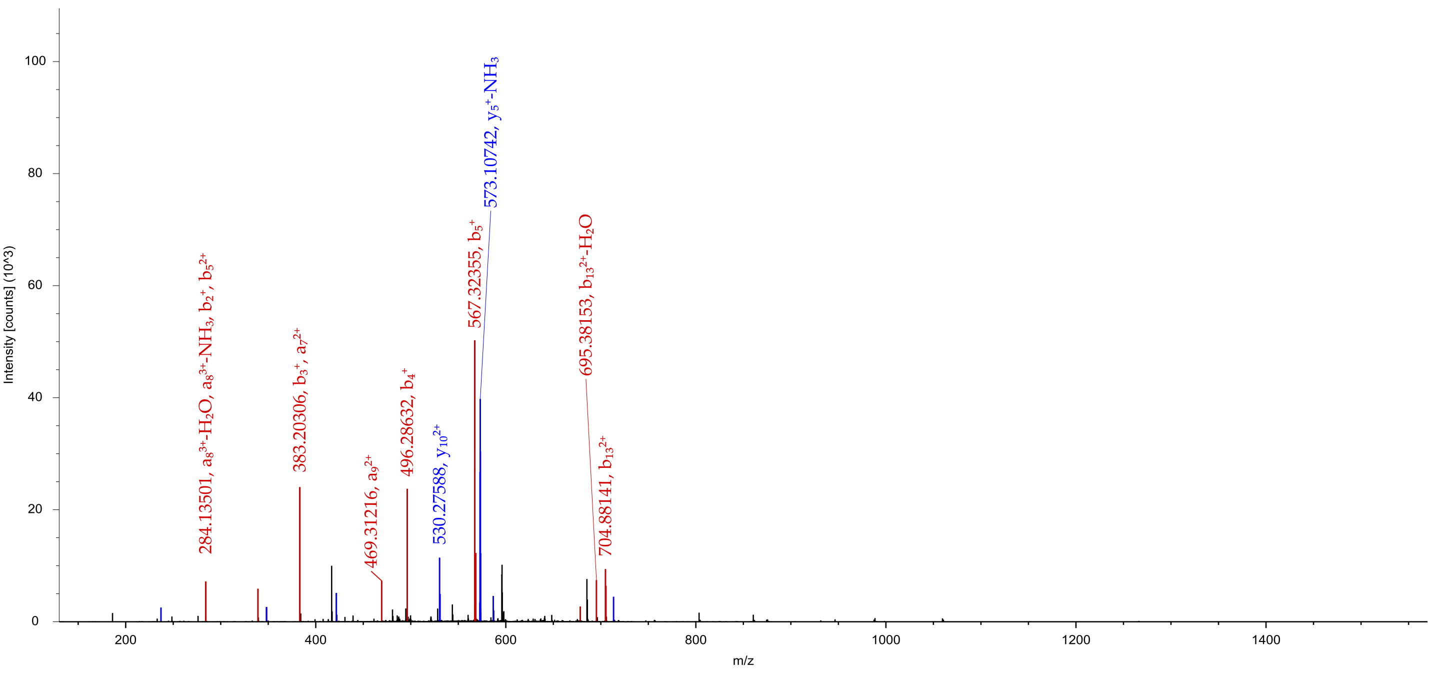

M/Z	Intensity	Matches
704.8814	9388	b (13) (2+)
695.3815	7423.6	b (13) (2+)-H2O
383.2031	24015.9	b (3) (1+), a (7) (2+)
496.2863	23734.3	b (4) (1+)
567.3236	50237.8	b (5) (1+)
678.3547	2708.7	b (6) (1+)-H2O
339.1896	5886.8	b (6) (2+)-H2O
530.2759	11434.6	y (10) (2+)
348.1947	2638.5	y (10) (3+)-NH3, y (10) (3+)-H2O, b (6) (2+)
586.8168	4594	y (11) (2+)
713.3803	4455.4	y (13) (2+)-NH3
237.1342	2528.6	y (4) (2+)-NH3
572.9073	26766.7	y (5) (1+)-H2O
573.1074	39754.9	y (5) (1+)-NH3
421.7288	5140.7	y (8) (2+)-H2O, y (8) (2+)-NH3

CKLGSLGK(ac)PNEPSR

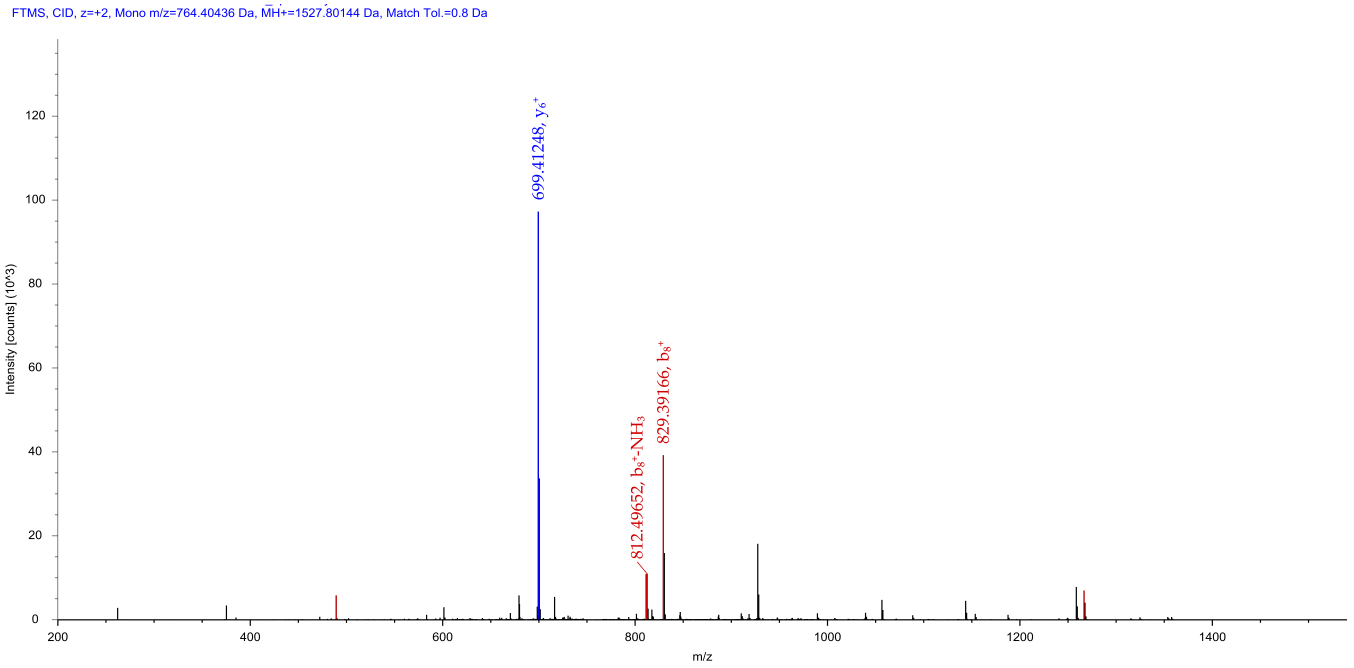

M/Z	Intensity	Matches
1266.654	6994	b (12) (1+)
489.2766	5817	b (5) (1+)
829.3917	39202.3	b (8) (1+)
811.3812	10871.3	b (8) (1+)-H2O
812.4965	10981.6	b (8) (1+)-NH3
699.4125	97268.1	y (6) (1+)

CTTPK(ac)TLKWDEITLPEK(ac)

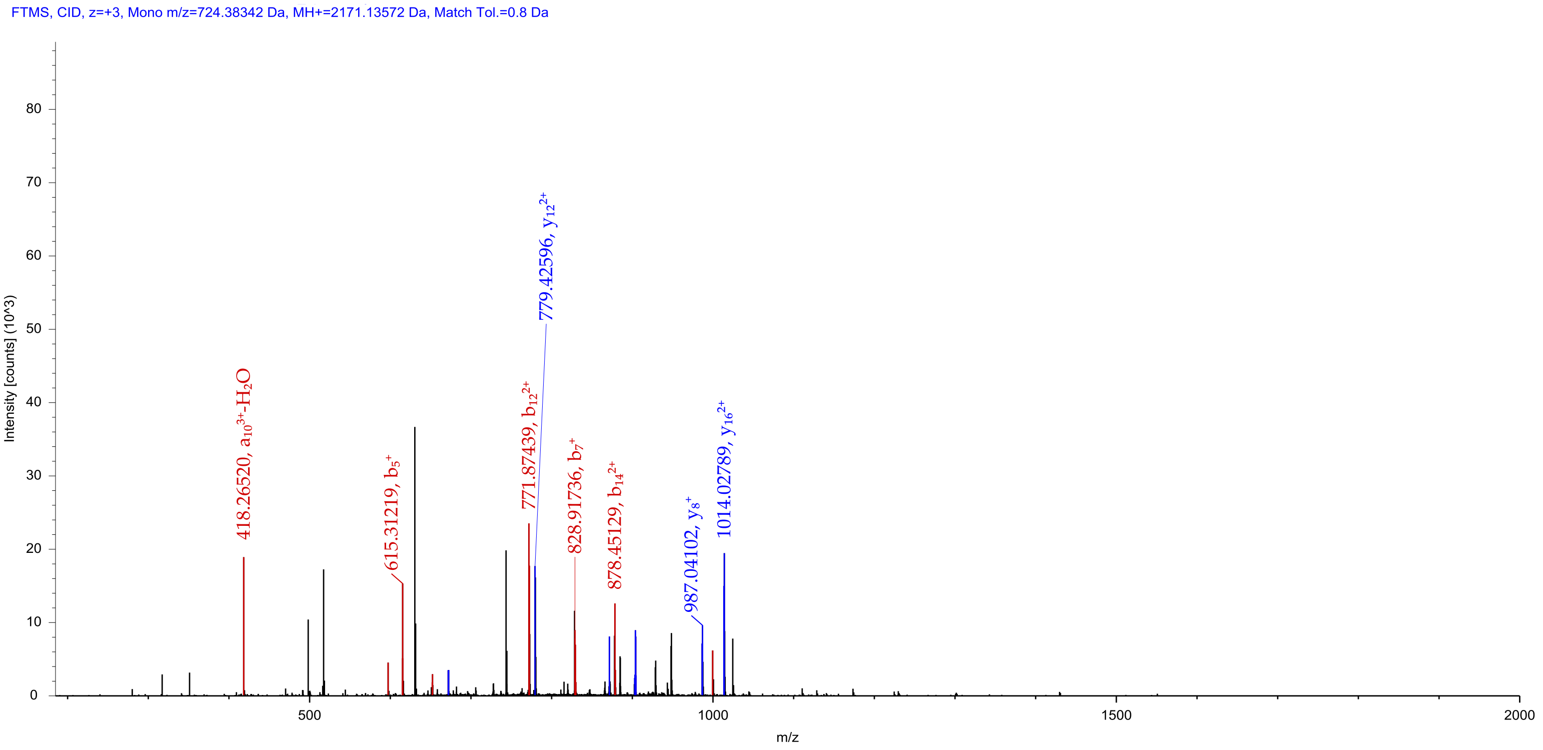

M/Z	Intensity	Matches
771.8744	23531.6	b (12) (2+)
878.4513	12609.5	b (14) (2+)
615.3122	15326.1	b (5) (1+)
597.3017	4539.6	b (5) (1+)-H2O
828.9174	8961.4	b (7) (1+)
999.6181	6198.4	b (8) (1+)
672.3351	3528.6	y (10) (2+)
779.426	17703.2	y (12) (2+)
903.8045	8944.2	y (14) (2+)-H2O, a (15) (2+)-H2O, y (14) (2+)-NH3
1014.028	19466.7	y (16) (2+)
871.5585	8090.1	y (7) (1+)
987.041	9623.5	y (8) (1+)

DLTNPYFAHLLGKGLVLIDGDEWK(ac)RHYKVVHPAFDMDK(ac)

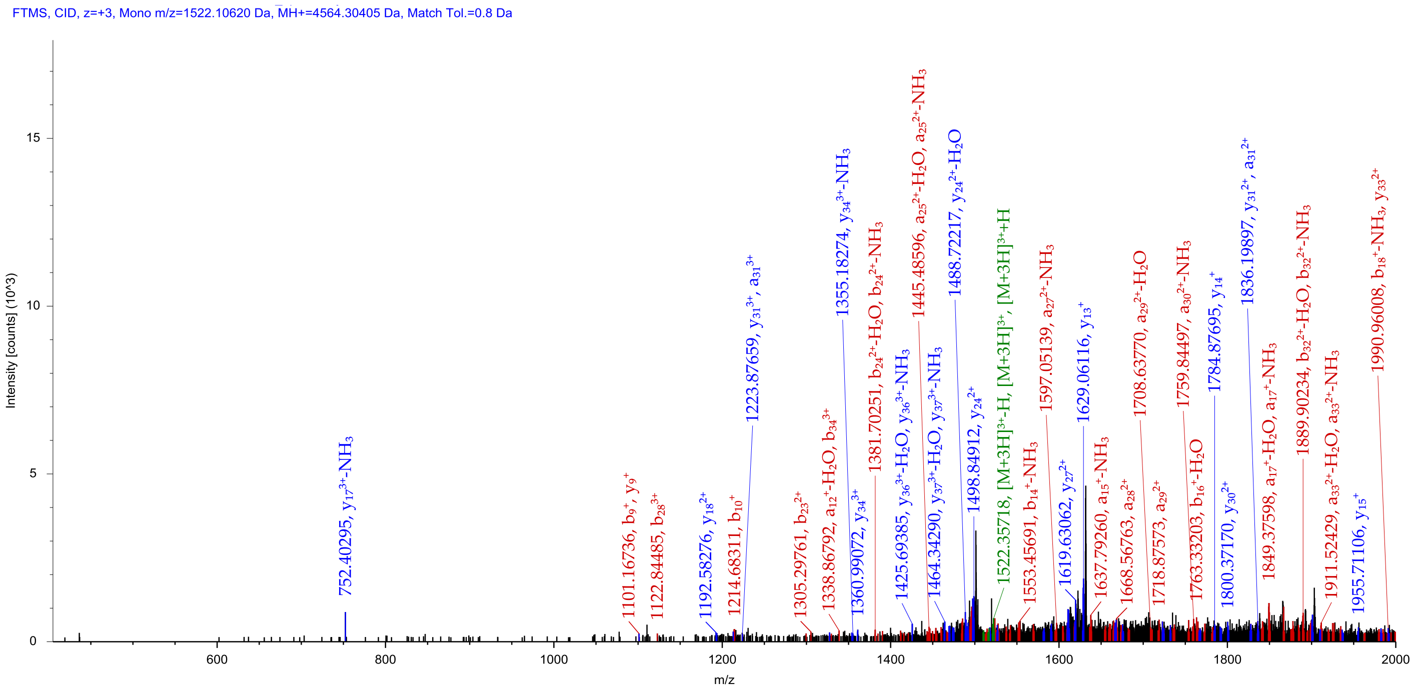

M/Z	Intensity	Matches
1214.683	374.1	b (10) (1+)
1327.546	276.3	b (11) (1+), y (33) (3+), y (21) (2+)-NH3
1384.941	242.7	b (12) (1+)
1513.538	293.6	b (13) (1+)
1494.478	665.3	b (13) (1+)-H2O
1496.347	1045.3	b (13) (1+)-NH3
1569.545	525.5	b (14) (1+)
1552.268	575.5	b (14) (1+)-H2O
1553.457	619.7	b (14) (1+)-NH3
1664.88	631.2	b (15) (1+)-H2O
1666.058	562.1	b (15) (1+)-NH3
1782.52	433.7	b (16) (1+), b (30) (2+)
1763.332	725.9	b (16) (1+)-H2O
1765.026	604.1	b (16) (1+)-NH3
1895.723	345.4	b (17) (1+)
1877.654	404.1	b (17) (1+)-H2O
1877.885	615.9	b (17) (1+)-NH3
1990.834	272.4	b (18) (1+)-H2O
1305.298	295.8	b (23) (2+)
1390.248	318.7	b (24) (2+)
1381.703	357.3	b (24) (2+)-NH3, b (24) (2+)-H2O
1469.218	468.2	b (25) (2+)
1459.848	430.6	b (25) (2+)-H2O, b (37) (3+), b (25) (2+)-NH3
1537.274	453.8	b (26) (2+)
1527.609	544.5	b (26) (2+)-H2O
1528.216	362.3	b (26) (2+)-NH3
1618.921	617.1	b (27) (2+)
1609.764	566.8	b (27) (2+)-H2O
1682.361	397.2	b (28) (2+), b (15) (1+)
1673.65	500.6	b (28) (2+)-H2O, b (28) (2+)-NH3
1122.845	246.4	b (28) (3+)
1732.286	496.4	b (29) (2+), y (29) (2+)

1724.317	409.2	b (29) (2+)-NH3, y (29) (2+)-NH3
1773.267	537.4	b (30) (2+)-H2O, b (30) (2+)-NH3
1849.877	875.5	b (31) (2+)
1841.713	608.9	b (31) (2+)-H2O, b (31) (2+)-NH3
1899.546	631.9	b (32) (2+)
1889.902	860.2	b (32) (2+)-NH3, b (32) (2+)-H2O
1934.746	462.2	b (33) (2+)
1924.919	555.5	b (33) (2+)-H2O
1926.733	559.5	b (33) (2+)-NH3
1999.156	299.3	b (34) (2+)-NH3, b (34) (2+)-H2O
1420.974	269	b (36) (3+)
1415.666	260	b (36) (3+)-NH3, b (36) (3+)-H2O
1453.707	410.8	b (37) (3+)-NH3, b (37) (3+)-H2O
1491.981	565.8	y (12) (1+)
1473.586	430.3	y (12) (1+)-H2O
1475.137	423.6	y (12) (1+)-NH3
1629.061	1883.8	y (13) (1+)
1611.498	945.6	y (13) (1+)-NH3
1784.877	517.9	y (14) (1+)
1766.446	341.3	y (14) (1+)-H2O
1955.711	428.7	y (15) (1+)
1936.238	361.4	y (15) (1+)-H2O
1937.413	272.7	y (15) (1+)-NH3
752.403	885.3	y (17) (3+)-NH3
1192.583	274.3	y (18) (2+)
1213.089	302.9	y (19) (2+)-NH3, b (22) (2+), y (19) (2+)-H2O
1498.849	1362.9	y (24) (2+)
1488.722	884.8	y (24) (2+)-H2O
1489.598	655.7	y (24) (2+)-NH3
1526.743	502.4	y (25) (2+)
1517.886	426.1	y (25) (2+)-NH3, y (25) (2+)-H2O
1590.928	536.8	y (26) (2+)
1582.322	398.4	y (26) (2+)-H2O, y (26) (2+)-NH3
1619.631	1244.1	y (27) (2+)
1610.629	967.9	y (27) (2+)-H2O, b (27) (2+)-NH3, y (27) (2+)-NH3, y (13) (1+)-H2O
1675.516	522	y (28) (2+)
1666.813	573.2	y (28) (2+)-H2O
1667.948	655.1	y (28) (2+)-NH3
1722.834	604.3	y (29) (2+)-H2O, b (29) (2+)-H2O
1800.372	595	y (30) (2+)
1791.334	452	y (30) (2+)-H2O
1792.364	416.1	y (30) (2+)-NH3
1827.883	530.5	y (31) (2+)-H2O, a (31) (2+)-H2O, a (31) (2+)-NH3, y (31) (2+)-NH3
1910.715	369	y (32) (2+)
1901.08	805	y (32) (2+)-H2O, y (32) (2+)-NH3
1990.96	407.7	y (33) (2+), b (18) (1+)-NH3
1982.457	397.3	y (33) (2+)-H2O, y (33) (2+)-NH3
1360.991	359.5	y (34) (3+)
1353.868	273.3	y (34) (3+)-H2O
1355.183	245.9	y (34) (3+)-NH3
1425.694	533.5	y (36) (3+)-NH3, y (36) (3+)-H2O
1470.335	476.5	y (37) (3+)
1464.343	598.5	y (37) (3+)-H2O, y (37) (3+)-NH3
1101.167	242.4	y (9) (1+), b (9) (1+)

EGNMEEFLEEVK(ac)ERLK(ac)KELK

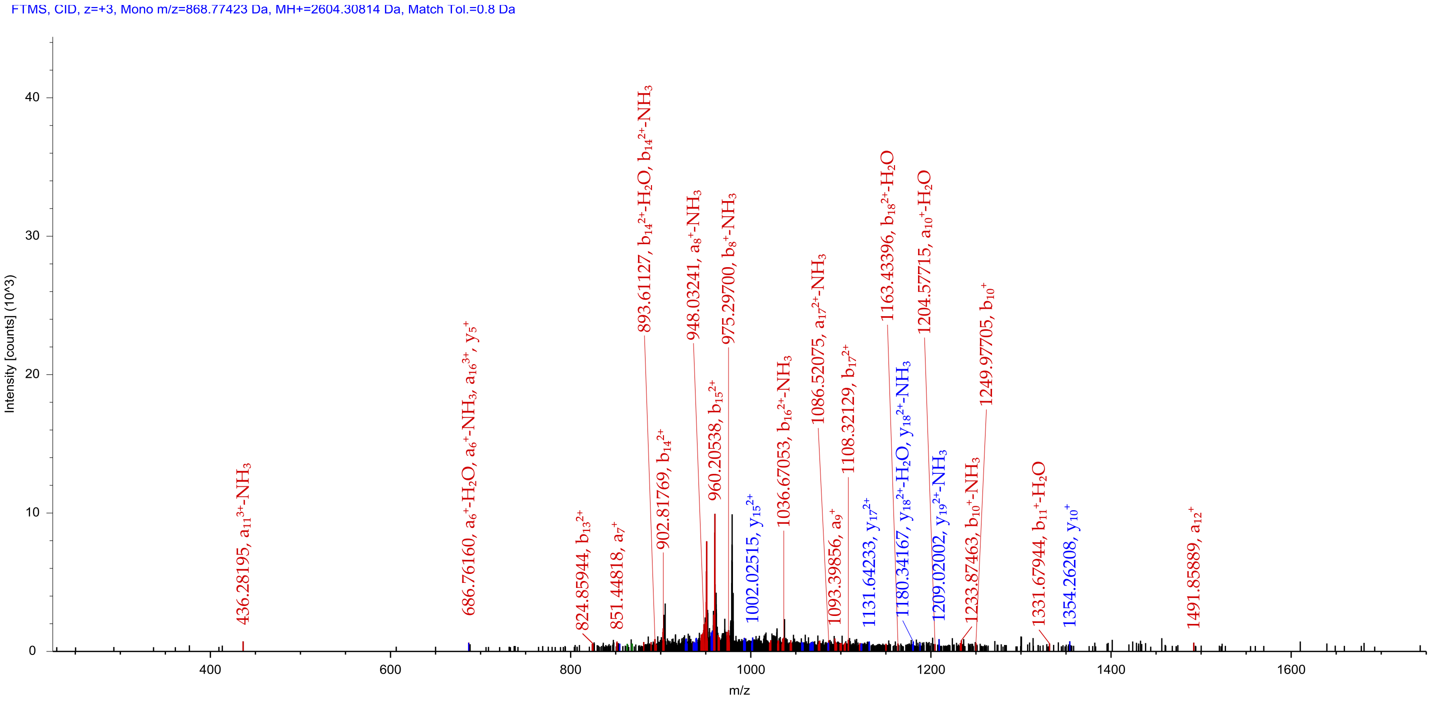

M/Z	Intensity	Matches
1249.977	611.6	b (10) (1+)
1232.786	611.9	b (10) (1+)-H2O
1233.875	801.9	b (10) (1+)-NH3
1331.679	571	b (11) (1+)-H2O
824.8594	559.1	b (13) (2+)
902.8177	1668.6	b (14) (2+)
893.6113	884.9	b (14) (2+)-NH3, b (14) (2+)-H2O
960.2054	9945	b (15) (2+)
950.9551	7957.3	b (15) (2+)-NH3, b (15) (2+)-H2O
1044.502	808.3	b (16) (2+)
1035.393	926.1	b (16) (2+)-H2O
1036.671	1010.1	b (16) (2+)-NH3
1108.321	837.7	b (17) (2+)
1100.074	608.1	b (17) (2+)-H2O
1100.735	611.5	b (17) (2+)-NH3
1163.434	568.4	b (18) (2+)-H2O
992.9761	977.3	b (8) (1+), y (15) (2+)-NH3, y (15) (2+)-H2O
973.6243	1413.1	b (8) (1+)-H2O
975.297	1538.8	b (8) (1+)-NH3
1121.309	604.1	b (9) (1+)
1103.856	544.6	b (9) (1+)-H2O
1104.729	634.4	b (9) (1+)-NH3
1354.262	732.6	y (10) (1+)

854.4824	553.6	y (13) (2+)-H2O, y (13) (2+)-NH3
928.2129	939.1	y (14) (2+)-H2O, y (14) (2+)-NH3
1002.025	1011.2	y (15) (2+)
1066.461	605.6	y (16) (2+)
1056.724	690.9	y (16) (2+)-H2O
1057.9	637.5	y (16) (2+)-NH3
1131.642	721.3	y (17) (2+)
1123.084	583.4	y (17) (2+)-NH3, y (17) (2+)-H2O
1187.956	608.8	y (18) (2+)
1180.342	712.4	y (18) (2+)-NH3, y (18) (2+)-H2O
1209.02	883.7	y (19) (2+)-NH3
686.7616	623	y (5) (1+), a (6) (1+)-H2O, a (6) (1+)-NH3, a (16) (3+)
957.3701	1506.8	y (7) (1+)
939.2016	925.4	y (7) (1+)-H2O
939.6118	979.2	y (7) (1+)-NH3
1086.058	641.7	y (8) (1+), a (17) (2+)-H2O
1067.17	683.9	y (8) (1+)-H2O
1068.604	705.3	y (8) (1+)-NH3

EK(ac)NFWARHSTSCSPMPGK

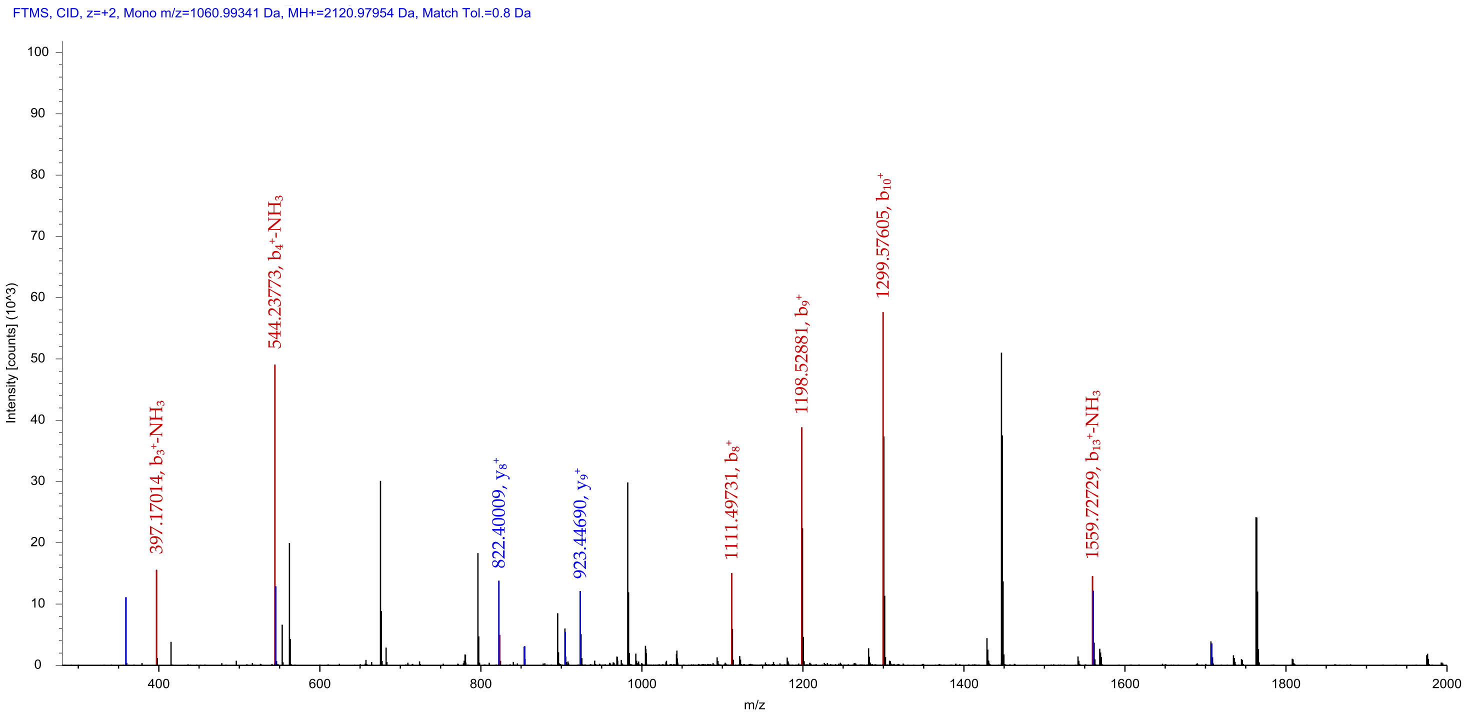

M/Z	Intensity	Matches
1299.576	57636.7	b (10) (1+)
1559.727	14554.1	b (13) (1+)-NH3
397.1701	15591.7	b (3) (1+)-NH3
544.2377	49072.6	b (4) (1+)-NH3
1111.497	15053	b (8) (1+)
1198.529	38845.9	b (9) (1+)
1560.731	12164.7	y (14) (1+)
1707.798	3568.4	y (15) (1+)
854.4025	3110.1	y (15) (2+)
545.241	12895.4	y (5) (1+)
359.1911	11095.1	y (7) (2+)-H2O, y (7) (2+)-NH3
822.4001	13806.4	y (8) (1+)
923.4469	12112.4	y (9) (1+)
904.9259	5423.4	y (9) (1+)-H2O

ELAMK(ac)FEKGLNTATLLSNEVK(ac)

FTMS, CID, z=+3, Mono m/z=826.76135 Da, MH+=2478.26950 Da, Match Tol.=0.8 Da

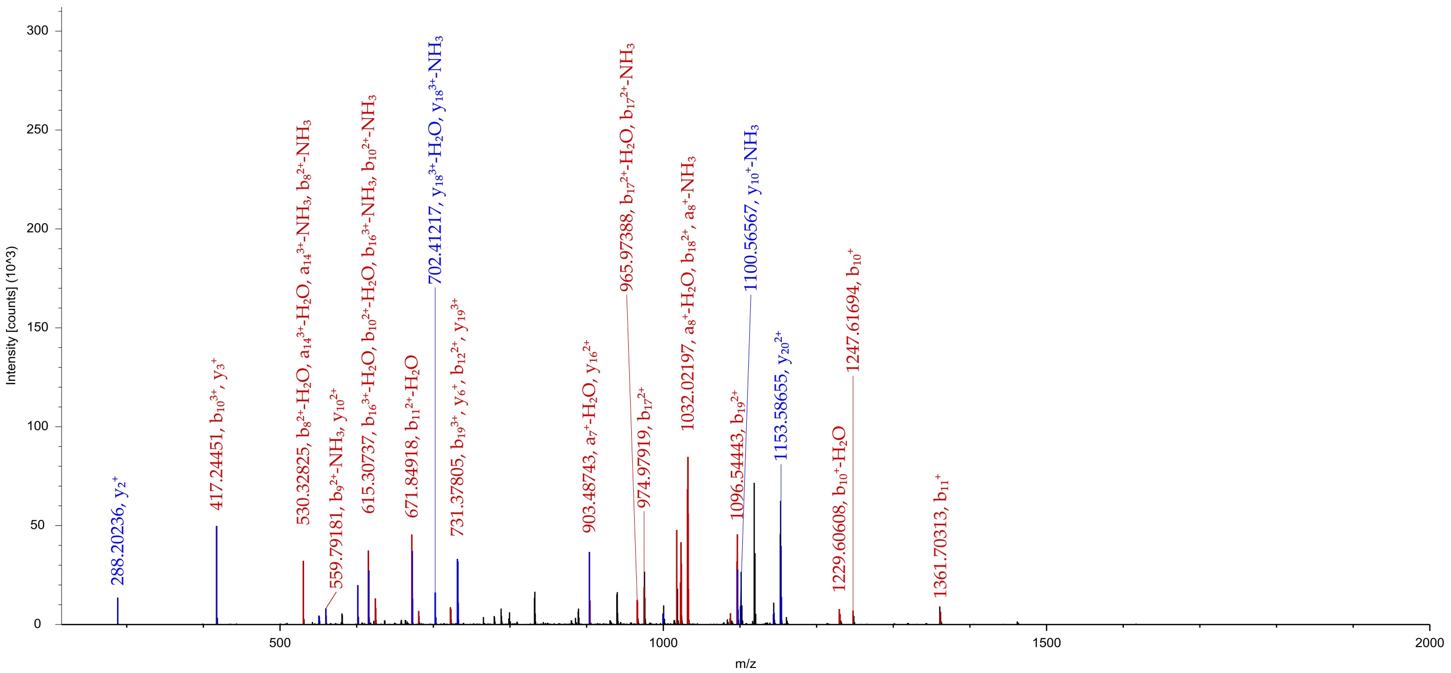

M/Z	Intensity	Matches
1247.617	7117.9	b (10) (1+)
1229.606	7806	b (10) (1+)-H2O
1230.609	5353.2	b (10) (1+)-NH3
624.3127	13232.4	b (10) (2+)
615.3074	37320.3	b (10) (2+)-H2O, b (16) (3+)-NH3, b (16) (3+)-H2O, b (10) (2+)-NH3
1361.703	6456.3	b (11) (1+)
680.8536	6890.4	b (11) (2+)
671.8492	45489.9	b (11) (2+)-H2O
722.8737	7819.2	b (12) (2+)-NH3
974.9792	18831.1	b (17) (2+)
965.9739	12467.4	b (17) (2+)-H2O, b (17) (2+)-NH3
1023.014	41572.6	b (18) (2+)-NH3, b (18) (2+)-H2O
1096.544	45610.6	b (19) (2+)
1087.539	5673.6	b (19) (2+)-NH3, b (19) (2+)-H2O
672.8516	13144.9	b (5) (1+)
559.7918	8119.7	b (9) (2+)-NH3, y (10) (2+)
1100.566	9292.8	y (10) (1+)-NH3
550.7869	4523.5	y (10) (2+)-NH3, y (10) (2+)-H2O
615.8082	27293.2	y (11) (2+)
702.4122	16109.4	y (18) (3+)-H2O, y (18) (3+)-NH3
1097.045	27720.1	y (19) (2+)
731.3781	33181.7	y (19) (3+), b (12) (2+), y (6) (1+), b (19) (3+)
288.2024	13553.8	y (2) (1+)
1153.587	39708.6	y (20) (2+)
1144.575	5955	y (20) (2+)-H2O
417.2445	49816.3	y (3) (1+), b (10) (3+)
999.5185	5551	y (9) (1+)-NH3

ELEEVVEYLK(ac)NPSK

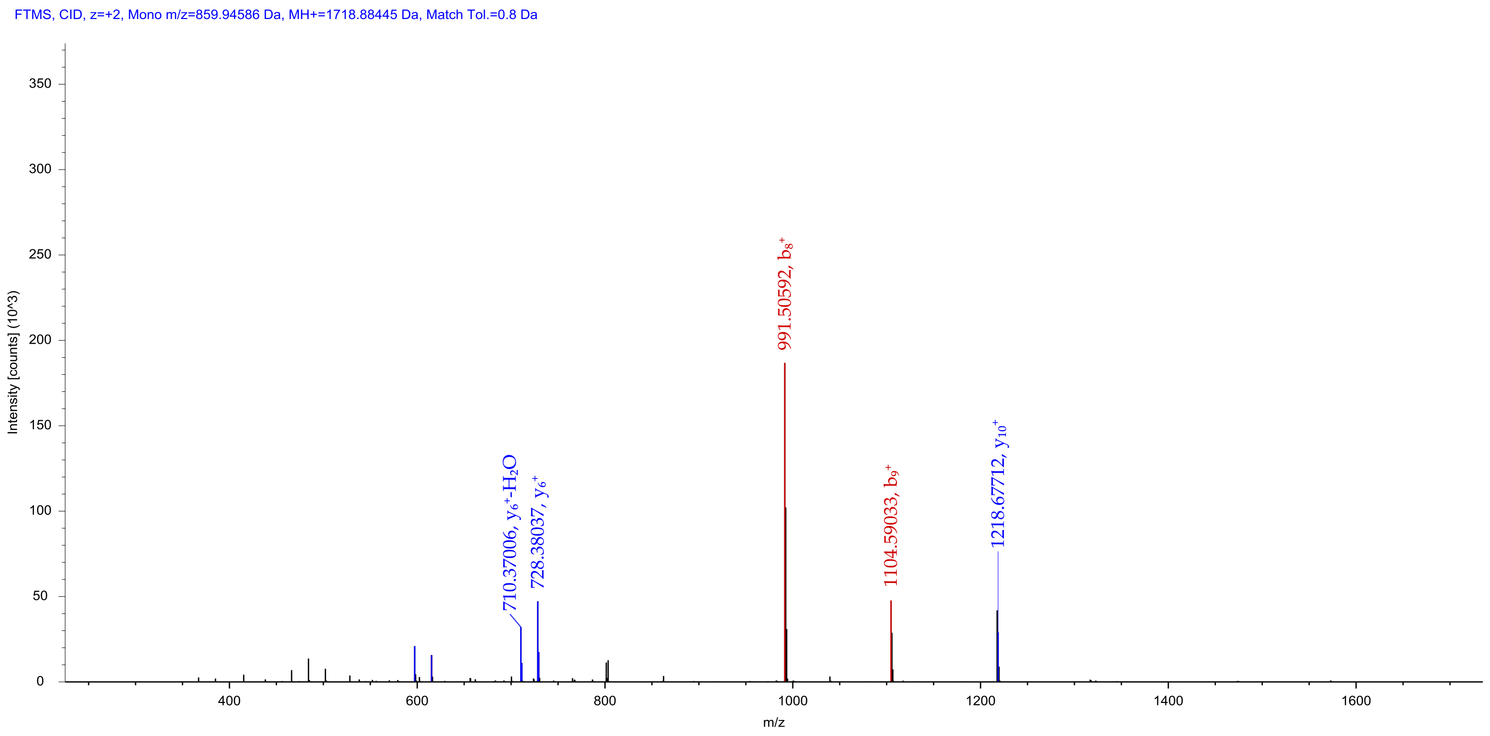

M/Z	Intensity	Matches
991.5059	186813.3	b (8) (1+)
1104.59	47700.7	b (9) (1+)
1218.677	29020.7	y (10) (1+)
615.2965	15710.6	y (5) (1+), a (10) (2+)-NH3, a (10) (2+)-H2O
597.2862	20960.3	y (5) (1+)-H2O
728.3804	47229.5	y (6) (1+)
710.3701	32117.7	y (6) (1+)-H2O
711.3729	11119.7	y (6) (1+)-NH3

ESDDMKLCK(ac)EDCISDCFAMEEDDMIK

FTMS, CID, z=+3, Mono m/z=1091.44019 Da, MH+=3272.30600 Da, Match Tol.=0.8 Da

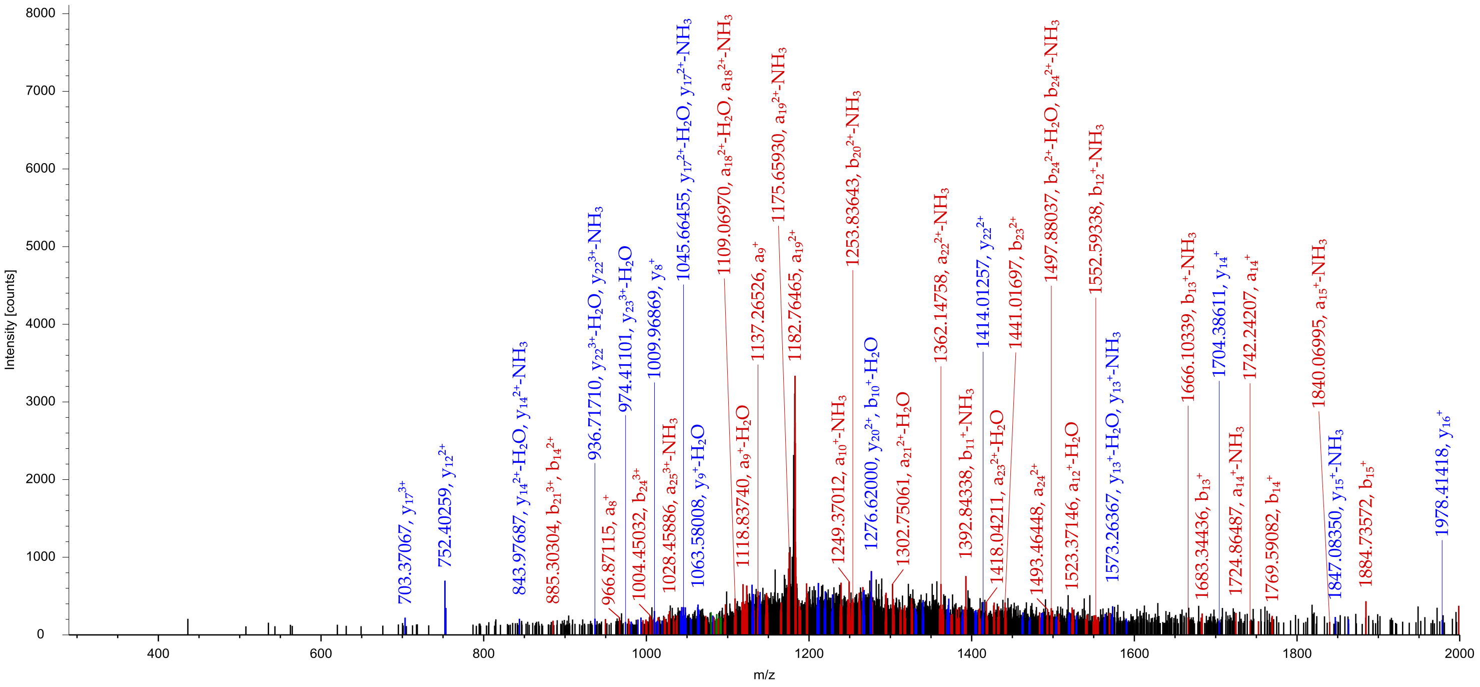

M/Z	Intensity	Matches
1294.757	542.2	b (10) (1+)
1276.62	821	b (10) (1+)-H2O, y (20) (2+)
1277.052	498.5	b (10) (1+)-NH3
1409.423	340.7	b (11) (1+)
1392.035	363.6	b (11) (1+)-H2O
1392.843	756.9	b (11) (1+)-NH3
1569.032	284.4	b (12) (1+)
1551.064	251.9	b (12) (1+)-H2O
1552.593	252.9	b (12) (1+)-NH3
1683.344	274.7	b (13) (1+)
1666.103	225.4	b (13) (1+)-NH3
1769.591	244.9	b (14) (1+)
1752.603	175.3	b (14) (1+)-NH3
1884.736	432.9	b (15) (1+)
1022.09	231.5	b (16) (2+)
1014.536	225	b (16) (2+)-H2O, b (16) (2+)-NH3
1097.152	394	b (17) (2+)
1087.35	204.3	b (17) (2+)-H2O
1088.532	213.2	b (17) (2+)-NH3
1131.438	526.1	b (18) (2+), y (18) (2+)-NH3

1123.435	634.2	b (18) (2+)-NH3, b (18) (2+)-H2O
1197.06	663.7	b (19) (2+)
1187.668	448.4	b (19) (2+)-H2O
1188.614	367.2	b (19) (2+)-NH3
1261.758	542.6	b (20) (2+)
1253.5	447.2	b (20) (2+)-H2O
1253.836	469.6	b (20) (2+)-NH3
1326.026	473.2	b (21) (2+)
1317.645	380.7	b (21) (2+)-NH3, b (21) (2+)-H2O
885.303	181.5	b (21) (3+), b (14) (2+)
1384.047	354.5	b (22) (2+)
1375.406	264.7	b (22) (2+)-H2O
1376.102	270	b (22) (2+)-NH3
1441.017	416.5	b (23) (2+)
1432.996	367	b (23) (2+)-NH3, b (23) (2+)-H2O
1506.941	272.9	b (24) (2+)
1497.88	343.1	b (24) (2+)-NH3, b (24) (2+)-H2O
1004.45	253.4	b (24) (3+)
999.3983	196.4	b (24) (3+)-H2O, a (16) (2+)-H2O, b (24) (3+)-NH3
1554.762	227.9	b (25) (2+)-NH3, b (25) (2+)-H2O
1043.516	356.5	b (25) (3+), y (25) (3+)-NH3
1037.52	250.1	b (25) (3+)-NH3, b (25) (3+)-H2O
978.1174	207.3	b (8) (1+)-NH3, b (8) (1+)-H2O
1165.599	419.5	b (9) (1+)
1147.859	538.4	b (9) (1+)-NH3, b (9) (1+)-H2O
1228.828	522.3	y (10) (1+)
1388.661	323.3	y (11) (1+)
1371.298	385.4	y (11) (1+)-H2O
1371.825	465.9	y (11) (1+)-NH3
1504.286	236.7	y (12) (1+)
1485.705	280.4	y (12) (1+)-H2O
1487.353	291.1	y (12) (1+)-NH3
752.4026	697.8	y (12) (2+)
1590.036	211.9	y (13) (1+)
1573.264	367.8	y (13) (1+)-H2O, y (13) (1+)-NH3
1704.386	187.7	y (14) (1+)
843.9769	206.4	y (14) (2+)-NH3, y (14) (2+)-H2O
1863.24	201.9	y (15) (1+)
1847.084	230.7	y (15) (1+)-NH3
1978.414	192.8	y (16) (1+)
989.5201	196.7	y (16) (2+), a (24) (3+)-NH3, a (24) (3+)-H2O

1054.184	221.3	y (17) (2+)
1045.665	357	y (17) (2+)-H2O, y (17) (2+)-NH3
703.3707	221.3	y (17) (3+)
1139.858	551.2	y (18) (2+)
1129.978	645.5	y (18) (2+)-H2O
753.4069	346.1	y (18) (3+)-H2O
1220.038	562.3	y (19) (2+)
1210.297	480.7	y (19) (2+)-H2O, y (10) (1+)-H2O
1211.466	667.6	y (19) (2+)-NH3, y (10) (1+)-NH3
1267.526	578.5	y (20) (2+)-H2O, y (20) (2+)-NH3
1340.266	448.2	y (21) (2+)
1330.361	348.3	y (21) (2+)-H2O
1330.905	332.7	y (21) (2+)-NH3
1414.013	423.4	y (22) (2+)
1405.157	320.4	y (22) (2+)-H2O, y (22) (2+)-NH3
936.7171	207.5	y (22) (3+)-H2O, y (22) (3+)-NH3
1471.429	230.2	y (23) (2+)
1462.291	238.1	y (23) (2+)-H2O
1462.973	289.9	y (23) (2+)-NH3
981.371	168.9	y (23) (3+), y (16) (2+)-NH3, y (16) (2+)-H2O
974.411	167.8	y (23) (3+)-H2O
1529.045	253.8	y (24) (2+)
1520.117	289.9	y (24) (2+)-H2O, y (24) (2+)-NH3
1019.199	183.3	y (24) (3+)
1013.749	182.7	y (24) (3+)-H2O, y (24) (3+)-NH3
1572.78	267	y (25) (2+)
1563.534	227	y (25) (2+)-H2O
1564.02	270.6	y (25) (2+)-NH3, b (25) (2+)
1047.872	354.7	y (25) (3+)
1042.591	308.7	y (25) (3+)-H2O
1009.969	307.2	y (8) (1+)
993.3461	223	y (8) (1+)-NH3
1081.581	250.5	y (9) (1+)
1063.58	390.4	y (9) (1+)-H2O
1064.425	327.5	y (9) (1+)-NH3

FAGGSRDTCAKLSGCK(ac)IVDGNCK(ac)PPYVHHTLHPEAGK

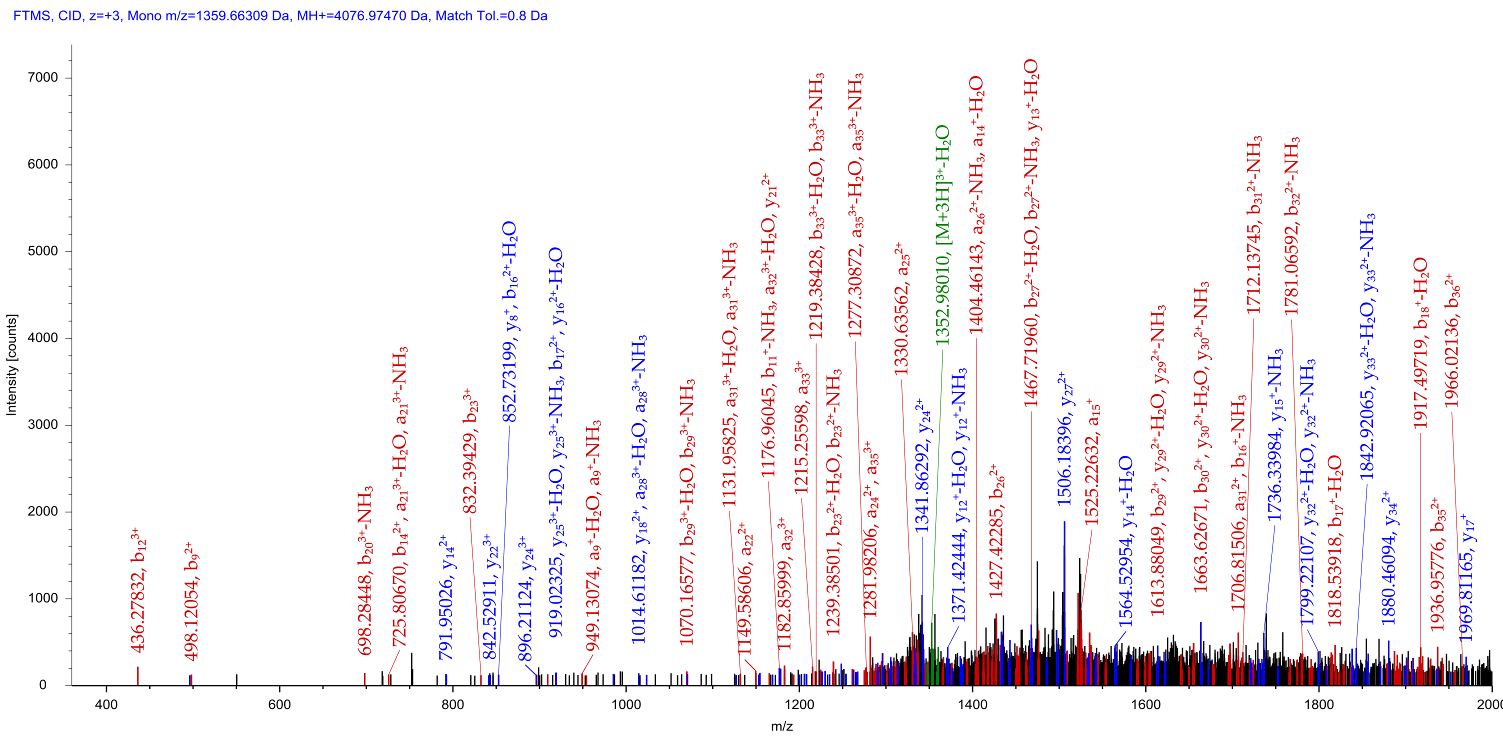

M/Z	Intensity	Matches
1306.292	284.3	b (12) (1+)
1288.545	250.4	b (12) (1+)-H2O, b (24) (2+)-NH3
1289.085	196.6	b (12) (1+)-NH3
436.2783	216.8	b (12) (3+)
1393.907	306.9	b (13) (1+)
1376.172	262.2	b (13) (1+)-NH3, b (13) (1+)-H2O
1451.251	453.2	b (14) (1+)
1554.203	347.4	b (15) (1+)
1536.763	468.1	b (15) (1+)-NH3, b (28) (2+)-NH3
1723.96	293.3	b (16) (1+)
1706.52	294	b (16) (1+)-H2O
1706.815	610.2	b (16) (1+)-NH3, a (31) (2+)
1836.665	213.1	b (17) (1+), b (33) (2+)
1818.539	468.2	b (17) (1+)-H2O
1819.861	225.1	b (17) (1+)-NH3
909.5109	130.2	b (17) (2+)-H2O
1917.497	441.6	b (18) (1+)-H2O
1919.648	337.7	b (18) (1+)-NH3
698.2845	144.9	b (20) (3+)-NH3

1239.385	278	b (23) (2+)-H2O, b (23) (2+)-NH3
832.3943	119.8	b (23) (3+)
1296.084	372.5	b (24) (2+), a (36) (3+)-NH3, a (36) (3+)-H2O, y (36) (3+)
1287.626	203.4	b (24) (2+)-H2O
1345.642	257.4	b (25) (2+)
1336.742	531.7	b (25) (2+)-NH3, b (25) (2+)-H2O
896.6827	125.9	b (25) (3+), a (17) (2+)-H2O, a (17) (2+)-NH3
1427.423	830.1	b (26) (2+)
1417.669	455.4	b (26) (2+)-NH3, b (26) (2+)-H2O
1476.334	636	b (27) (2+)
1467.72	703.4	b (27) (2+)-NH3, b (27) (2+)-H2O, y (13) (1+)-H2O
1544.346	362.9	b (28) (2+)
1535.109	612	b (28) (2+)-H2O, b (15) (1+)-H2O
1603.667	372.9	b (29) (2+)-H2O
1605.443	397.9	b (29) (2+)-NH3
1070.166	163.5	b (29) (3+)-H2O, b (29) (3+)-NH3
1663.627	731.6	b (30) (2+), y (30) (2+)-NH3, y (30) (2+)-H2O
1655.183	488.6	b (30) (2+)-H2O, b (30) (2+)-NH3
1720.194	295.4	b (31) (2+)
1710.924	324	b (31) (2+)-H2O
1712.137	384.9	b (31) (2+)-NH3
1788.972	205.5	b (32) (2+)
1779.445	365.9	b (32) (2+)-H2O
1781.066	369.9	b (32) (2+)-NH3
1193.588	124.8	b (32) (3+), b (11) (1+)
1827.891	246.6	b (33) (2+)-H2O
1829.115	241.7	b (33) (2+)-NH3
1219.384	163	b (33) (3+)-NH3, b (33) (3+)-H2O
1902.59	292.2	b (34) (2+)
1893.111	263.9	b (34) (2+)-NH3, b (34) (2+)-H2O
1262.676	184.9	b (34) (3+)-H2O, b (34) (3+)-NH3, y (22) (2+)
1936.958	446.5	b (35) (2+)
1929.016	309.7	b (35) (2+)-H2O, b (35) (2+)-NH3
1291.843	214.6	b (35) (3+)
1286.094	174.1	b (35) (3+)-H2O, b (35) (3+)-NH3
1966.021	223.2	b (36) (2+)
1957.051	180	b (36) (2+)-NH3, b (36) (2+)-H2O
1311.629	280.6	b (36) (3+)
498.1205	127.1	b (9) (2+)
1127.132	121.2	y (10) (1+)
1226.383	163.1	y (11) (1+)

1208.268	132.3	y (11) (1+)-NH3, y (11) (1+)-H2O
1388.988	251.3	y (12) (1+)
1371.424	441.9	y (12) (1+)-NH3, y (12) (1+)-H2O
1485.709	330.7	y (13) (1+)
1468.047	548.1	y (13) (1+)-NH3
496.1839	118.2	y (13) (3+)
1583.373	351.1	y (14) (1+)
1564.53	461	y (14) (1+)-H2O
1565.64	389.6	y (14) (1+)-NH3
791.9503	133.4	y (14) (2+)
1752.677	226.4	y (15) (1+)
1734.132	307.7	y (15) (1+)-H2O
1736.34	606.4	y (15) (1+)-NH3
1856.701	368.2	y (16) (1+)
1838.202	424	y (16) (1+)-NH3, y (16) (1+)-H2O
919.0233	149.6	y (16) (2+)-H2O, y (25) (3+)-NH3, b (17) (2+), y (25) (3+)-H2O
1969.812	332.2	y (17) (1+)
985.3441	135	y (17) (2+)
1070.776	131.6	y (19) (2+)
1168.984	126.6	y (21) (2+)-NH3, y (21) (2+)-H2O
1253.835	135.2	y (22) (2+)-H2O, a (34) (3+)-NH3, y (34) (3+), y (22) (2+)-NH3
842.5291	141.4	y (22) (3+)
1314.461	283.2	y (23) (2+)
1305.627	325.2	y (23) (2+)-NH3, b (36) (3+)-NH3, y (23) (2+)-H2O, b (36) (3+)-H2O
1341.863	737.2	y (24) (2+)
1333.189	429.2	y (24) (2+)-H2O
1333.968	386	y (24) (2+)-NH3
896.2112	118.3	y (24) (3+)
1385.719	339.8	y (25) (2+)
1376.377	205.9	y (25) (2+)-H2O
1377.01	197.9	y (25) (2+)-NH3
1443.187	525.7	y (26) (2+)
1433.426	619.8	y (26) (2+)-NH3, b (14) (1+)-H2O, y (26) (2+)-H2O, b (14) (1+)-NH3
1506.184	1893.2	y (27) (2+)
1496.959	641	y (27) (2+)-H2O
1498.851	410	y (27) (2+)-NH3
1541.855	374.2	y (28) (2+)
1532.9	325	y (28) (2+)-H2O
1534.506	376.6	y (28) (2+)-NH3
1023.633	123.6	y (28) (3+)-NH3, b (28) (3+)-H2O
1623.052	367.2	y (29) (2+)

1613.88	461.4	y (29) (2+)-NH3, y (29) (2+)-H2O, b (29) (2+)
1672.31	313.4	y (30) (2+)
1730.62	308.8	y (31) (2+)
1721.255	315.3	y (31) (2+)-H2O, y (31) (2+)-NH3
1154.455	146.2	y (31) (3+), b (22) (2+)-NH3, b (22) (2+)-H2O
1799.221	393.2	y (32) (2+)-NH3, y (32) (2+)-H2O
1205.861	135.9	y (32) (3+)
1200.142	126.8	y (32) (3+)-NH3, y (32) (3+)-H2O
1852.036	168.3	y (33) (2+)
1842.921	432.1	y (33) (2+)-NH3, y (33) (2+)-H2O
1234.886	129.9	y (33) (3+)
1229.699	168.7	y (33) (3+)-H2O, y (33) (3+)-NH3
1880.461	518.5	y (34) (2+)
1870.881	277.2	y (34) (2+)-H2O
1871.374	224.1	y (34) (2+)-NH3
1248.299	251.7	y (34) (3+)-NH3, b (23) (2+), y (34) (3+)-H2O
1899.213	232.7	y (35) (2+)-H2O
1901.074	323.1	y (35) (2+)-NH3
1267.233	161.7	y (35) (3+)-NH3, y (35) (3+)-H2O
1936.228	194	y (36) (2+)-NH3, y (36) (2+)-H2O, b (18) (1+)
1290.59	262.5	y (36) (3+)-NH3, y (36) (3+)-H2O
852.732	126	y (8) (1+), b (16) (2+)-H2O

HDLYEQTNRSPTPK(ac)TEEEQIAK

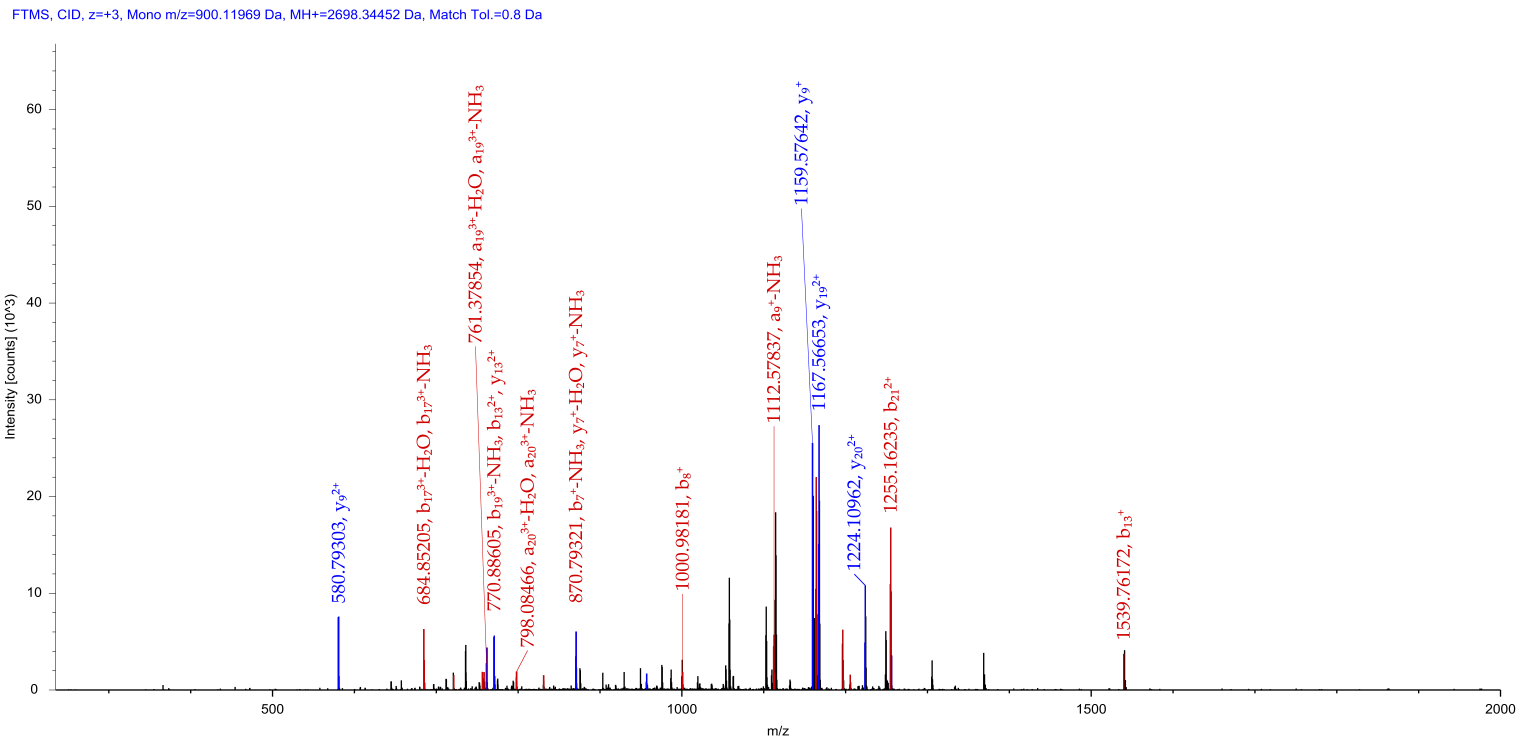

M/Z	Intensity	Matches
721.3515	1534.5	b (12) (2+)
1539.762	3730.4	b (13) (1+)
761.8802	4388.3	b (13) (2+)-H2O, b (13) (2+)-NH3, y (13) (2+)-H2O
684.8521	6289.6	b (17) (3+)-NH3, b (17) (3+)-H2O
1164.07	22016.4	b (19) (2+)
770.384	5463	b (19) (3+)-H2O
1255.162	16785.8	b (21) (2+)
831.107	1514.7	b (21) (3+)-NH3, b (21) (3+)-H2O
870.7932	6047.2	b (7) (1+)-NH3, y (7) (1+)-H2O, y (7) (1+)-NH3
1000.982	1845.3	b (8) (1+)
1256.164	3571.9	y (10) (1+)
770.8861	5613.3	y (13) (2+), b (13) (2+), b (19) (3+)-NH3
1167.567	27374.6	y (19) (2+)
1224.11	10804.2	y (20) (2+)
1159.576	25529.1	y (9) (1+)
580.793	7590.2	y (9) (2+)

ILMATAISK(ac)MFLSEPMKSSLGEDGAVEPLVEMFK(ac)SGNLEAK(ac)

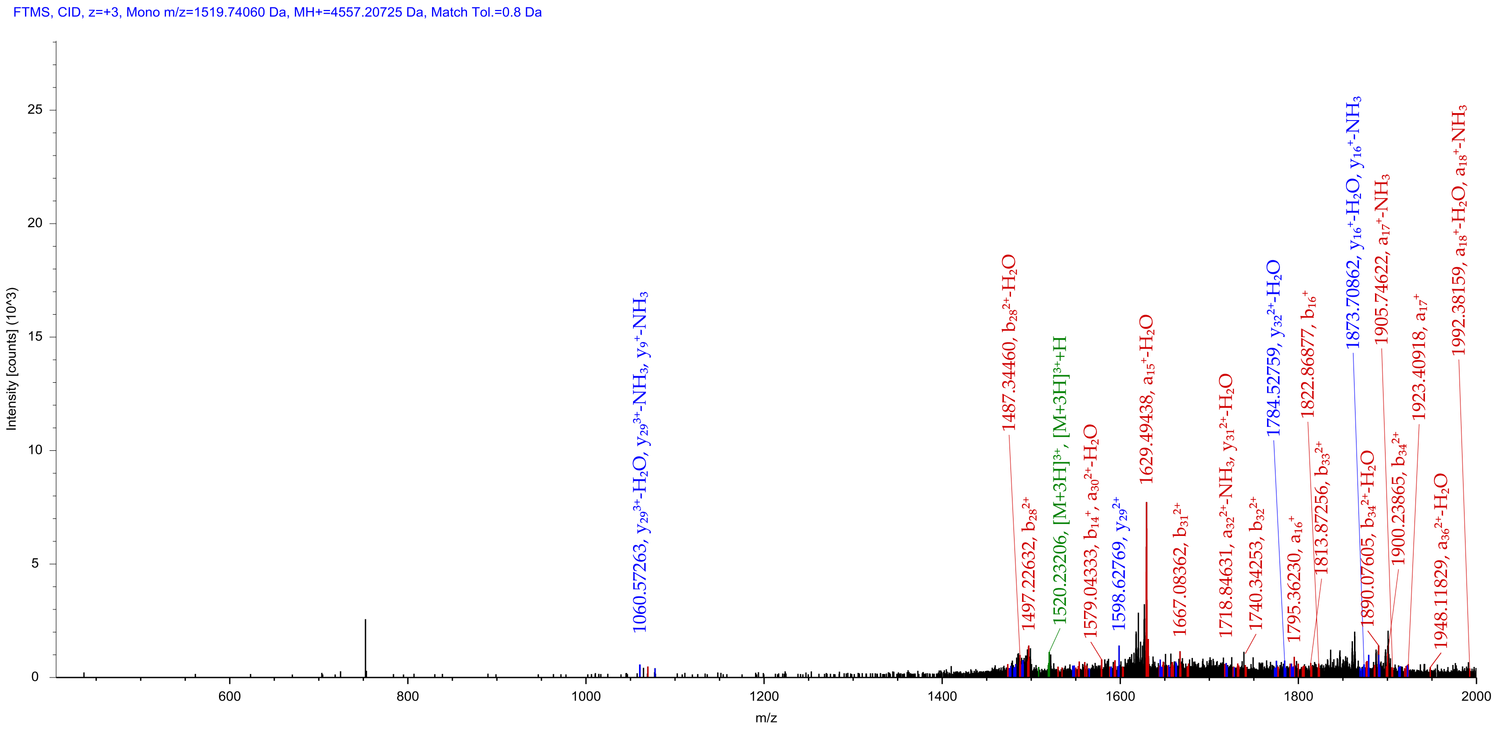

M/Z	Intensity	Matches
1560.164	678	b (14) (1+)-H2O
1562.031	531.3	b (14) (1+)-NH3
1676.646	613.9	b (15) (1+)
1822.869	610.1	b (16) (1+)
1497.226	1408.9	b (28) (2+)
1487.345	990.8	b (28) (2+)-H2O
1487.722	725.9	b (28) (2+)-NH3
1553.89	707.4	b (29) (2+)
1602.915	482.8	b (30) (2+)
1594.052	755.1	b (30) (2+)-NH3, b (30) (2+)-H2O
1069.546	483.1	b (30) (3+)
1667.084	1155.8	b (31) (2+)
1658.509	677	b (31) (2+)-H2O, b (15) (1+)-H2O, b (31) (2+)-NH3, b (15) (1+)-NH3
1740.343	552.5	b (32) (2+)
1731.844	613.3	b (32) (2+)-H2O, b (32) (2+)-NH3
1813.873	642.4	b (33) (2+)
1805.697	499.5	b (33) (2+)-H2O, b (16) (1+)-H2O
1806.357	519	b (33) (2+)-NH3, b (16) (1+)-NH3
1900.239	1058.1	b (34) (2+)

1890.076	1410.4	b (34) (2+)-H2O
1565.179	397.8	y (13) (1+)
1548.174	527.6	y (13) (1+)-H2O, y (13) (1+)-NH3
1662.225	697.8	y (14) (1+)
1644.797	790.3	y (14) (1+)-H2O, a (31) (2+)-H2O, a (31) (2+)-NH3
1792.183	610.5	y (15) (1+), a (33) (2+)-H2O, a (33) (2+)-NH3
1773.852	436.7	y (15) (1+)-H2O
1775.348	735.6	y (15) (1+)-NH3
1890.238	1002.6	y (16) (1+), b (34) (2+)-NH3
1873.709	579.2	y (16) (1+)-NH3, y (16) (1+)-H2O
1490.718	772.2	y (27) (2+)
1481.096	451.9	y (27) (2+)-NH3, y (27) (2+)-H2O
1546.604	519.7	y (28) (2+)-NH3
1598.628	1406.4	y (29) (2+)
1589.752	480	y (29) (2+)-H2O, y (29) (2+)-NH3
1654.53	552.6	y (30) (2+)
1645.777	472.3	y (30) (2+)-NH3, y (14) (1+)-NH3, y (30) (2+)-H2O
1728.084	448.6	y (31) (2+)
1718.846	640.9	y (31) (2+)-H2O, a (32) (2+)-NH3
1719.817	498.5	y (31) (2+)-NH3
1794.521	490.1	y (32) (2+)
1784.528	746.6	y (32) (2+)-H2O
1785.865	414.5	y (32) (2+)-NH3
1878.895	997.9	y (33) (2+)
1869.233	442.4	y (33) (2+)-H2O
1871.092	388.5	y (33) (2+)-NH3
1922.427	539.2	y (34) (2+)
1912.9	463.1	y (34) (2+)-H2O
1914.308	497.5	y (34) (2+)-NH3
1475.713	415.4	y (40) (3+)-H2O
1477.094	514.8	y (40) (3+)-NH3
1077.544	408	y (9) (1+)
1060.573	574	y (9) (1+)-NH3, y (29) (3+)-NH3, y (29) (3+)-H2O

ISPTRDVYCPIQK(ac)TKNHDLSSCKVFLSAMK

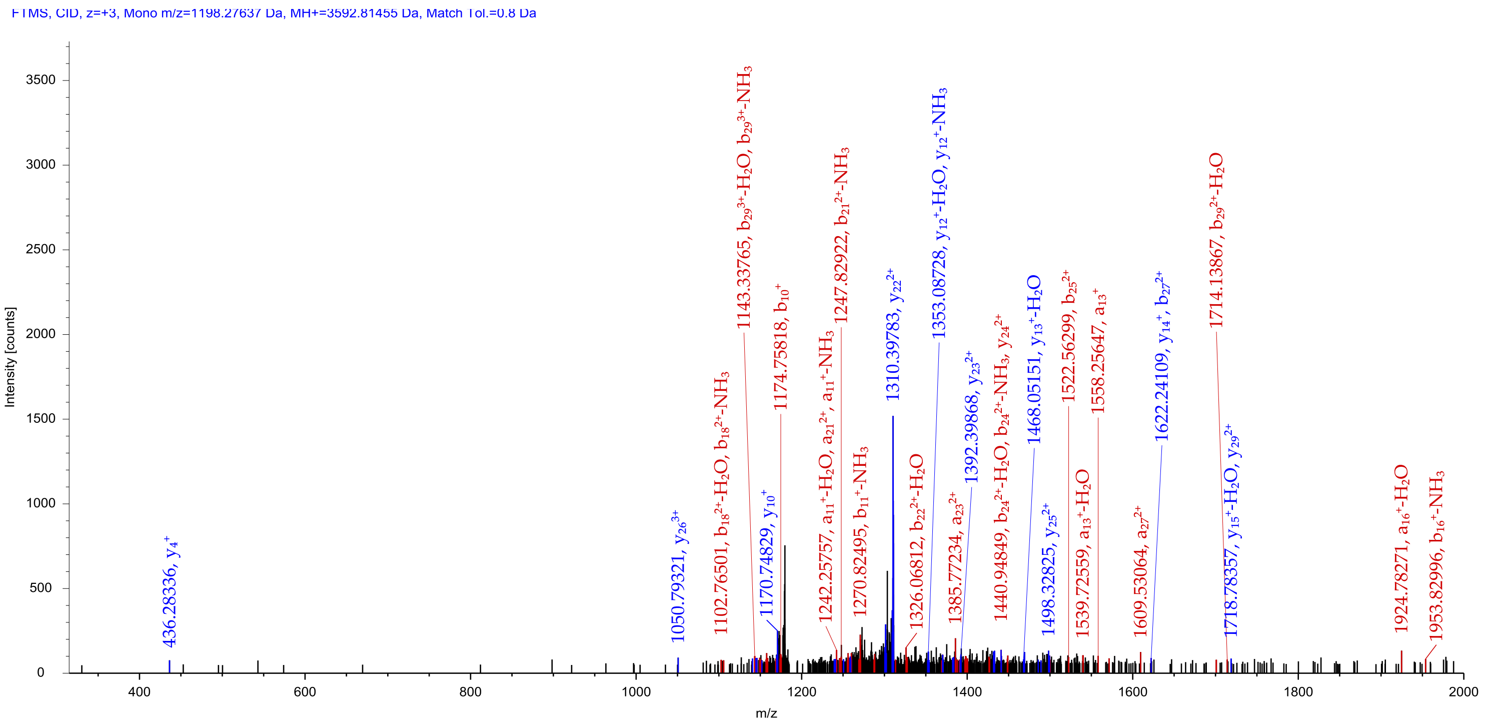

M/Z	Intensity	Matches
1174.758	113.2	b (10) (1+)
1157.034	80.6	b (10) (1+)-H2O
1157.745	118.6	b (10) (1+)-NH3
1288.271	113.9	b (11) (1+)
1269.595	86	b (11) (1+)-H2O
1270.825	227.5	b (11) (1+)-NH3
1398.027	101.5	b (12) (1+)-H2O, b (12) (1+)-NH3
1953.83	84.3	b (16) (1+)-NH3
1102.765	78.6	b (18) (2+)-NH3, b (18) (2+)-H2O
1168.745	87	b (19) (2+)
1158.953	90.1	b (19) (2+)-H2O
1256.095	118.5	b (21) (2+)
1246.166	89.6	b (21) (2+)-H2O
1247.829	98.2	b (21) (2+)-NH3
1326.068	151.6	b (22) (2+)-H2O
1327.245	95.4	b (22) (2+)-NH3
1400.414	87.5	b (23) (2+)
1390.895	80.4	b (23) (2+)-NH3, b (23) (2+)-H2O
1449.062	104.4	b (24) (2+)

1522.563	95.7	b (25) (2+)
1571.393	87.7	b (26) (2+)-NH3
1105.8	76.2	b (28) (3+)
1714.139	80.8	b (29) (2+)-H2O
1149.34	91.4	b (29) (3+)
1143.338	100.9	b (29) (3+)-NH3, b (29) (3+)-H2O
1170.748	250.7	y (10) (1+)
1151.967	76.2	y (10) (1+)-H2O
1258.381	87.9	y (11) (1+)
1239.383	82.6	y (11) (1+)-H2O
1240.92	84	y (11) (1+)-NH3
1353.087	127.2	y (12) (1+)-NH3, y (12) (1+)-H2O
1485.827	79.9	y (13) (1+)
1468.052	81.5	y (13) (1+)-H2O
1469.254	125.4	y (13) (1+)-NH3
1622.241	89.6	y (14) (1+), b (27) (2+)
1144.754	89.9	y (19) (2+)-NH3, y (19) (2+)-H2O
1258.808	98.7	y (21) (2+)
1250.592	82.7	y (21) (2+)-NH3
1310.398	1519.4	y (22) (2+)
1301.508	288.3	y (22) (2+)-NH3, y (22) (2+)-H2O
1392.399	111	y (23) (2+)
1383.023	92.4	y (23) (2+)-H2O
1383.769	95.1	y (23) (2+)-NH3
1440.948	137.8	y (24) (2+), b (24) (2+)-NH3, b (24) (2+)-H2O
1432.933	132.4	y (24) (2+)-H2O, y (24) (2+)-NH3
1498.328	133	y (25) (2+)
1489.086	76	y (25) (2+)-H2O
1050.793	92	y (26) (3+)
1718.784	86.2	y (29) (2+), y (15) (1+)-H2O
1140.553	86.8	y (29) (3+)-H2O, y (29) (3+)-NH3, a (29) (3+)
436.2834	76.2	y (4) (1+)

K(ac)HAVQECVLKVDGGCSCTCMK(ac)PK

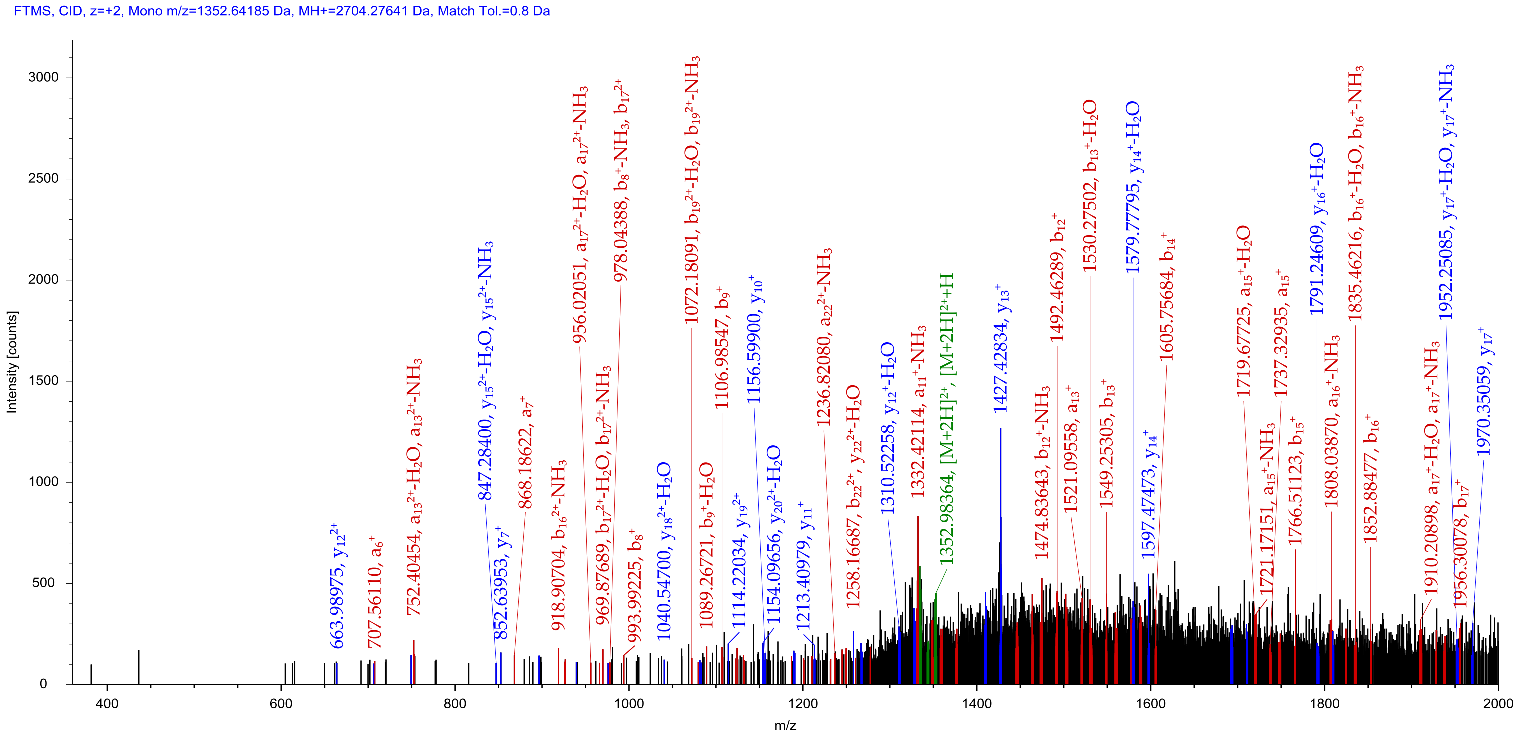

M/Z	Intensity	Matches
1277.596	187.6	b (10) (1+)
1260.605	132.7	b (10) (1+)-NH3
1377.081	276.4	b (11) (1+)
1358.151	276.3	b (11) (1+)-H2O
1360.089	276.3	b (11) (1+)-NH3
1492.463	463.1	b (12) (1+)
1473.021	310.3	b (12) (1+)-H2O
1474.836	527.8	b (12) (1+)-NH3
1549.253	450.5	b (13) (1+)
1530.275	421	b (13) (1+)-H2O
1531.903	280.9	b (13) (1+)-NH3
1605.757	367.7	b (14) (1+)
1588.043	390.5	b (14) (1+)-H2O, b (14) (1+)-NH3
1766.511	264.9	b (15) (1+)
1747.389	211.3	b (15) (1+)-H2O
1749.058	252.8	b (15) (1+)-NH3
1852.885	276	b (16) (1+)
1835.462	217	b (16) (1+)-NH3, b (16) (1+)-H2O
926.7204	124.5	b (16) (2+)
918.907	180.6	b (16) (2+)-NH3
1956.301	303.5	b (17) (1+)
1938.623	240.2	b (17) (1+)-H2O, b (17) (1+)-NH3
978.0439	109.2	b (17) (2+), b (8) (1+)-NH3
969.8769	174.2	b (17) (2+)-NH3, b (17) (2+)-H2O
1072.181	131.1	b (19) (2+)-NH3, b (19) (2+)-H2O
1231.594	118	b (21) (2+)
1222.596	169.9	b (21) (2+)-H2O, b (21) (2+)-NH3
1279.003	202	b (22) (2+)
1270.727	162.5	b (22) (2+)-H2O, b (22) (2+)-NH3

993.9923	144.6	b (8) (1+)
975.984	106.9	b (8) (1+)-H2O, y (17) (2+)-H2O
1106.985	186	b (9) (1+)
1089.267	188.5	b (9) (1+)-H2O
1156.599	157.4	y (10) (1+)
1213.41	194.1	y (11) (1+)
1327.76	379.7	y (12) (1+)
1310.523	287.4	y (12) (1+)-H2O
1312.245	259.9	y (12) (1+)-NH3
663.9898	107.6	y (12) (2+)
1427.428	1268.7	y (13) (1+)
1409.988	457.4	y (13) (1+)-H2O, y (13) (1+)-NH3
706.3967	104.1	y (13) (2+)-NH3
1597.475	548.3	y (14) (1+)
1579.778	412.7	y (14) (1+)-H2O
1580.897	384.6	y (14) (1+)-NH3
1710.105	299.3	y (15) (1+)
1692.261	276.6	y (15) (1+)-H2O
1693.611	293	y (15) (1+)-NH3
847.284	106.3	y (15) (2+)-NH3, y (15) (2+)-H2O
1809.456	266.2	y (16) (1+)
1791.246	207	y (16) (1+)-H2O
1793.142	188.9	y (16) (1+)-NH3
896.625	143.2	y (16) (2+)-NH3, y (16) (2+)-H2O
1970.351	303.1	y (17) (1+)
1952.251	223	y (17) (1+)-H2O, y (17) (1+)-NH3
1040.547	123.4	y (18) (2+)-H2O
1114.22	202.4	y (19) (2+)
1154.097	206.9	y (20) (2+)-H2O
1189.66	167.7	y (21) (2+)-H2O
1190.677	157	y (21) (2+)-NH3
1266.85	205.9	y (22) (2+)
1258.167	265.1	y (22) (2+)-H2O
749.3847	145	y (6) (1+)
852.6395	158.7	y (7) (1+)
939.4558	111.5	y (8) (1+)
1081.552	122	y (9) (1+)-H2O
1082.907	109	y (9) (1+)-NH3

KHGAKPFACRRCAKPFAVK(ac)GDWR

FTMS, CID, z=+3, Mono m/z=924.14447 Da, MH+=2770.41886 Da, Match Tol.=0.8 Da

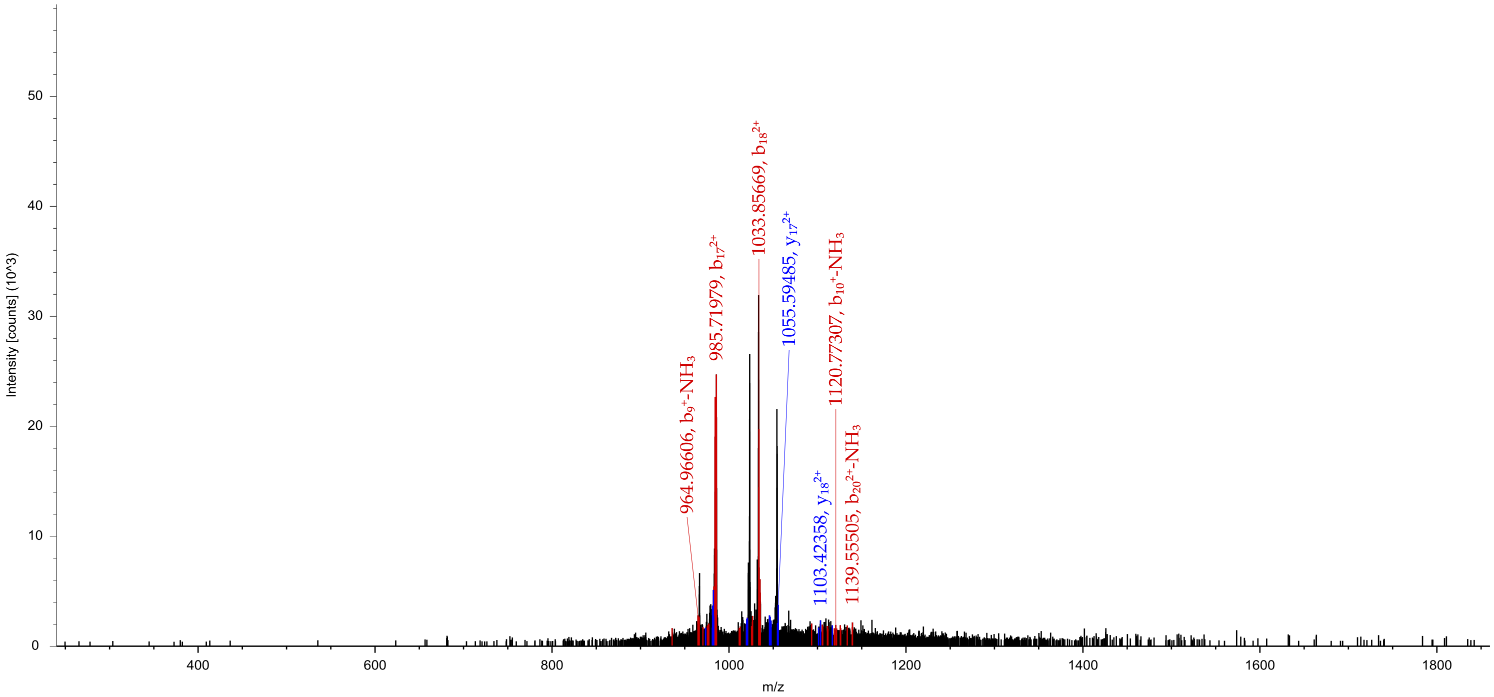

M/Z	Intensity	Matches
1139.35	1721.3	b (10) (1+)
1120.773	1937.4	b (10) (1+)-NH3
985.7198	24707.5	b (17) (2+)
977.1314	2061.2	b (17) (2+)-NH3
1033.857	19759.3	b (18) (2+)
1026.668	2755.8	b (18) (2+)-NH3
1119.107	1625	b (19) (2+)
1111.335	1842.2	b (19) (2+)-NH3, a (10) (1+)
1139.555	2160.7	b (20) (2+)-NH3
982.9666	5431.7	b (9) (1+)
964.9661	2806.5	b (9) (1+)-NH3
982.4705	5122.7	y (16) (2+)
973.5435	1671.3	y (16) (2+)-H2O, y (16) (2+)-NH3
1055.595	3762.8	y (17) (2+)
1045.77	2805.6	y (17) (2+)-H2O
1046.397	2671.1	y (17) (2+)-NH3
1103.424	2355.2	y (18) (2+)
1020.584	2585.4	y (8) (1+), a (18) (2+)
1116.78	1899.8	y (9) (1+)
1101.227	1693.2	y (9) (1+)-NH3

KPAAK(ac)KPAEEEPAAEKAEK(ac)APAGK(ac)KPK

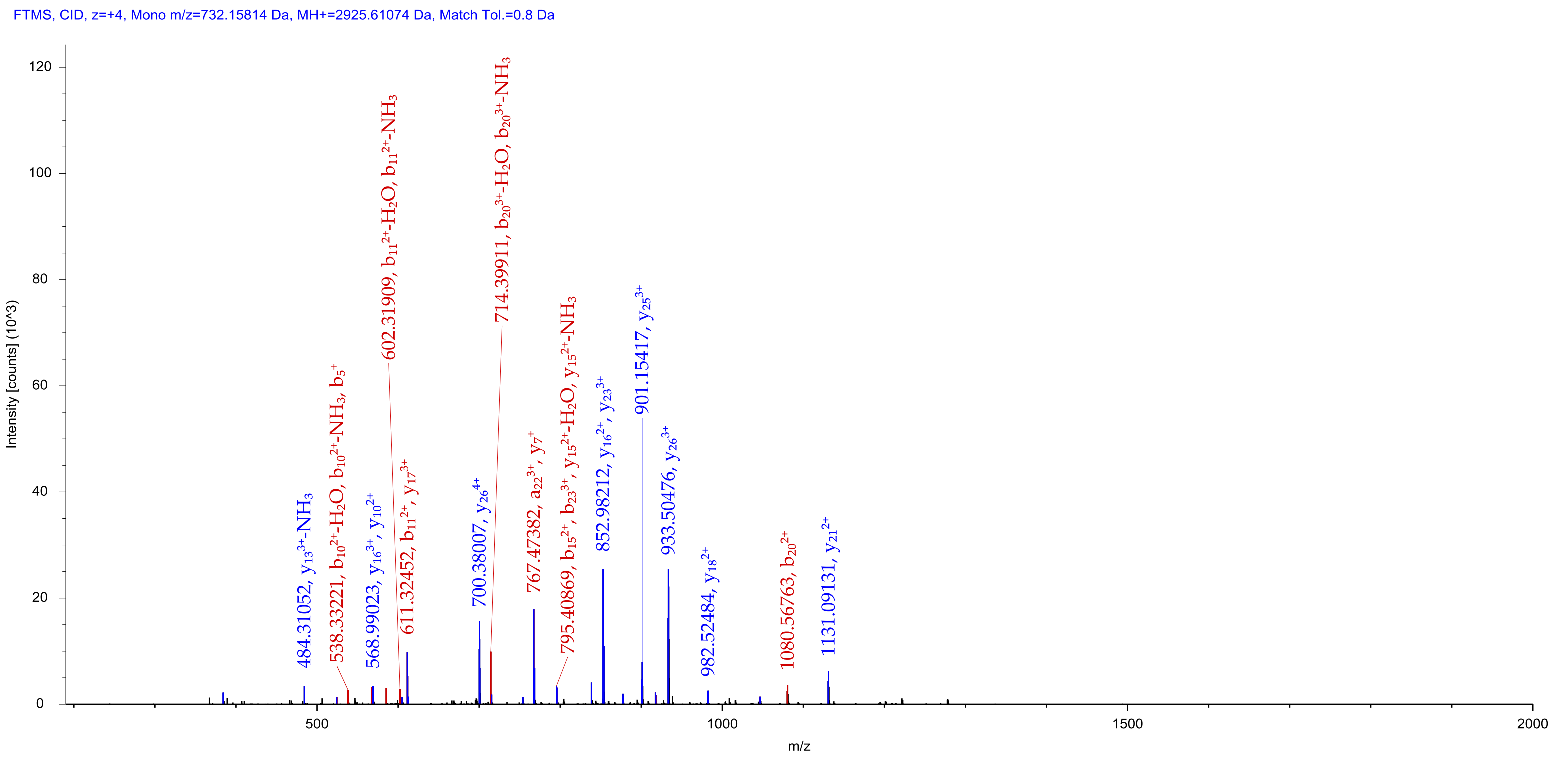

M/Z	Intensity	Matches
730.8873	5826.9	b (14) (2+)
721.3825	15469.3	b (14) (2+)-H2O
640.3351	5847.1	b (18) (3+), y (23) (4+)
384.2412	37751.2	b (18) (5+), y (7) (2+), y (14) (4+)
697.0369	17565.5	b (19) (3+)
720.7142	51386.3	b (20) (3+)
714.7103	8194.1	b (20) (3+)-H2O, b (20) (3+)-NH3
795.4097	14938.8	b (23) (3+), y (15) (2+)-NH3, y (15) (2+)-H2O, b (15) (2+)
499.2734	4571.1	b (24) (5+)-NH3, b (24) (5+)-H2O
568.9904	8677.1	y (10) (2+), y (16) (3+)
604.8513	5986.4	y (11) (2+)
768.478	11336.3	y (14) (2+)
852.9831	6890.1	y (16) (2+), y (23) (3+)
611.3253	21994	y (17) (3+), b (11) (2+)
605.353	3530.5	y (17) (3+)-H2O
697.3711	11049.5	y (19) (3+)
721.0482	31011.3	y (20) (3+)
715.0442	3387	y (20) (3+)-H2O
754.397	6085.8	y (21) (3+)

565.7983	4508.1	y (21) (4+)
560.5068	16614.2	y (21) (4+)-H2O, y (26) (5+), b (21) (4+)-H2O, y (10) (2+)-H2O, y (10) (2+)-NH3, b (21) (4+)-NH3
560.9072	7094.4	y (21) (4+)-NH3
796.4128	3386.2	y (22) (3+)
676.1182	6442	y (25) (4+)
700.381	67304.3	y (26) (4+)
695.3707	8633.4	y (26) (4+)-H2O, b (13) (2+), y (26) (4+)-NH3
599.3857	26230.9	y (5) (1+)
670.4226	7790.8	y (6) (1+)
767.4749	32629.6	y (7) (1+), a (22) (3+)
375.236	4810.7	y (7) (2+)-NH3, a (18) (5+)-NH3, a (18) (5+)-H2O

K(ac)QAIELLR

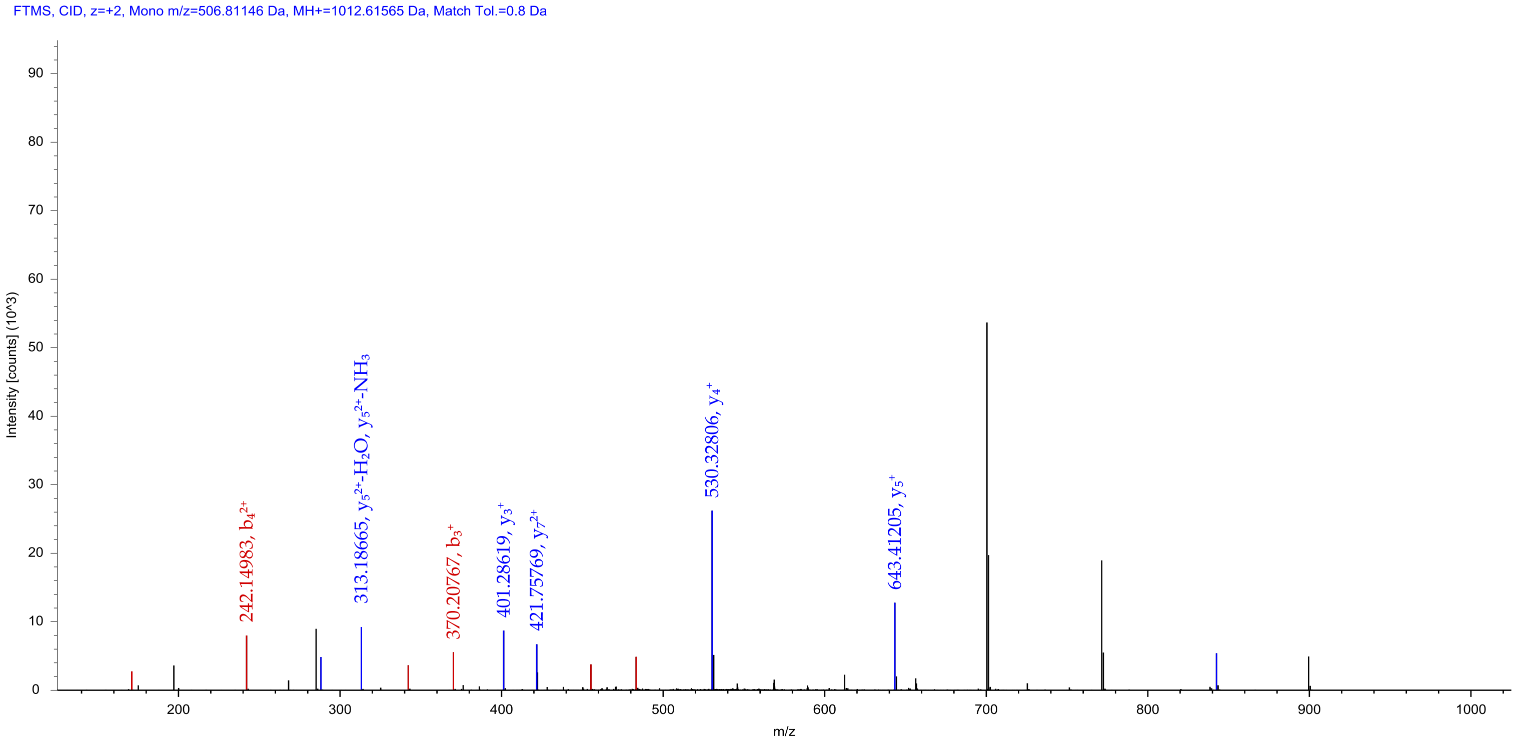

M/Z	Intensity	Matches
171.1126	2745.1	b (1) (1+), a (3) (2+)
370.2077	5549.9	b (3) (1+)
483.2911	4875.9	b (4) (1+)
242.1498	7991.6	b (4) (2+)
288.2026	4840.7	y (2) (1+)
401.2862	8720.3	y (3) (1+)
530.3281	26218.9	y (4) (1+)
643.4121	12779.2	y (5) (1+)
313.1867	9230.3	y (5) (2+)-NH3, y (5) (2+)-H2O
842.5067	5396.7	y (7) (1+)
421.7577	6704.8	y (7) (2+)

K(ac)YFILFK

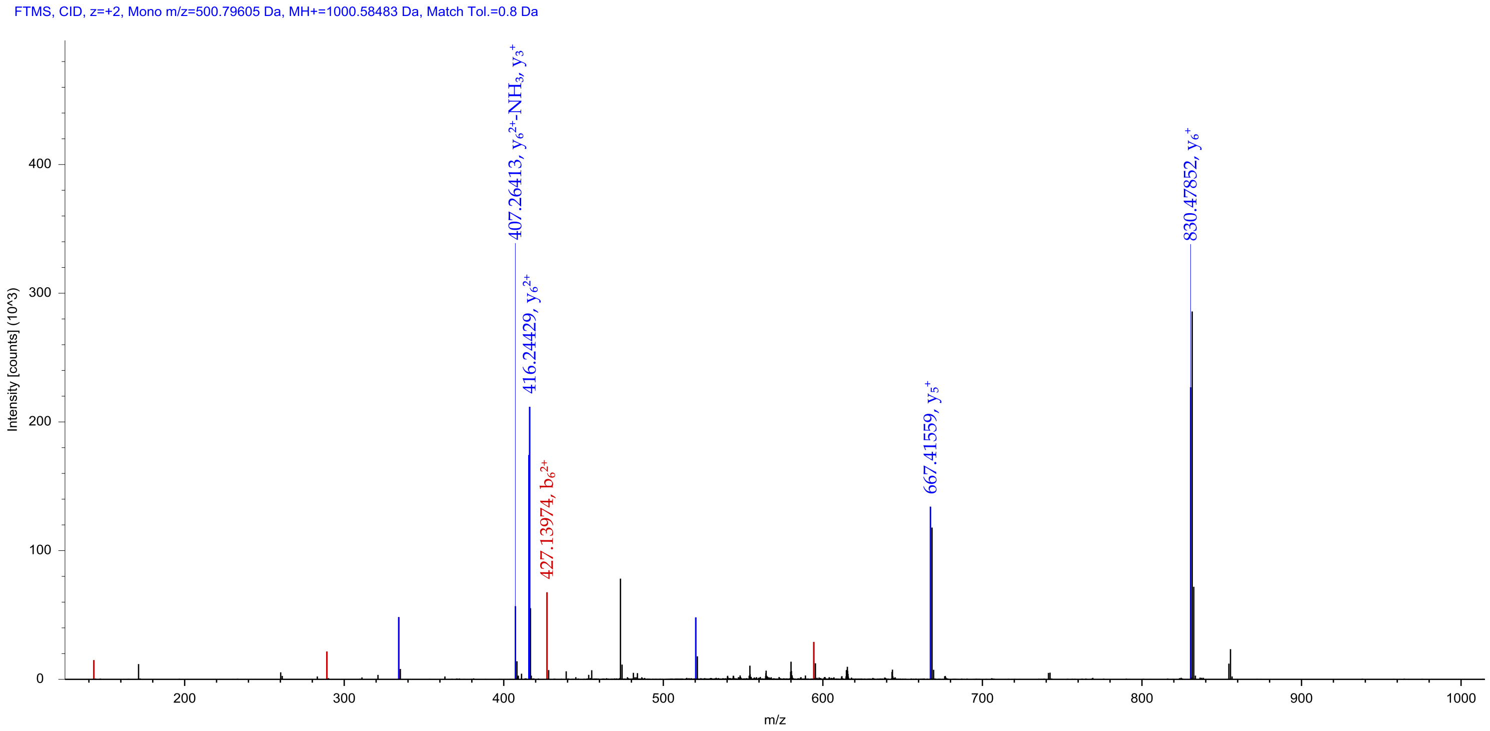

M/Z	Intensity	Matches
334.1753	48303.7	b (2) (1+), y (5) (2+)
594.3264	29045.1	b (4) (1+)
427.1397	67629.7	b (6) (2+)
520.3477	48150.8	y (4) (1+)
667.4156	134134.6	y (5) (1+)
830.4785	226915.3	y (6) (1+)
416.2443	211759.3	y (6) (2+)
407.2641	56786.6	y (6) (2+)-NH3, y (3) (1+)

LCHVNFGCMTSLANMSLIPKFTLVKGSK(ac)CHTCVQSK

FTMS, CID, z=+3, Mono m/z=1352.98914 Da, MH+=4056.95285 Da, Match Tol.=0.8 Da

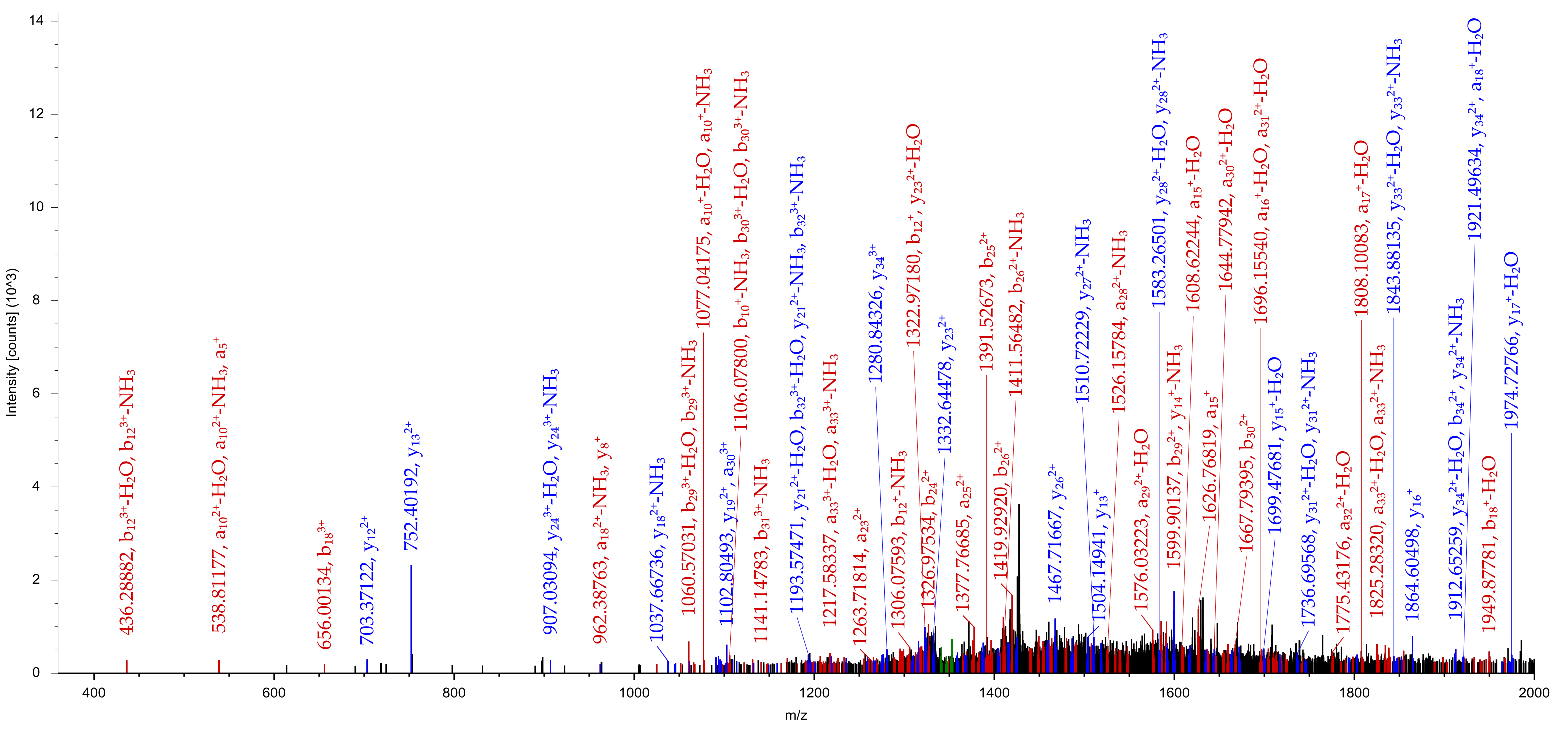

M/Z	Intensity	Matches
1122.236	231.6	b (10) (1+)
1104.979	213.8	b (10) (1+)-H2O
1106.078	381.8	b (10) (1+)-NH3, b (30) (3+)-H2O, b (30) (3+)-NH3
1210.171	250.7	b (11) (1+)
1190.76	266.9	b (11) (1+)-H2O
1193.173	276.3	b (11) (1+)-NH3
1306.076	555	b (12) (1+)-NH3
436.2888	275.9	b (12) (3+)-H2O, b (12) (3+)-NH3
1393.798	403.6	b (13) (1+)
1376.162	702.8	b (13) (1+)-NH3, b (13) (1+)-H2O
1507.965	641	b (14) (1+)
1489.219	578.3	b (14) (1+)-H2O
1490.709	661.7	b (14) (1+)-NH3
1636.801	521.8	b (15) (1+)-H2O
1637.685	472.4	b (15) (1+)-NH3
1742.488	342.7	b (16) (1+)
1723.669	407.2	b (16) (1+)-H2O
1725.403	294.4	b (16) (1+)-NH3
1854.875	485.1	b (17) (1+)

1836.733	328.4	b (17) (1+)-H2O
1838.366	549.3	b (17) (1+)-NH3
1967.34	323.8	b (18) (1+)
1949.878	467.4	b (18) (1+)-H2O
1950.484	360.2	b (18) (1+)-NH3
656.0013	201.9	b (18) (3+)
1025.109	196.7	b (19) (2+)-NH3
1170.163	326.1	b (21) (2+)
1221.777	249.5	b (22) (2+)
1212.585	250.2	b (22) (2+)-NH3, b (22) (2+)-H2O
1269.242	324.3	b (23) (2+)-H2O, b (23) (2+)-NH3, b (34) (3+)-NH3, b (34) (3+)-H2O
1326.975	1054.5	b (24) (2+)
1318.738	572.1	b (24) (2+)-H2O
1319.415	575.9	b (24) (2+)-NH3
1391.527	769.6	b (25) (2+)
1382.517	458.7	b (25) (2+)-NH3, b (25) (2+)-H2O
1419.929	1676	b (26) (2+)
1410.176	1211	b (26) (2+)-H2O
1411.565	928.7	b (26) (2+)-NH3
1463.838	748.1	b (27) (2+)
1453.98	696.7	b (27) (2+)-H2O, b (27) (2+)-NH3
1548.037	579.2	b (28) (2+)
1539.879	497.8	b (28) (2+)-H2O, b (28) (2+)-NH3
1599.901	1762.9	b (29) (2+), y (14) (1+)-NH3
1591.329	1109.3	b (29) (2+)-H2O, b (29) (2+)-NH3
1066.126	232.7	b (29) (3+)
1060.57	681	b (29) (3+)-H2O, b (29) (3+)-NH3
1667.794	628.8	b (30) (2+)
1659.793	624.1	b (30) (2+)-NH3, b (30) (2+)-H2O
1718.973	339.3	b (31) (2+)
1710.158	442	b (31) (2+)-H2O, b (31) (2+)-NH3
1141.148	238.2	b (31) (3+)-NH3
1798.358	356.7	b (32) (2+)
1790.348	331.5	b (32) (2+)-NH3, b (32) (2+)-H2O
1199.49	253.7	b (32) (3+)
1848.892	342.7	b (33) (2+)
1839.589	355.1	b (33) (2+)-NH3, b (33) (2+)-H2O
1233.337	348.9	b (33) (3+)
1226.602	325.5	b (33) (3+)-H2O, b (33) (3+)-NH3
1902.909	240.1	b (34) (2+)-H2O
1903.545	234.4	b (34) (2+)-NH3

1946.563	301.8	b (35) (2+)-H2O
1304.961	402.5	b (35) (3+), b (12) (1+)-H2O, a (24) (2+)-NH3
1297.693	472.4	b (35) (3+)-H2O
1299.338	510.2	b (35) (3+)-NH3
1219.088	361.5	y (10) (1+)
1201.169	250.5	y (10) (1+)-H2O
1259.315	358.1	y (11) (1+)-H2O, y (11) (1+)-NH3, a (34) (3+)-H2O
1404.182	544.3	y (12) (1+)
1386.495	461.2	y (12) (1+)-H2O
1387.807	657.9	y (12) (1+)-NH3
703.3712	297.5	y (12) (2+)
1504.149	779.4	y (13) (1+)
1486.4	461.6	y (13) (1+)-H2O
1486.596	747.7	y (13) (1+)-NH3
752.4019	2322.1	y (13) (2+)
1617.434	593.3	y (14) (1+)
1599.325	1351.9	y (14) (1+)-H2O
1717.308	403.6	y (15) (1+)
1699.477	530	y (15) (1+)-H2O
1700.13	340.9	y (15) (1+)-NH3
1864.605	797.4	y (16) (1+)
1847.589	318.9	y (16) (1+)-NH3, y (16) (1+)-H2O
1974.728	415	y (17) (1+)-H2O
1976.544	332.9	y (17) (1+)-NH3
1046.042	215.9	y (18) (2+)
1037.667	261	y (18) (2+)-NH3
1093.701	369.7	y (19) (2+)-H2O, y (19) (2+)-NH3, a (10) (1+)
1149.355	184.8	y (20) (2+)-H2O, y (20) (2+)-NH3
1193.575	400.9	y (21) (2+)-H2O, b (32) (3+)-H2O, b (32) (3+)-NH3, y (21) (2+)-NH3
1265.935	279.6	y (22) (2+)-H2O
1267.603	273.2	y (22) (2+)-NH3
1332.645	869.6	y (23) (2+)
1322.972	990.9	y (23) (2+)-H2O, b (12) (1+)
1324.299	764.9	y (23) (2+)-NH3
1368.425	392.1	y (24) (2+), a (25) (2+)-H2O
1359.144	447.3	y (24) (2+)-H2O, y (24) (2+)-NH3
907.0309	289.4	y (24) (3+)-H2O, y (24) (3+)-NH3
1425.432	770.4	y (25) (2+)
1415.301	665.5	y (25) (2+)-H2O
1416.672	627.2	y (25) (2+)-NH3
1467.717	1175.2	y (26) (2+)

1459.691	718.5	y (26) (2+)-NH3, y (26) (2+)-H2O
1518.621	650.9	y (27) (2+)
1509.002	602.4	y (27) (2+)-H2O
1510.722	778.2	y (27) (2+)-NH3
1592.419	640.3	y (28) (2+)
1583.265	490.8	y (28) (2+)-NH3, y (28) (2+)-H2O
1061.573	256.5	y (28) (3+)
1644.452	515.4	y (29) (2+)
1634.785	496.3	y (29) (2+)-H2O
1635.636	509.3	y (29) (2+)-NH3
1095.442	295.8	y (29) (3+)
1090.837	339.5	y (29) (3+)-NH3, y (29) (3+)-H2O
1672.129	571.3	y (30) (2+)
1662.795	407.9	y (30) (2+)-H2O
1664.122	501.6	y (30) (2+)-NH3
1109.265	299.7	y (30) (3+)-H2O, y (30) (3+)-NH3
1745.511	296.5	y (31) (2+)
1736.696	427.4	y (31) (2+)-NH3, y (31) (2+)-H2O
1159.027	223.6	y (31) (3+)-NH3, y (20) (2+), y (31) (3+)-H2O
1803.153	402.3	y (32) (2+)
1793.853	337.2	y (32) (2+)-H2O, y (32) (2+)-NH3
1195.568	277.1	y (32) (3+)-H2O
1196.229	230.4	y (32) (3+)-NH3
1852.548	359.3	y (33) (2+)
1843.881	367.1	y (33) (2+)-NH3, y (33) (2+)-H2O
1235.609	329.7	y (33) (3+)
1912.653	514.5	y (34) (2+)-NH3, y (34) (2+)-H2O, b (34) (2+)
1280.843	512.6	y (34) (3+)
1275.625	395.8	y (34) (3+)-NH3, y (22) (2+), b (34) (3+), y (34) (3+)-H2O
1971.93	262.4	y (35) (2+)
1963.839	250	y (35) (2+)-H2O
1964.45	252.7	y (35) (2+)-NH3
1315.969	689.8	y (35) (3+)
1309.421	361.6	y (35) (3+)-H2O
1310.185	393.2	y (35) (3+)-NH3
1132.757	216.7	y (9) (1+)
1113.89	257.1	y (9) (1+)-H2O

LNERFLELGAVLEPGK(ac)TPKMDK

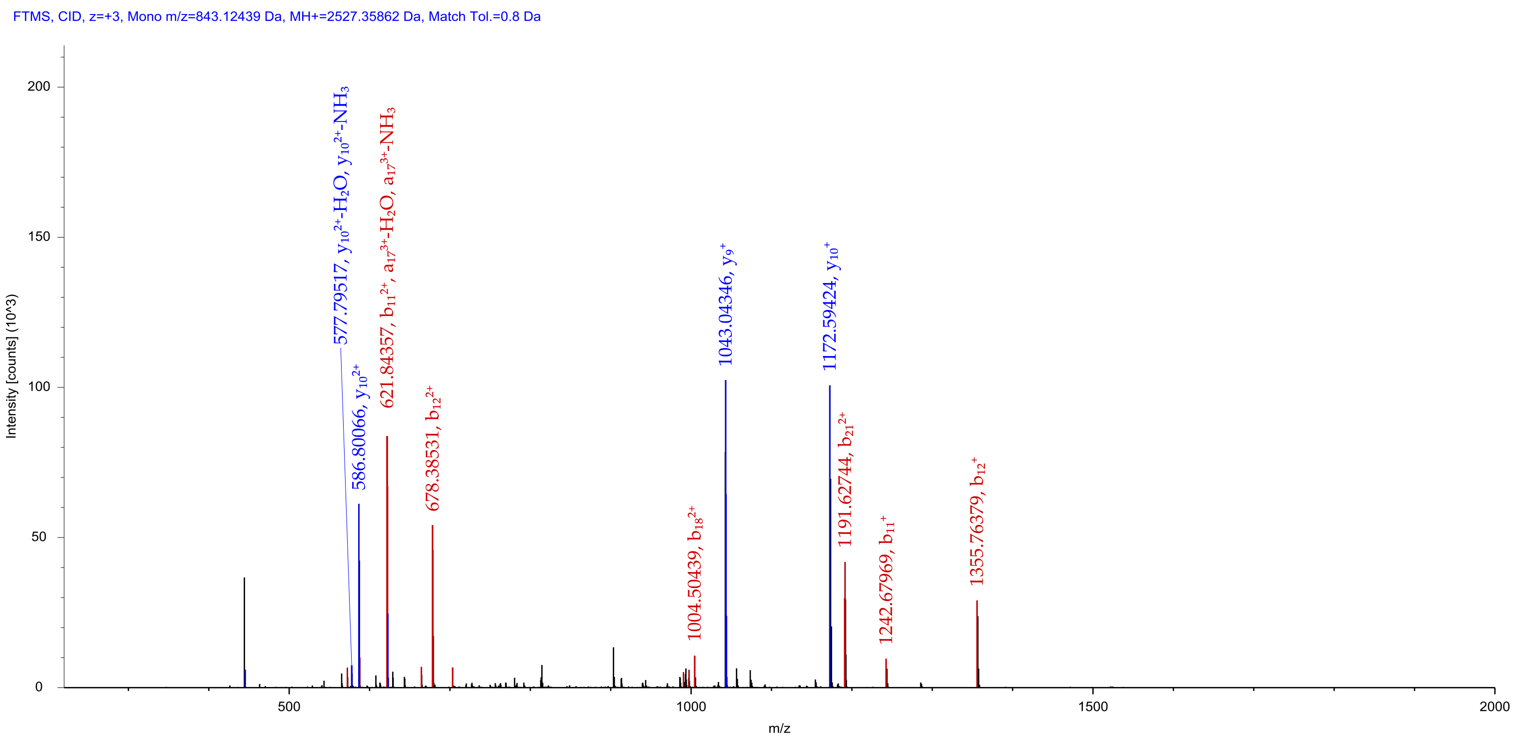

M/Z	Intensity	Matches
572.3098	6636.6	b (10) (2+)
1242.68	9609.1	b (11) (1+)
1355.764	29051.9	b (12) (1+)
678.3853	54127.3	b (12) (2+)
1004.504	10615.7	b (18) (2+)
1191.627	41835.5	b (21) (2+)
997.5391	5919.9	b (8) (1+)-H2O
1172.594	100666.3	y (10) (1+)
586.8007	61208.8	y (10) (2+)
577.7952	7402.7	y (10) (2+)-H2O, y (10) (2+)-NH3
622.846	24636.2	y (17) (3+)
445.2831	5948.9	y (7) (2+)
1043.043	102463.6	y (9) (1+)

LPAGK(ac)AEK(ac)GSGEGK

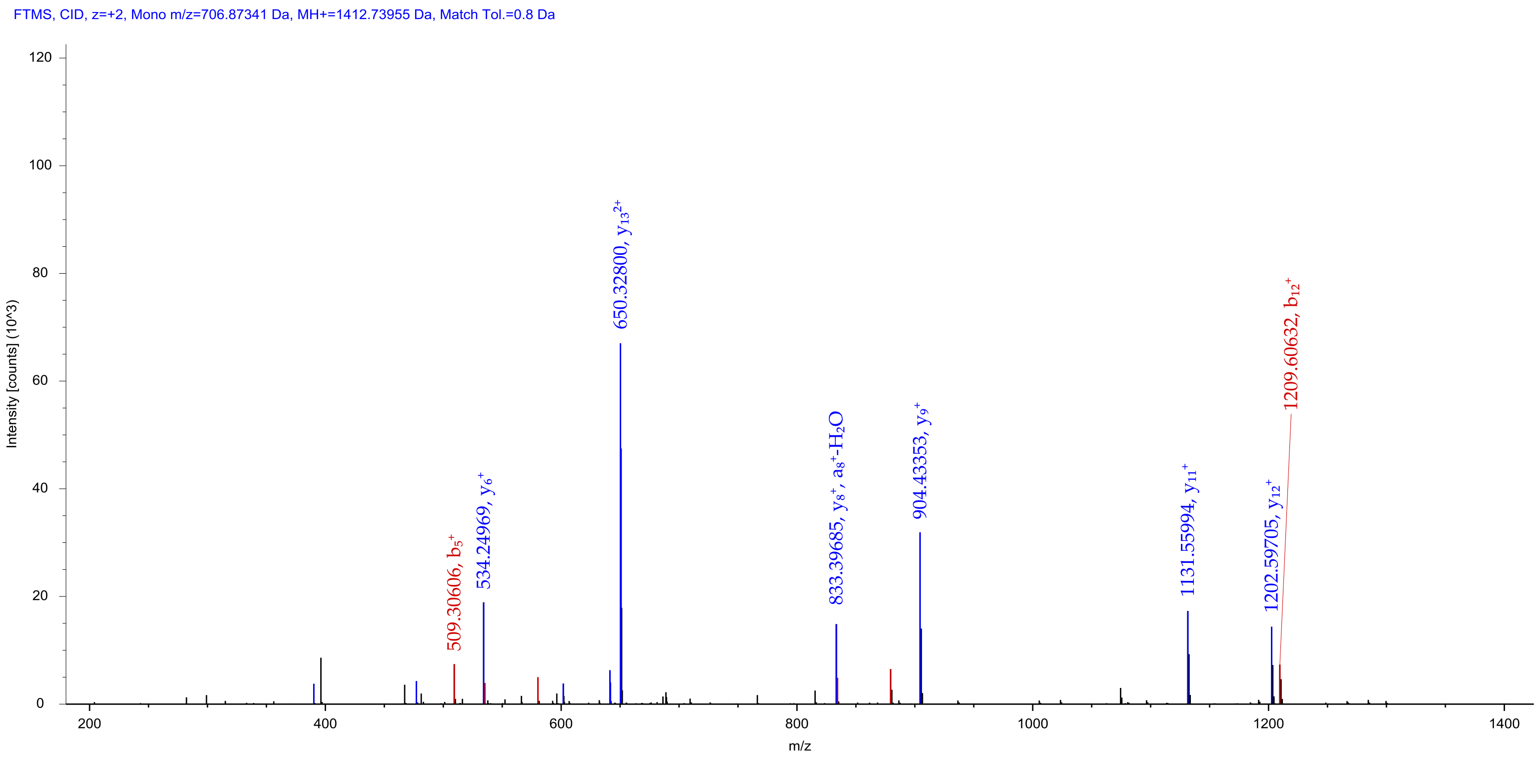

M/Z	Intensity	Matches
1209.606	7338.2	b (12) (1+)
509.3061	7417	b (5) (1+)
580.3428	4988.4	b (6) (1+)
879.4903	6497.4	b (8) (1+)
1131.56	17294.3	y (11) (1+)
1202.597	14368.4	y (12) (1+)
601.802	3824.3	y (12) (2+)
650.328	67014.2	y (13) (2+)
641.3234	6278.5	y (13) (2+)-H2O, y (13) (2+)-NH3
390.1969	3773.1	y (4) (1+)
477.2284	4275.7	y (5) (1+)
534.2497	18911.4	y (6) (1+)
904.4335	31916.4	y (9) (1+)

LPAGK(ac)GEK(ac)GSGEGK

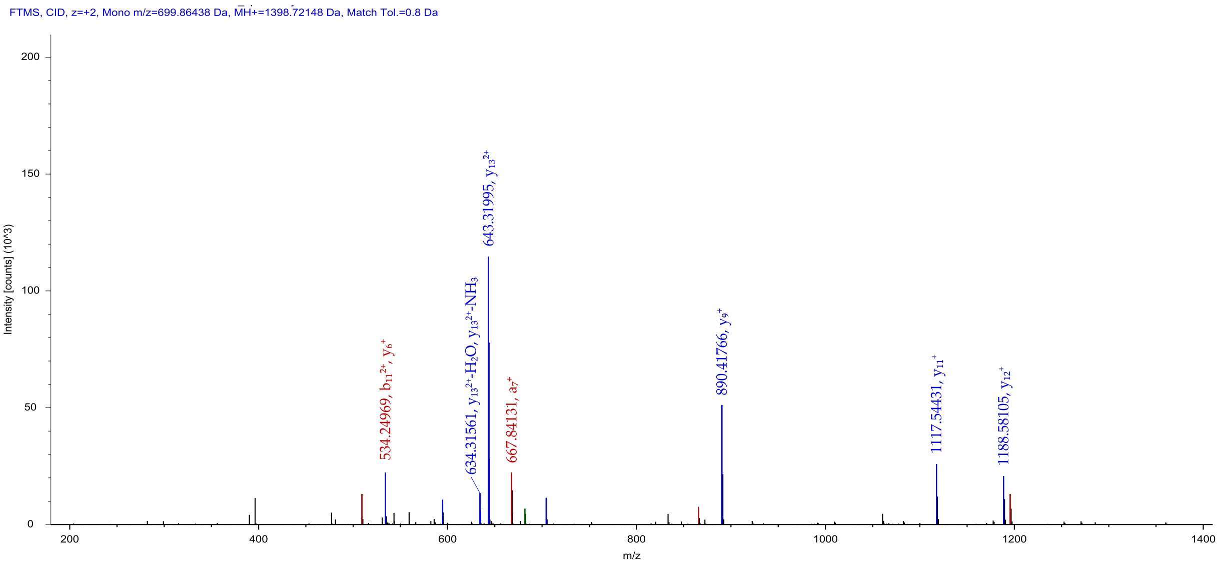

M/Z	Intensity	Matches
534.2497	22291.1	b (11) (2+), y (6) (1+)
1195.591	13059.1	b (12) (1+)
509.306	13106.1	b (5) (1+)
865.4743	7605.6	b (8) (1+)
1117.544	25914.3	y (11) (1+)
1188.581	20773.3	y (12) (1+)
594.794	10617.6	y (12) (2+)
643.32	114652.1	y (13) (2+)
634.3156	13457.4	y (13) (2+)-H2O, y (13) (2+)-NH3
704.3541	11449.5	y (7) (1+)
890.4177	51196.3	y (9) (1+)

LPK(ac)NASSSGLGLTNK(ac)SYTNVSTGEDR

FTMS, CID, z=+3, Mono m/z=941.47272 Da, MH+=2822.40360 Da, Match Tol.=0.8 Da

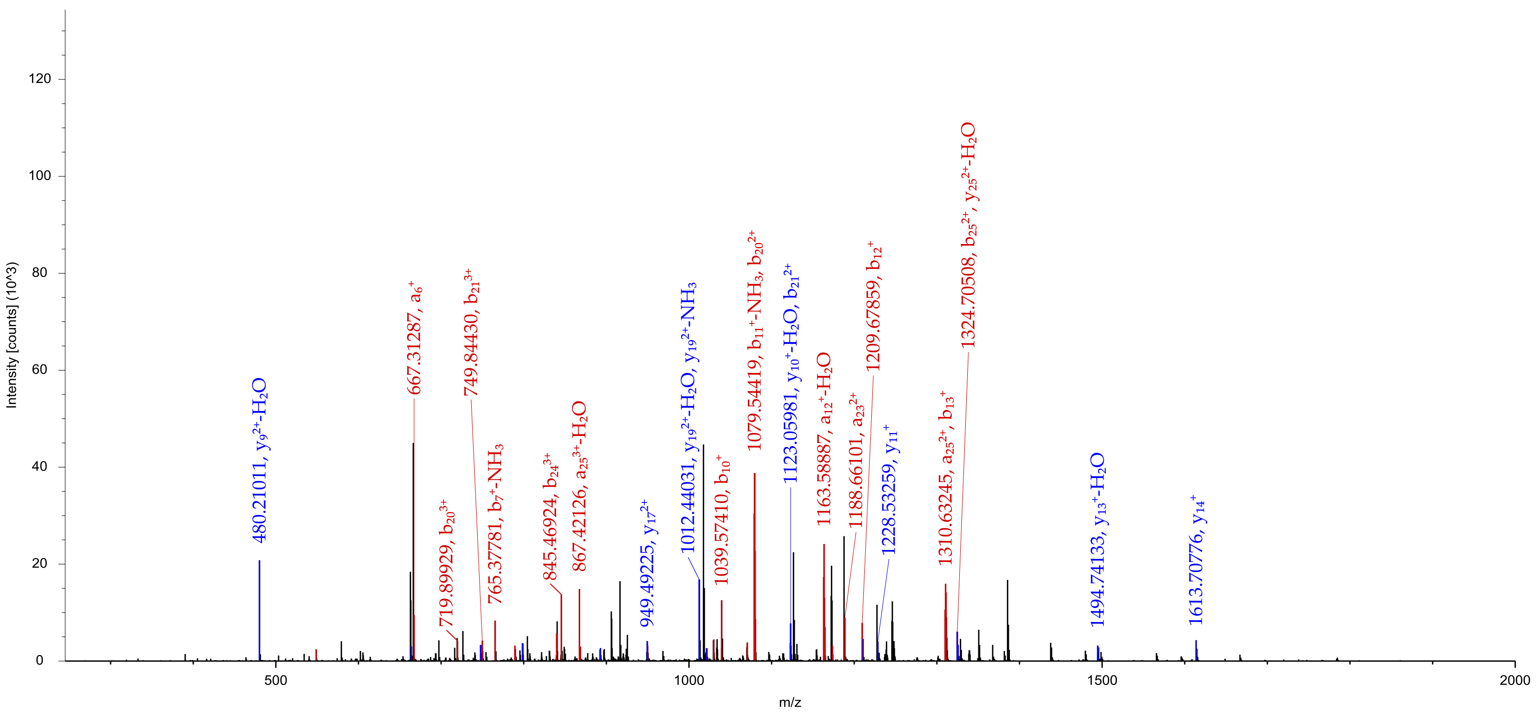

M/Z	Intensity	Matches
1039.574	12542.3	b (10) (1+)
1079.043	30448.3	b (11) (1+)-H2O
1079.544	38790.2	b (11) (1+)-NH3, b (20) (2+)
548.801	2420.7	b (11) (2+)
1209.679	7871.6	b (12) (1+)
789.4271	3166.5	b (15) (2+)-NH3, b (15) (2+)-H2O
1030.009	4459	b (19) (2+)
1070.538	3842.8	b (20) (2+)-H2O
719.8993	4169.3	b (20) (3+)
1123.06	7765.2	b (21) (2+), y (10) (1+)-H2O
749.8443	4199.5	b (21) (3+)
1173.595	3146.6	b (22) (2+)
1164.59	6962.5	b (22) (2+)-H2O
845.4692	13747.4	b (24) (3+)
839.9506	5745.6	b (24) (3+)-NH3
765.3778	8336.7	b (7) (1+)-NH3
1228.533	4033.9	y (11) (1+)
1210.682	4558.9	y (11) (1+)-H2O
1494.741	3193.9	y (13) (1+)-H2O

1495.745	2805.6	y (13) (1+)-NH3
747.909	3326.1	y (13) (2+)-NH3, y (13) (2+)-H2O
1613.708	4293.6	y (14) (1+)
798.4326	3657.9	y (14) (2+)-H2O, b (15) (2+), y (14) (2+)-NH3
892.9116	2683.7	y (16) (2+)
949.4923	4118	y (17) (2+)
1021.494	2634	y (19) (2+), b (10) (1+)-H2O, b (19) (2+)-NH3, b (19) (2+)-H2O
1012.44	16806.8	y (19) (2+)-NH3, y (19) (2+)-H2O
1324.705	6031.1	y (25) (2+)-H2O, b (25) (2+)
1325.708	3346.3	y (25) (2+)-NH3
663.8592	3001.3	y (6) (1+)
480.2101	20793.4	y (9) (2+)-H2O

MQAAK(ac)ISQLEK(ac)QIR

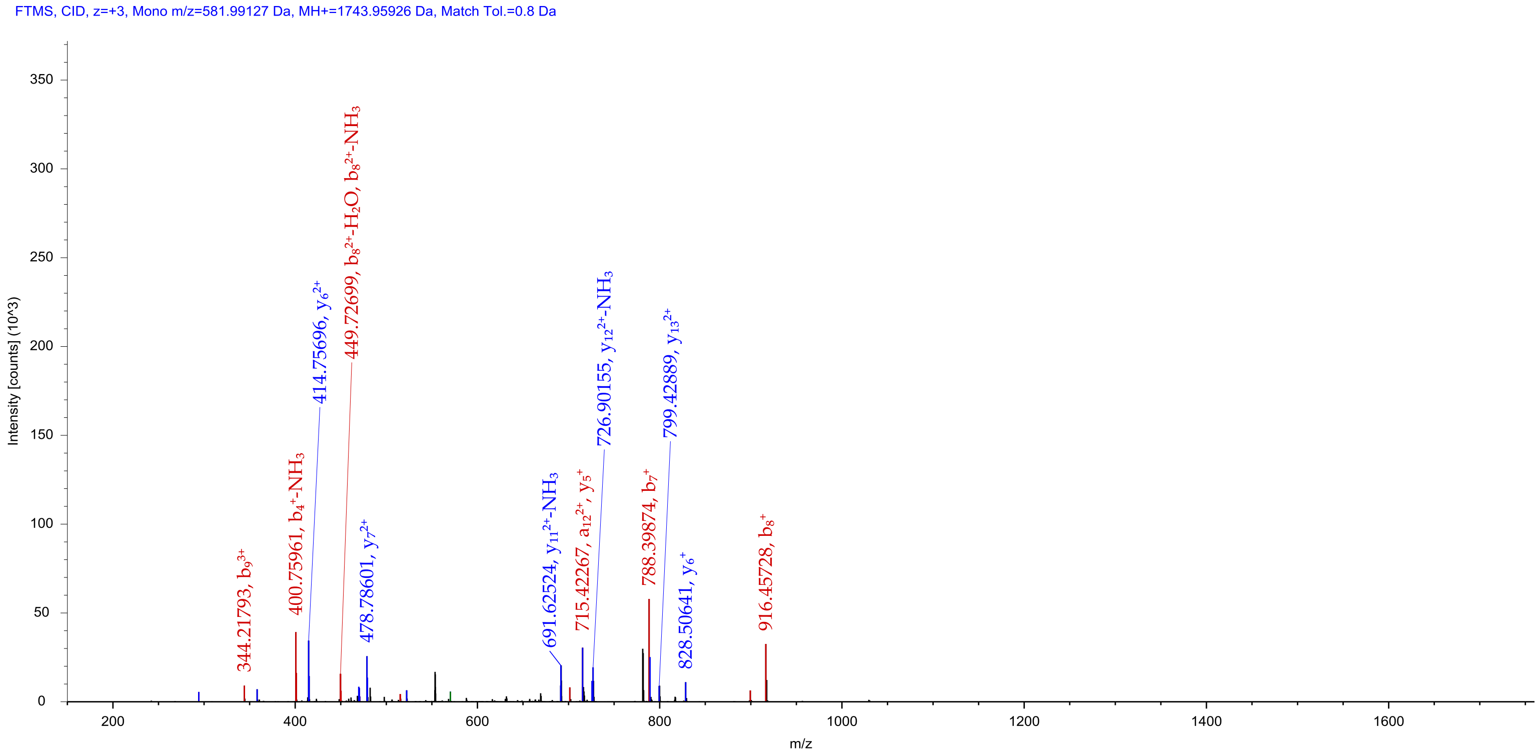

M/Z	Intensity	Matches
400.7596	39227.5	b (4) (1+)-NH3
701.3666	8109.9	b (6) (1+)
788.3987	57898.6	b (7) (1+)
916.4573	32564.6	b (8) (1+)
899.4301	6389.5	b (8) (1+)-NH3
449.727	15732.6	b (8) (2+)-NH3, b (8) (2+)-H2O
344.2179	9096.7	b (9) (3+)
691.6252	20395	y (11) (2+)-NH3
725.8947	11748.7	y (12) (2+)-H2O
726.9016	19385.7	y (12) (2+)-NH3
799.4289	8870.1	y (13) (2+)
789.4018	25096.9	y (13) (2+)-H2O
294.1805	5548.5	y (4) (2+), b (5) (2+)
358.215	7046.2	y (5) (2+)
828.5064	11039.9	y (6) (1+)
414.757	34453.3	y (6) (2+)
478.786	25663.5	y (7) (2+)
469.7806	8397.4	y (7) (2+)-NH3, y (7) (2+)-H2O
522.3017	6464.2	y (8) (2+)

NNKTMAVCK(ac)NAKGTATGCLK(ac)

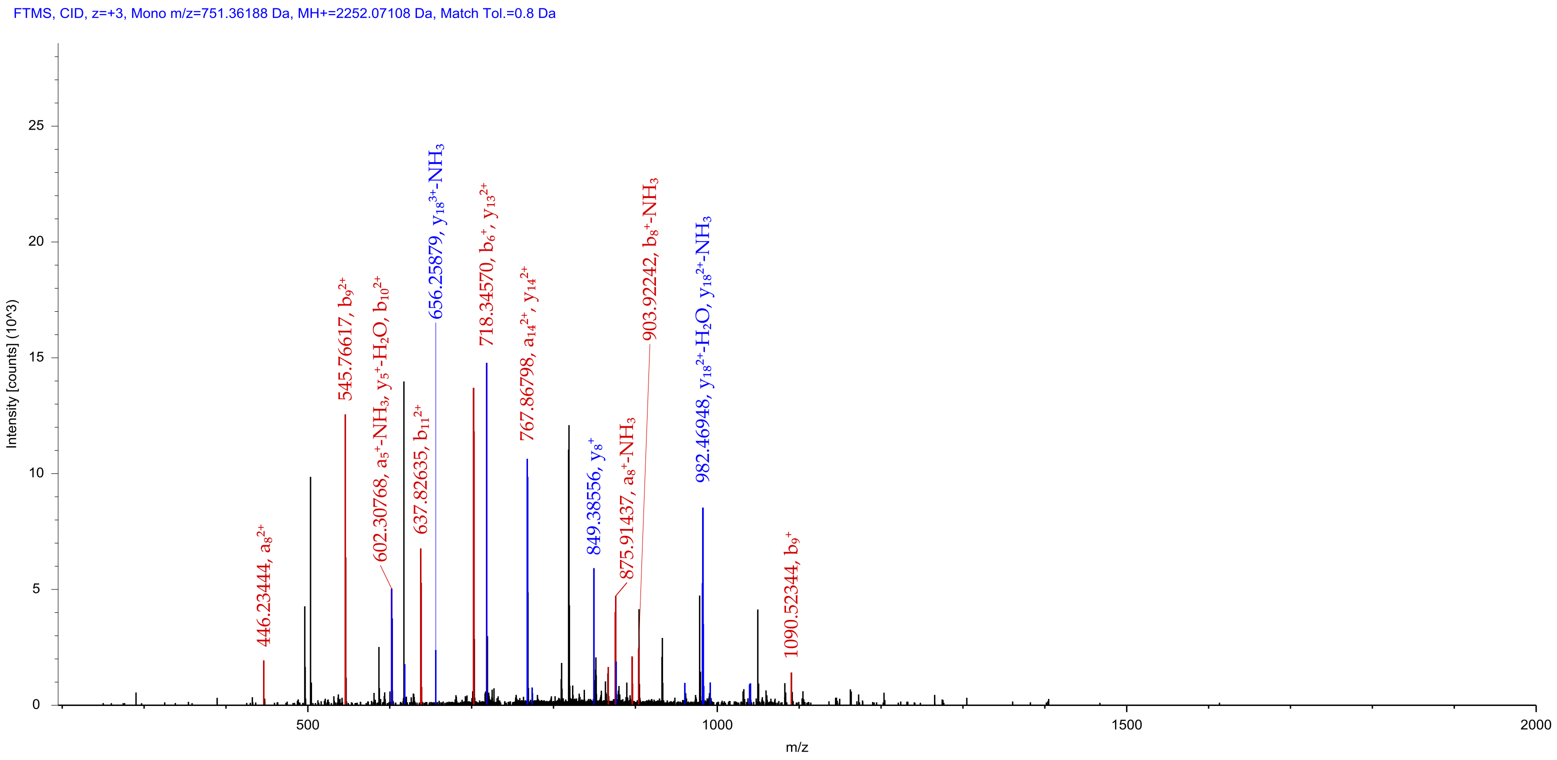

M/Z	Intensity	Matches
637.8264	6768.3	b (11) (2+)
702.3478	13704.1	b (12) (2+)
866.9075	1654.1	b (16) (2+)
895.9244	2110.1	b (17) (2+)
903.9224	2446.6	b (8) (1+)-NH3
1090.523	1412.8	b (9) (1+)
545.7662	12555.4	b (9) (2+)
718.3457	14779.5	y (13) (2+), b (6) (1+)
767.868	10641.4	y (14) (2+), a (14) (2+)
876.4143	1886	y (16) (2+)
618.301	1769.4	y (17) (3+)
991.4549	986.2	y (18) (2+)
982.4695	8527.8	y (18) (2+)-H2O, y (18) (2+)-NH3
656.2588	2384.1	y (18) (3+)-NH3
1039.983	923.8	y (19) (2+)-H2O
1040.485	945.6	y (19) (2+)-NH3
602.3077	5033.2	y (5) (1+)-H2O, a (5) (1+)-NH3, b (10) (2+)
602.8095	3749.1	y (5) (1+)-NH3
773.8586	764.6	y (7) (1+)-H2O
849.3856	5916.8	y (8) (1+)
960.4177	965.1	y (9) (1+)-NH3

QGRK(ac)QELVDK

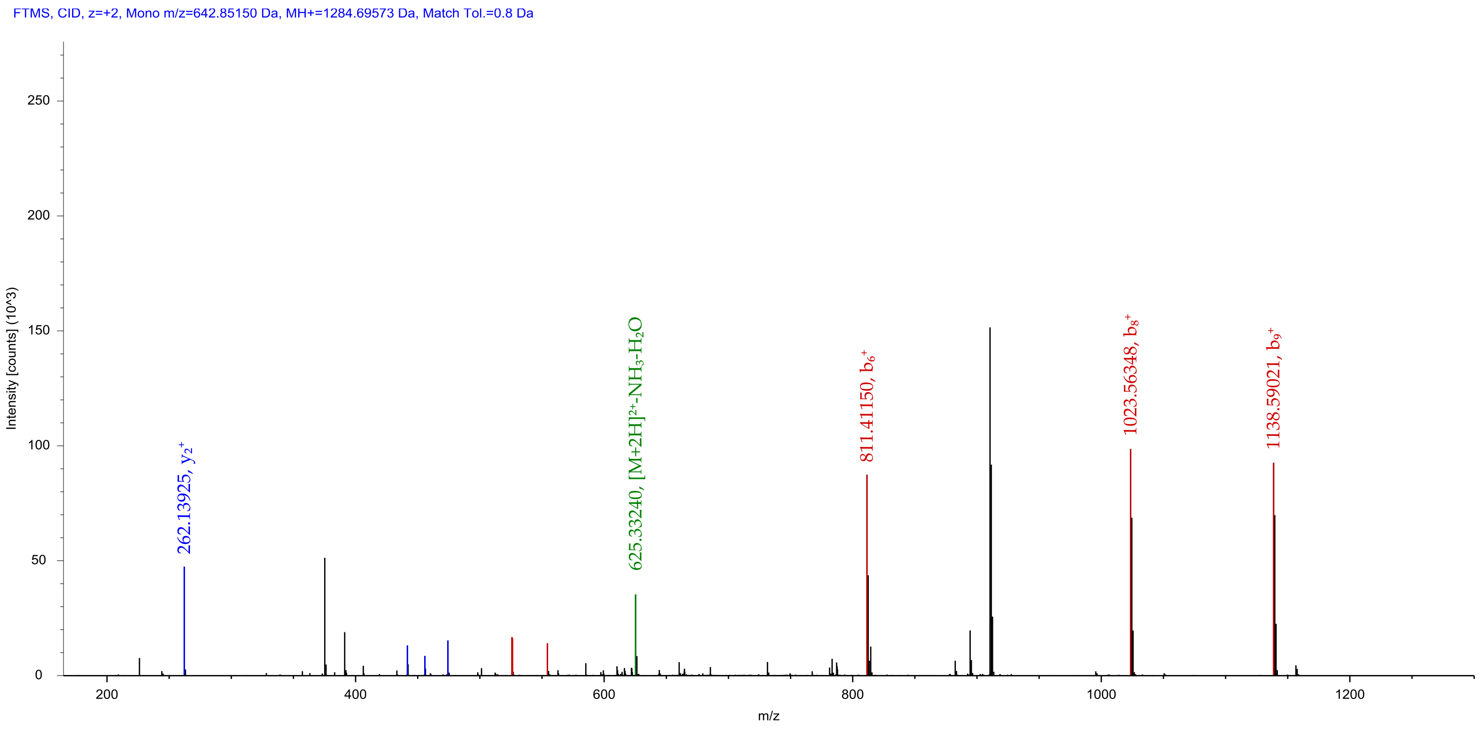

M/Z	Intensity	Matches
554.2957	14084.5	b (4) (1+)
811.4115	87431.5	b (6) (1+)
1023.563	98595.6	b (8) (1+)
1138.59	92662.6	b (9) (1+)
262.1393	47409.3	y (2) (1+)
474.2907	15313.4	y (4) (1+)
455.7434	8601.2	y (4) (1+)-H2O
441.7461	13169.6	y (7) (2+)-H2O

QHTCIPYYKFK(ac)GGEQTRTREK

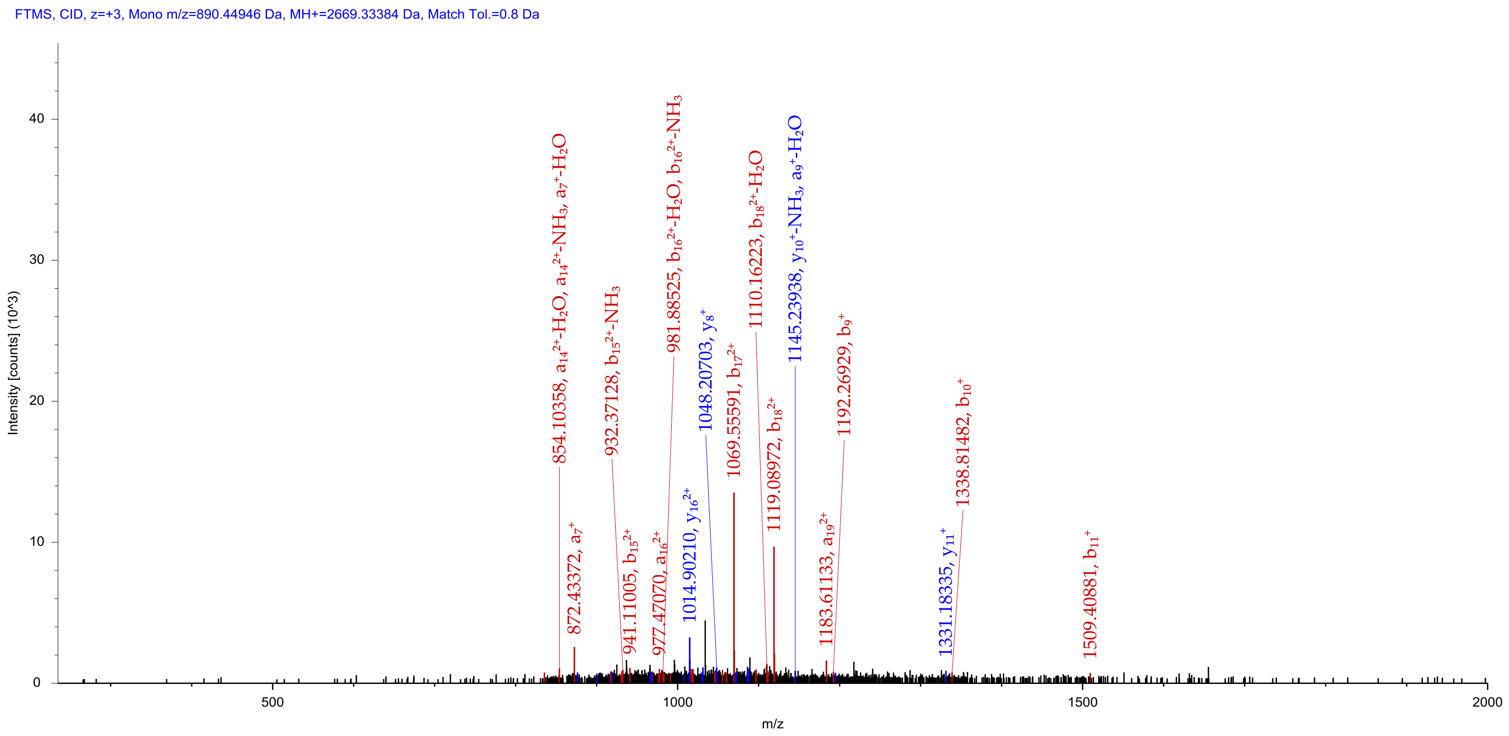

M/Z	Intensity	Matches
1338.815	766.7	b (10) (1+)
1509.409	725.5	b (11) (1+)
876.4423	766.4	b (14) (2+), y (14) (2+)-H2O, y (14) (2+)-NH3
941.1101	1091	b (15) (2+)
931.357	846.6	b (15) (2+)-H2O
932.3713	949.3	b (15) (2+)-NH3
990.9639	716.9	b (16) (2+)
981.8853	790.1	b (16) (2+)-H2O, b (16) (2+)-NH3
1069.556	13519.5	b (17) (2+)
1060.069	743.5	b (17) (2+)-H2O, b (17) (2+)-NH3
1119.09	9687.2	b (18) (2+)
1110.162	1370	b (18) (2+)-H2O
1111.529	844.6	b (18) (2+)-NH3
835.4231	761.5	b (20) (3+)-NH3, b (20) (3+)-H2O
900.1118	678.8	b (7) (1+), y (7) (1+)-H2O
1192.269	810	b (9) (1+)
1145.239	878.8	y (10) (1+)-NH3, a (9) (1+)-H2O
1331.183	885	y (11) (1+)
966.3214	793.3	y (15) (2+)
1014.902	3252	y (16) (2+)
1007.712	690.1	y (16) (2+)-NH3
1072.526	760.2	y (17) (2+)
1194.869	738.1	y (19) (2+)-NH3
918.654	831.3	y (7) (1+), a (15) (2+)-NH3
1048.207	1118.5	y (8) (1+)
1030.195	793	y (8) (1+)-H2O
1031.232	1127.9	y (8) (1+)-NH3
1086.248	1165.1	y (9) (1+)-H2O
1087.845	1040.3	y (9) (1+)-NH3

QTGQQK(ac)GGASRKAR

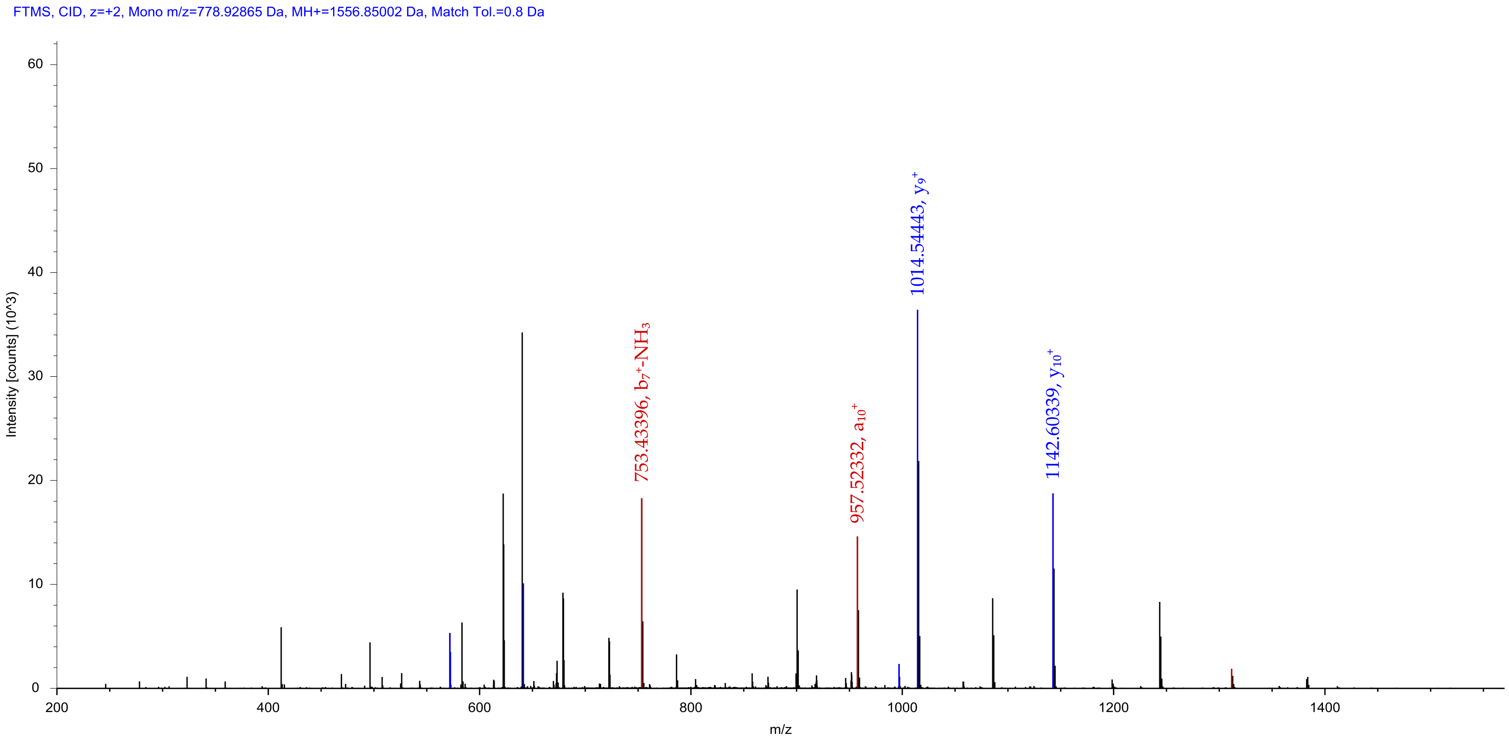

M/Z	Intensity	Matches
1311.701	1881.4	b (12) (1+)
753.434	18288.4	b (7) (1+)-NH3
1142.603	18756.2	y (10) (1+)
571.8051	5335.6	y (4) (1+), y (10) (2+), b (11) (2+)
641.3529	10095.6	y (5) (1+)-H2O
1014.544	36422.3	y (9) (1+)
996.9716	2337.8	y (9) (1+)-H2O, y (9) (1+)-NH3

RFK(ac)ASK(ac)SSGDNSSLR

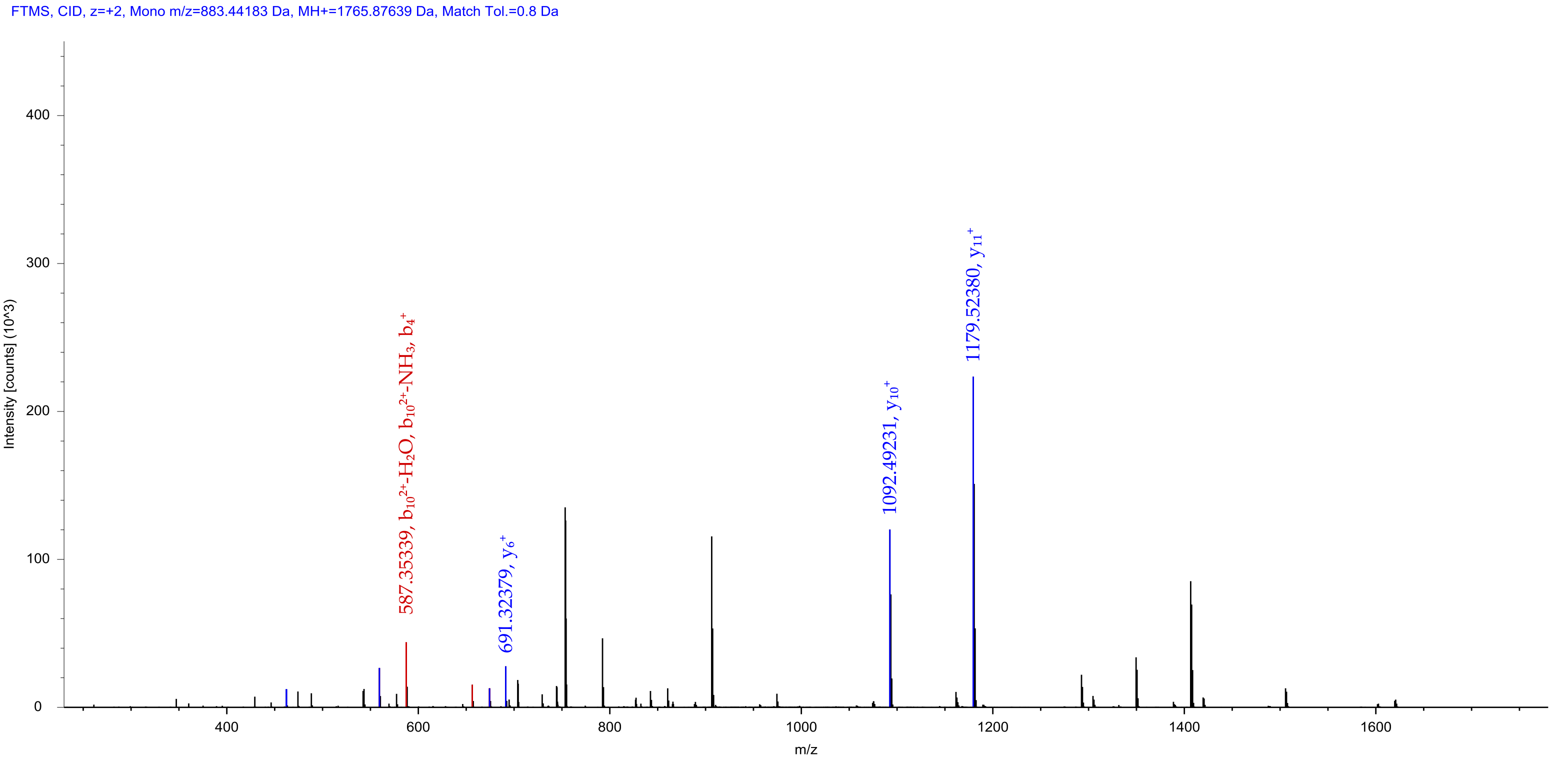

M/Z	Intensity	Matches
587.3532	23159.8	b (10) (2+)-NH3, b (10) (2+)-H2O, b (4) (1+)
656.3742	9137.5	b (5) (1+)-H2O
1092.492	95445.2	y (10) (1+)
1179.524	178318	y (11) (1+)
462.254	11588.4	y (4) (1+), y (9) (2+)
691.3236	22551.1	y (6) (1+)

RIK(ac)PESKPLKELK

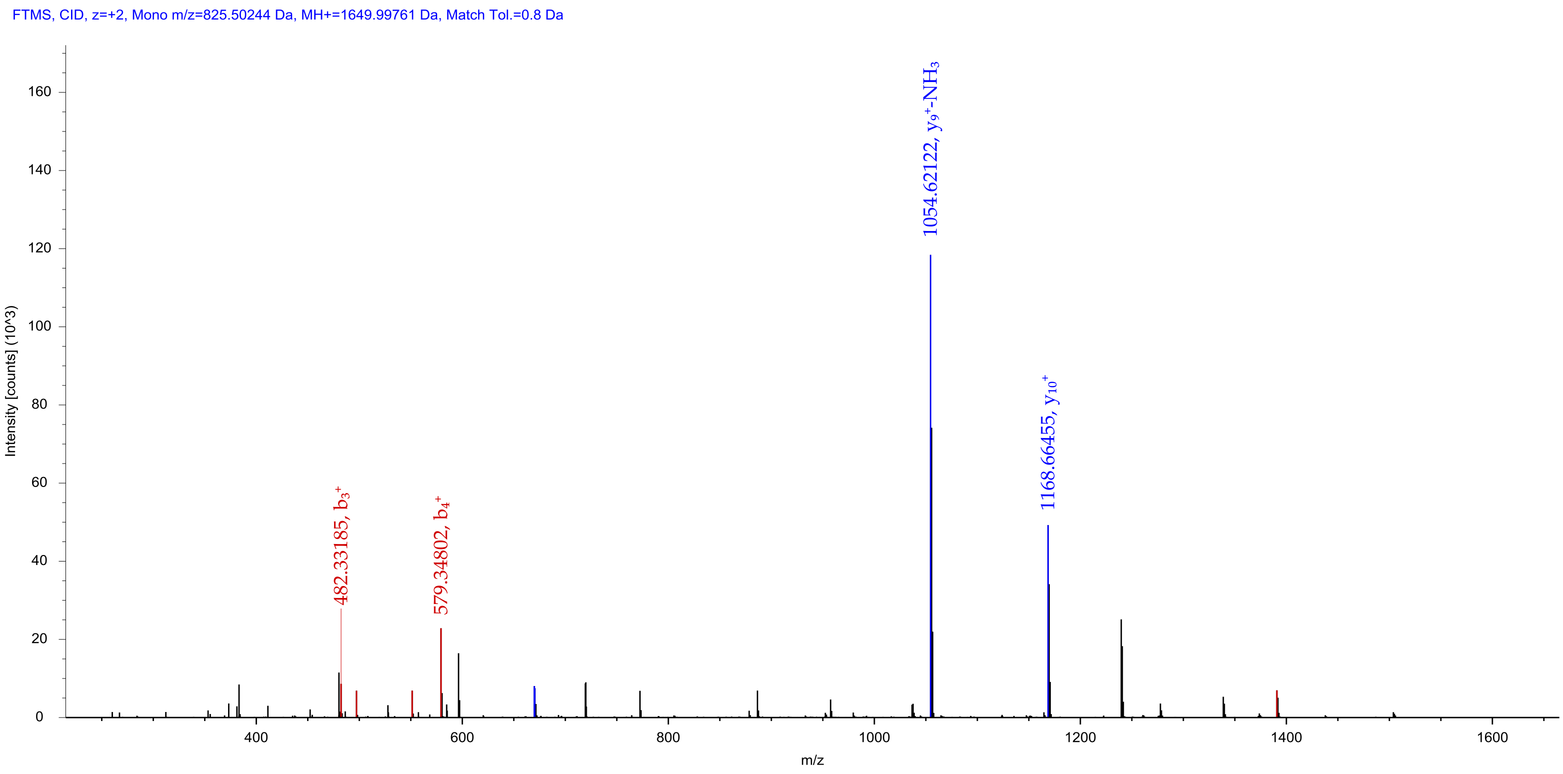

M/Z	Intensity	Matches
1390.8	6962.2	b (11) (1+)
482.3319	8630.5	b (3) (1+)
579.348	22849.2	b (4) (1+)
1168.665	49243.3	y (10) (1+)
669.8886	8069.5	y (11) (2+)
1054.621	118408.6	y (9) (1+)-NH3

TIK(ac)ILLR

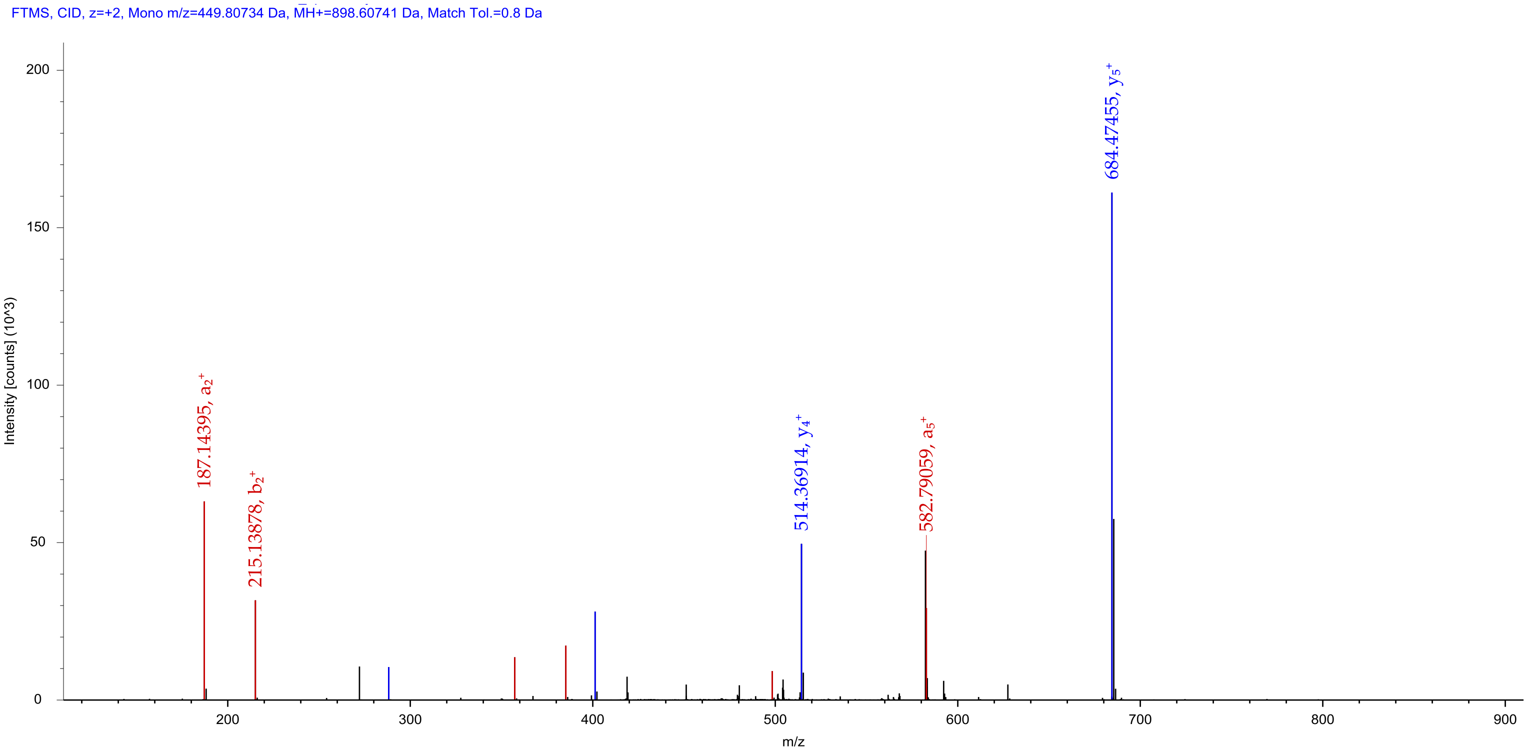

M/Z	Intensity	Matches
215.1388	31683	b (2) (1+)
385.2437	17282.3	b (3) (1+)
498.3272	9188.1	b (4) (1+)
288.2026	10457.9	y (2) (1+)
401.2861	28091.6	y (3) (1+)
514.3691	49606.9	y (4) (1+)
684.4746	161175.4	y (5) (1+)

TPPEK(ac)SWITWYK

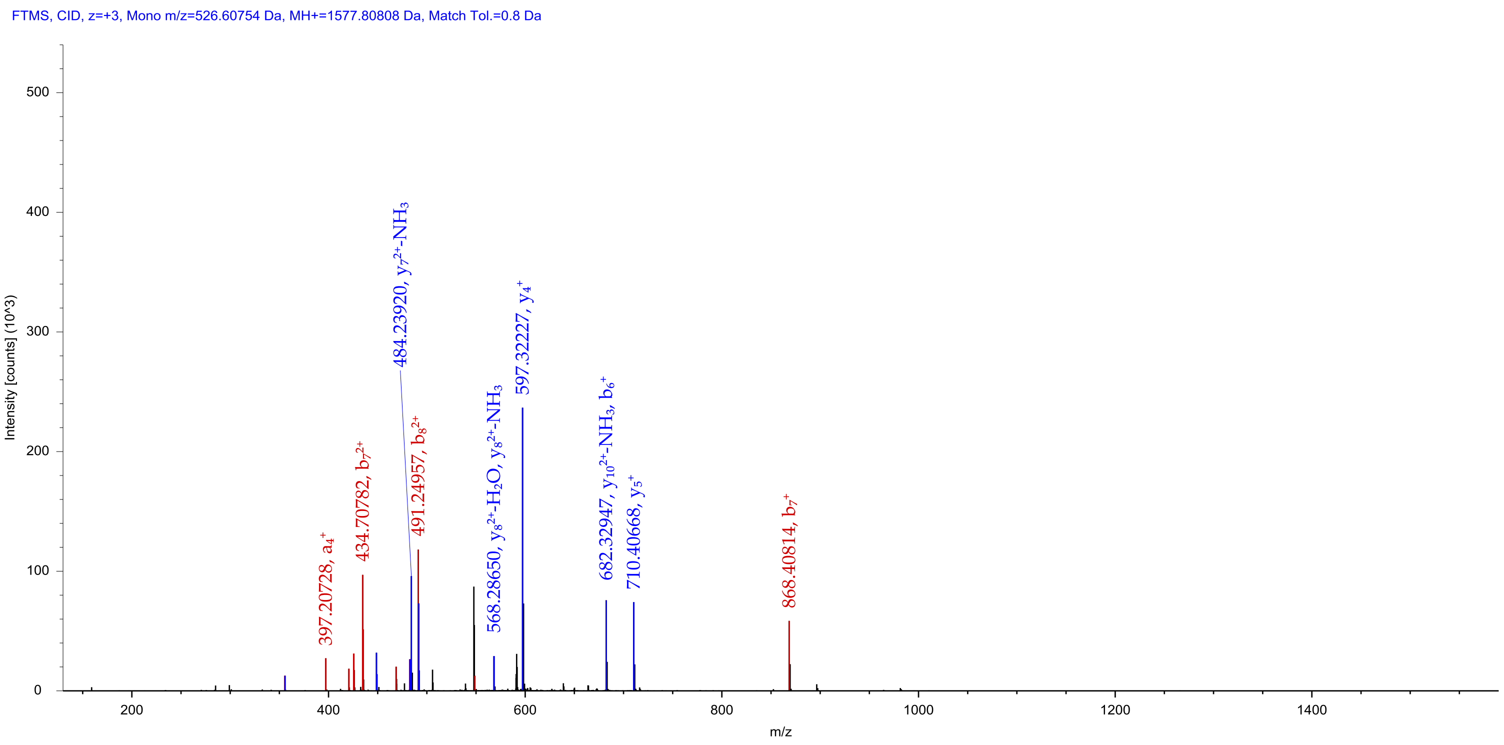

M/Z	Intensity	Matches
425.7027	7336.9	b (4) (1+), b (7) (2+)-H2O, b (7) (2+)-NH3
682.3298	17406.9	b (6) (1+), y (10) (2+)-NH3
868.4077	12704.7	b (7) (1+)
434.7079	26263.5	b (7) (2+)
491.2494	29490.8	b (8) (2+)
482.7365	4296.2	b (8) (2+)-NH3, b (8) (2+)-H2O, y (7) (2+)-H2O
486.9413	3028.6	y (11) (3+)-NH3, y (11) (3+)-H2O
597.3218	57556.4	y (4) (1+)
579.0062	3164.9	y (4) (1+)-H2O, b (5) (1+)-NH3
710.4067	18591.4	y (5) (1+)
448.7465	5742.9	y (6) (2+)
491.7506	15408.7	y (7) (2+)
484.2393	24166.2	y (7) (2+)-NH3
568.2864	4155.6	y (8) (2+)-NH3, y (8) (2+)-H2O

TVDGVLLKCLGPEEAK(ac)TVMSEVHEGICGTHQSAHKMK

FTMS, CID, z=+3, Mono m/z=1387.69275 Da, MH+=4161.06369 Da, Match Tol.=0.8 Da

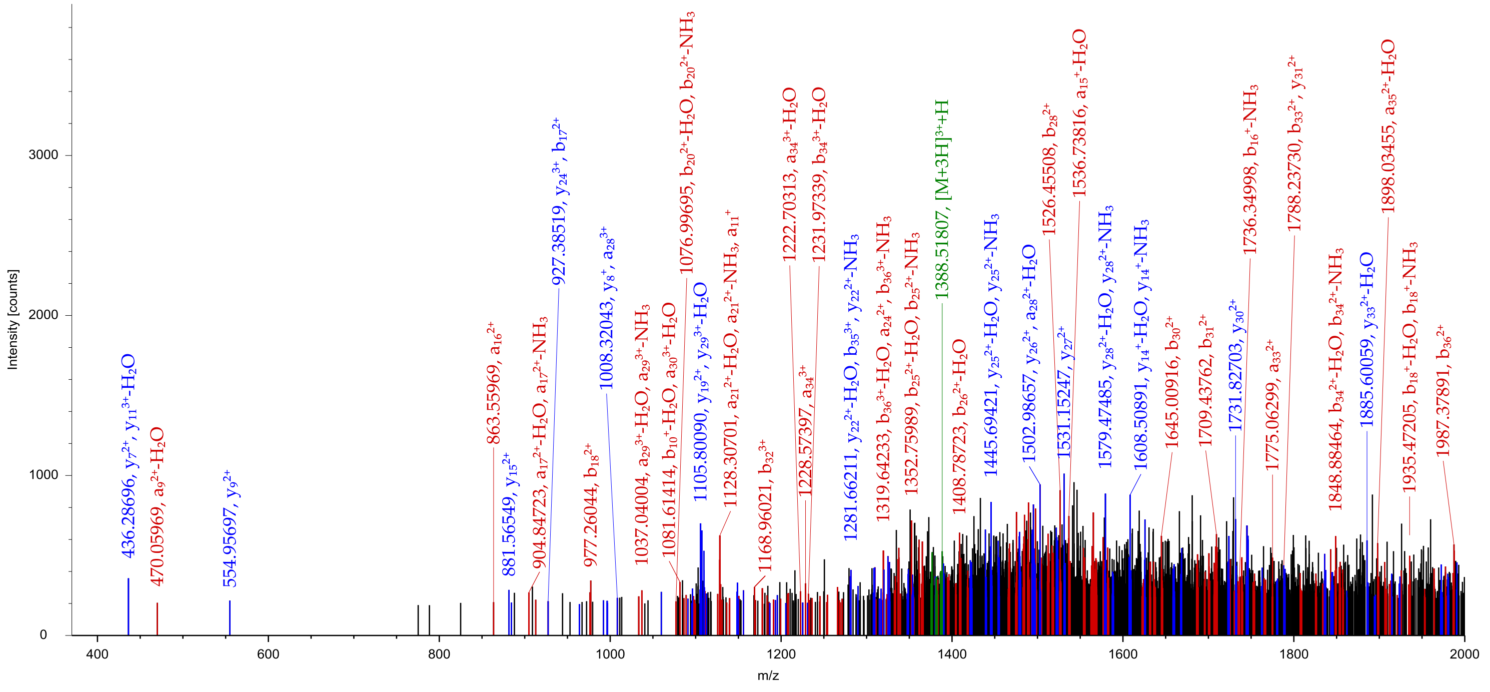

M/Z	Intensity	Matches
1098.881	223	b (10) (1+)
1081.614	333.7	b (10) (1+)-H2O, a (30) (3+)-H2O
1155.929	282.4	b (11) (1+), y (30) (3+)
1139.766	233.4	b (11) (1+)-NH3, b (31) (3+)
1236.257	233.3	b (12) (1+)-NH3, b (12) (1+)-H2O
1383.389	350	b (13) (1+)
1365.27	587.6	b (13) (1+)-H2O, b (13) (1+)-NH3
1512.458	635.8	b (14) (1+), a (28) (2+)
1495.224	819.4	b (14) (1+)-NH3, y (26) (2+)-NH3
1582.259	549.7	b (15) (1+)
1565.362	768.8	b (15) (1+)-H2O, b (15) (1+)-NH3
1753.142	337.2	b (16) (1+)
1734.619	338.7	b (16) (1+)-H2O
1736.35	366.9	b (16) (1+)-NH3
1854.215	460.3	b (17) (1+)
1836.05	510.3	b (17) (1+)-H2O, y (32) (2+)-H2O
1837.377	409	b (17) (1+)-NH3, y (32) (2+)-NH3
1953.061	291.5	b (18) (1+)
1935.472	498	b (18) (1+)-NH3, b (18) (1+)-H2O

977.2604	343.8	b (18) (2+)
1033.408	244.6	b (19) (2+)-NH3, b (19) (2+)-H2O
1086.37	233.2	b (20) (2+)
1076.997	213.2	b (20) (2+)-H2O, b (20) (2+)-NH3
1150.568	247.4	b (21) (2+)
1199.478	229.8	b (22) (2+)
1191.093	224.5	b (22) (2+)-NH3, b (22) (2+)-H2O
1268.191	220.5	b (23) (2+)
1333.819	371.3	b (24) (2+)
1323.963	257.8	b (24) (2+)-H2O
1361.435	599	b (25) (2+)
1352.76	718.7	b (25) (2+)-NH3, b (25) (2+)-H2O
1418.437	405.8	b (26) (2+)
1408.787	642.7	b (26) (2+)-H2O
1410.394	485.2	b (26) (2+)-NH3
1497.736	793.4	b (27) (2+)
1489.464	831.6	b (27) (2+)-H2O, b (27) (2+)-NH3
1526.455	908	b (28) (2+)
1517.006	562.7	b (28) (2+)-H2O
1518.456	516.6	b (28) (2+)-NH3
1576.78	614.6	b (29) (2+)
1568.337	482.4	b (29) (2+)-H2O, b (29) (2+)-NH3
1645.009	622.9	b (30) (2+)
1636.001	462.2	b (30) (2+)-H2O
1636.527	351.3	b (30) (2+)-NH3
1097.618	246.5	b (30) (3+)
1090.916	230.1	b (30) (3+)-H2O, y (9) (1+)-H2O
1709.438	633.6	b (31) (2+)
1700.597	513.6	b (31) (2+)-H2O, b (31) (2+)-NH3
1753.92	387.1	b (32) (2+)
1745.113	687.3	b (32) (2+)-H2O, y (15) (1+)-H2O, b (32) (2+)-NH3, y (15) (1+)-NH3
1168.96	303.8	b (32) (3+)
1779.802	342.7	b (33) (2+)-H2O, y (31) (2+)-H2O
1780.936	356	b (33) (2+)-NH3, y (31) (2+)-NH3
1857.289	412.7	b (34) (2+)
1848.885	620.7	b (34) (2+)-NH3, b (34) (2+)-H2O
1231.973	260.9	b (34) (3+)-H2O
1233.523	221.7	b (34) (3+)-NH3
1920.923	253.5	b (35) (2+)
1911.802	322.3	b (35) (2+)-H2O
1913.352	342.5	b (35) (2+)-NH3

1987.379	568.9	b (36) (2+)
1977.425	431.5	b (36) (2+)-H2O
1977.86	306.8	b (36) (2+)-NH3
1319.642	530.9	b (36) (3+)-NH3, a (24) (2+), b (36) (3+)-H2O
1148.839	329.6	y (10) (1+)-H2O, y (30) (3+)-H2O, y (10) (1+)-NH3, y (30) (3+)-NH3
1326.777	459.8	y (11) (1+)
1308.557	422.2	y (11) (1+)-H2O
1309.705	425.9	y (11) (1+)-NH3, a (36) (3+)-NH3, a (36) (3+)-H2O, a (24) (2+)-H2O
436.287	357.6	y (11) (3+)-H2O, y (7) (2+)
1439.037	662.2	y (12) (1+)
1421.407	470.2	y (12) (1+)-H2O
1422.939	458.7	y (12) (1+)-NH3
1496.128	543.3	y (13) (1+)
1478.972	647.8	y (13) (1+)-NH3, y (13) (1+)-H2O
1625.788	725.5	y (14) (1+)
1608.509	879.7	y (14) (1+)-NH3, y (14) (1+)-H2O
1762.949	424.1	y (15) (1+)
881.5655	285.1	y (15) (2+)
1862.081	280.8	y (16) (1+)
1844.647	394.9	y (16) (1+)-H2O
1845.181	395.7	y (16) (1+)-NH3, y (32) (2+)
1990.81	457.3	y (17) (1+)
1973.385	271.1	y (17) (1+)-NH3
1105.801	700.8	y (19) (2+), y (29) (3+)-H2O
1096.34	293.5	y (19) (2+)-H2O, y (19) (2+)-NH3
1205.488	203.4	y (21) (2+)
1195.579	252.3	y (21) (2+)-H2O
1290.044	309.6	y (22) (2+)
1281.662	410	y (22) (2+)-NH3, y (22) (2+)-H2O, b (35) (3+)
1325.009	495.6	y (23) (2+), b (24) (2+)-NH3, b (36) (3+)
1317.103	292.1	y (23) (2+)-H2O, y (23) (2+)-NH3
884.4235	206.3	y (23) (3+)
1389.904	450.3	y (24) (2+)
1380.563	369.9	y (24) (2+)-H2O
927.3852	214.1	y (24) (3+), b (17) (2+)
1454.024	592.3	y (25) (2+)
1445.694	834.1	y (25) (2+)-NH3, y (25) (2+)-H2O
964.0527	195.5	y (25) (3+)-H2O, y (25) (3+)-NH3
1502.987	944.1	y (26) (2+), a (28) (2+)-H2O
1493.945	661.1	y (26) (2+)-H2O, b (14) (1+)-H2O
996.4645	216.6	y (26) (3+)-NH3, y (17) (2+), y (26) (3+)-H2O

1531.152	1011.8	y (27) (2+)
1521.978	674.2	y (27) (2+)-H2O
1522.63	614.4	y (27) (2+)-NH3
1587.77	398.6	y (28) (2+)
1579.475	886.7	y (28) (2+)-H2O, y (28) (2+)-NH3
1059.817	272.8	y (28) (3+)
1668.794	546.1	y (29) (2+)
1659.833	445.3	y (29) (2+)-H2O, y (29) (2+)-NH3
1112.725	262.6	y (29) (3+)
1107.168	656.9	y (29) (3+)-NH3
1731.827	727.7	y (30) (2+)
1723.959	621.7	y (30) (2+)-H2O, y (30) (2+)-NH3
1788.237	438.8	y (31) (2+), b (33) (2+)
1193.279	222.2	y (31) (3+), b (33) (3+)
1186.686	203.3	y (31) (3+)-H2O, y (31) (3+)-NH3, b (33) (3+)-NH3, b (33) (3+)-H2O
1230.961	204.6	y (32) (3+)
1225.257	206.4	y (32) (3+)-H2O, y (32) (3+)-NH3, a (12) (1+)
1895.503	255	y (33) (2+)
1885.601	594.5	y (33) (2+)-H2O
1886.099	333.5	y (33) (2+)-NH3
1924.18	369	y (34) (2+)
1914.584	385.8	y (34) (2+)-H2O, y (34) (2+)-NH3
1980.178	386	y (35) (2+)
1320.328	411.6	y (35) (3+)
1314.924	230.3	y (35) (3+)-H2O, a (36) (3+), y (35) (3+)-NH3
1353.881	380.3	y (36) (3+)
1348.761	385.1	y (36) (3+)-H2O
1348.899	414.4	y (36) (3+)-NH3
1008.32	235.2	y (8) (1+), a (28) (3+)
992.1196	220	y (8) (1+)-NH3
1109.619	529.3	y (9) (1+)
554.957	218.3	y (9) (2+)

TVDGPSSK(ac)DWR

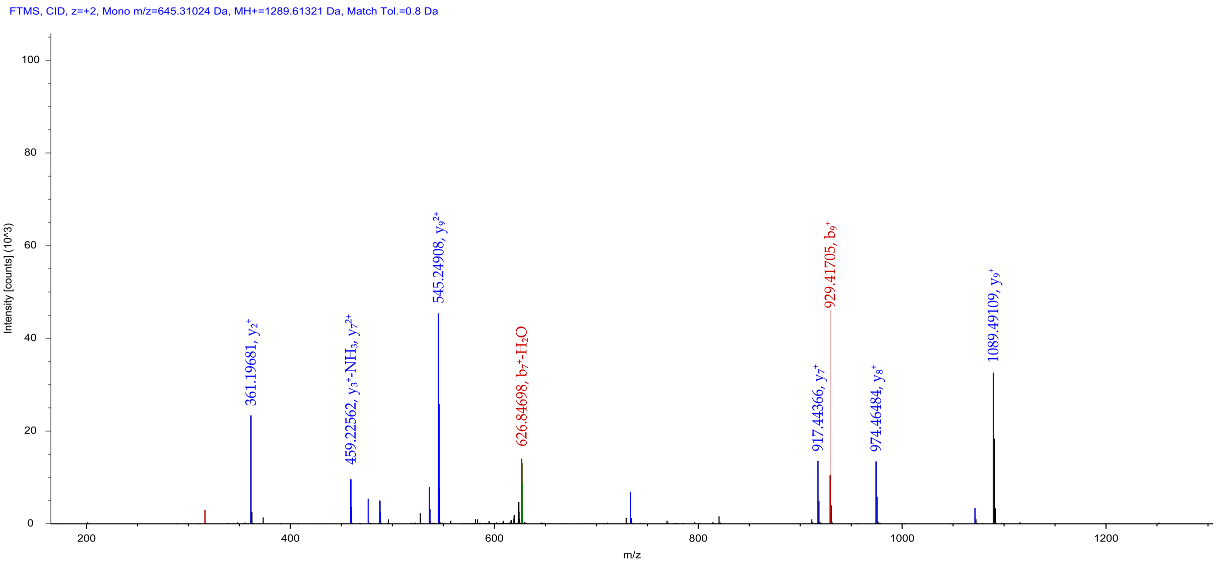

M/Z	Intensity	Matches
316.1492	2989	b (3) (1+)
626.847	14035.1	b (7) (1+)-H2O
929.4171	10466.6	b (9) (1+)
361.1968	23375.7	y (2) (1+)
476.2229	5371.2	y (3) (1+)
459.2256	9588	y (3) (1+)-NH3, y (7) (2+)
733.3594	6887.8	y (5) (1+)
917.4437	13537.4	y (7) (1+)
974.4648	13451	y (8) (1+)
487.736	5015.6	y (8) (2+)
1089.491	32590.1	y (9) (1+)
1071.48	3399	y (9) (1+)-H2O
545.2491	45392.5	y (9) (2+)
536.2441	7879.2	y (9) (2+)-NH3, y (9) (2+)-H2O, a (10) (2+)-NH3

VNCEMIAKYPQATEDNLVHLLKEQHFKTPAESNVYDLMDK(ac)K(ac)

FTMS, CID, z=+4, Mono m/z=1233.84741 Da, MH+=4932.36782 Da, Match Tol.=0.8 Da

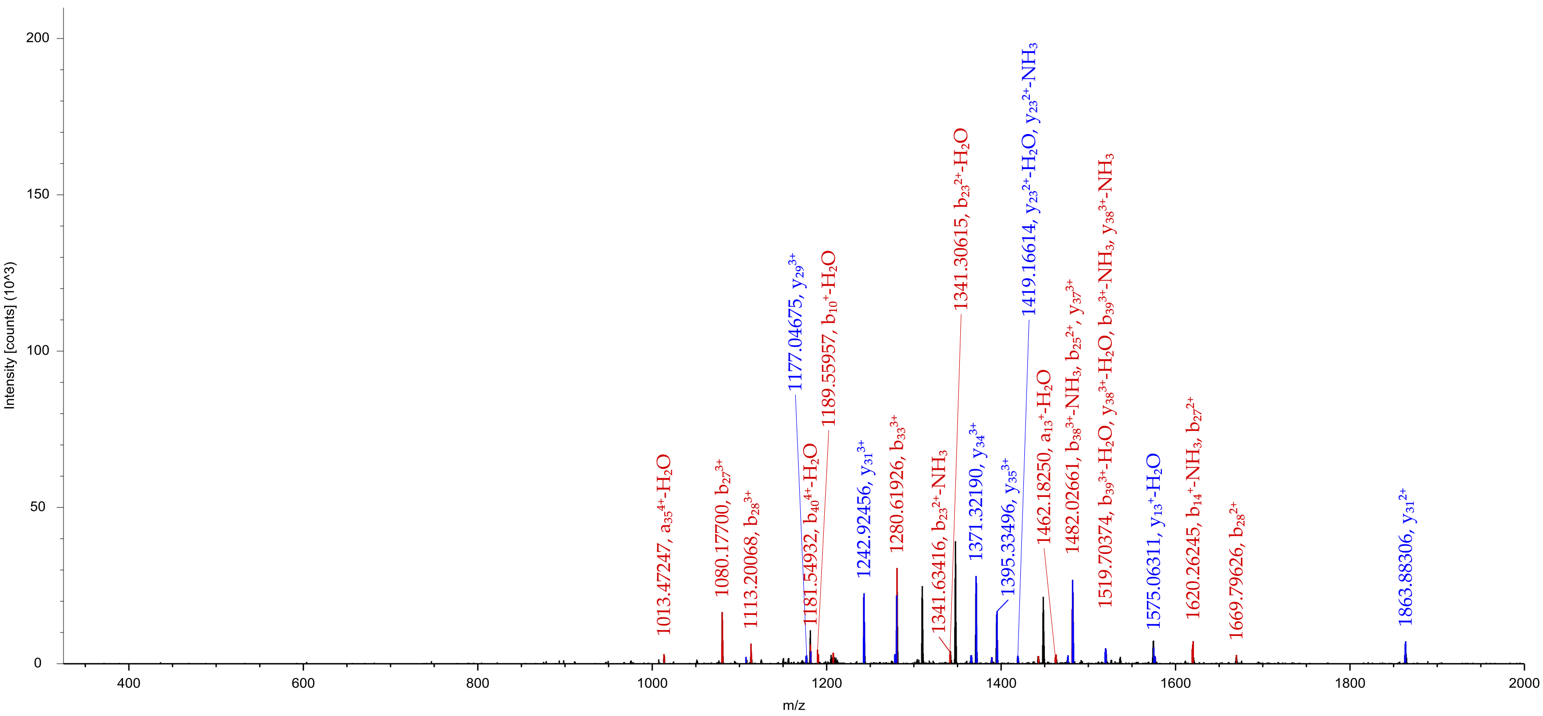

M/Z	Intensity	Matches
1189.56	4505.3	b (10) (1+)-H2O
1190.544	3051.7	b (10) (1+)-NH3
1389.328	2068	b (12) (1+)-H2O, y (35) (3+)-H2O, b (12) (1+)-NH3, y (35) (3+)-NH3
1619.26	4647.2	b (14) (1+)-H2O
1107.531	2191	b (19) (2+), y (37) (4+)-NH3, b (28) (3+)-H2O, y (37) (4+)-H2O, b (28) (3+)-NH3
1341.306	2982.1	b (23) (2+)-H2O
1341.634	3721.4	b (23) (2+)-NH3
1482.027	26838.9	b (25) (2+), b (38) (3+)-NH3, y (37) (3+)
1620.262	7205.4	b (27) (2+), b (14) (1+)-NH3
1080.177	16505.1	b (27) (3+)
1669.796	2832.5	b (28) (2+)
1113.201	6433.2	b (28) (3+)
1207.569	3480.3	b (31) (3+)-NH3, b (10) (1+), b (31) (3+)-H2O, a (21) (2+)
1862.88	2754	b (32) (2+)
1242.591	21313.1	b (32) (3+)
1280.619	30611.7	b (33) (3+)
1442.672	2436.9	b (37) (3+)
1481.692	17434.5	b (38) (3+)-H2O
1519.704	4928.6	b (39) (3+)-NH3, b (39) (3+)-H2O, y (38) (3+)-NH3, y (38) (3+)-H2O
1575.399	2666.8	b (40) (3+)-H2O

1576.246	2455.1	b (40) (3+)-NH3, y (13) (1+)-NH3
1181.549	6161.2	b (40) (4+)-H2O
1278.613	3118.7	y (10) (1+)-H2O
1280.285	21882.6	y (10) (1+)-NH3
1575.063	5129.5	y (13) (1+)-H2O
1181.801	4009	y (19) (2+), b (40) (4+)-NH3
1419.166	2545.9	y (23) (2+)-H2O, y (23) (2+)-NH3
1177.047	2691.6	y (29) (3+)
1863.883	7175.4	y (31) (2+)
1242.925	22544.5	y (31) (3+)
1371.322	28097.5	y (34) (3+)
1365.981	2698.7	y (34) (3+)-NH3, y (34) (3+)-H2O
1395.335	16760.5	y (35) (3+)
1476.686	2699.8	y (37) (3+)-H2O, y (37) (3+)-NH3

YYK(ac)NITPPDCYVLTK

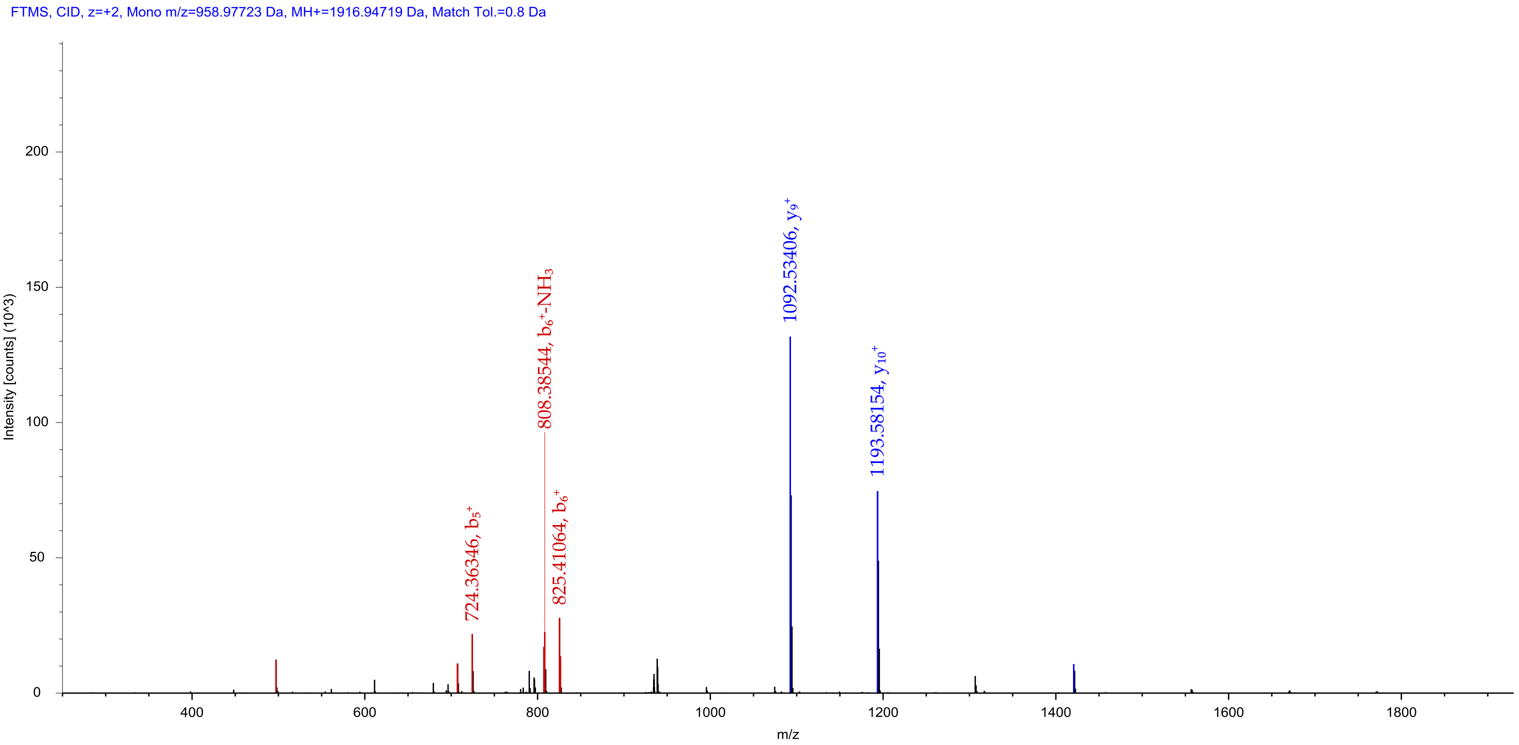

M/Z	Intensity	Matches
826.4134	13616.3	b (13) (2+)-H2O, b (13) (2+)-NH3
497.2372	12418.1	b (3) (1+)
724.3635	21828.3	b (5) (1+)
825.4106	27804.5	b (6) (1+)
807.4005	17067.3	b (6) (1+)-H2O
808.3854	22569.8	b (6) (1+)-NH3
1193.582	74667.6	y (10) (1+)
1420.709	10720.7	y (12) (1+)
1092.534	131749	y (9) (1+)

RCTHCLSYK(Ac)TPQWR

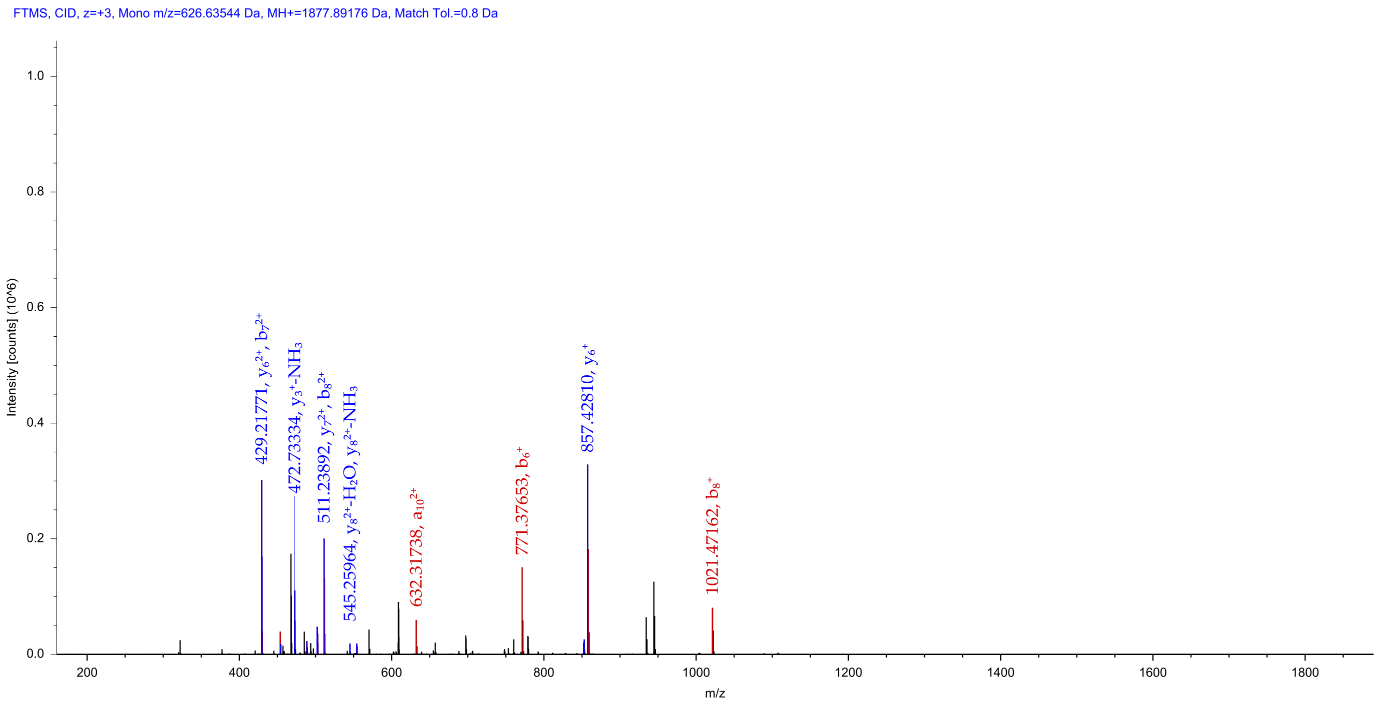

M/Z	Intensity	Matches
771.3765	150586.4	b (6) (1+)
858.4309	182212.4	b (7) (1+)
429.2177	301594.6	b (7) (2+), y (6) (2+)
1021.472	80496.1	b (8) (1+)
502.2342	47546.3	b (8) (2+)-H2O, y (7) (2+)-NH3, b (8) (2+)-NH3, y (7) (2+)-H2O
454.2275	17754.8	y (10) (3+)-H2O, a (11) (3+)
852.8962	25810.6	y (13) (2+)-H2O, y (13) (2+)-NH3, b (13) (2+)
472.7333	110511.8	y (3) (1+)-NH3
857.4281	328406.5	y (6) (1+)
511.2389	200206.8	y (7) (2+), b (8) (2+)
545.2596	18576.9	y (8) (2+)-NH3, y (8) (2+)-H2O

Supplemental Figure 1. Fragmentation spectra of lysine acetylated peptides identified in rice.
