# Supplementary material for: Global Analysis of Lysine Acetylation Suggests the Involvement of Protein Acetylation in Diverse Biological Processes in Rice (Oryza sativa)
Source: PLoS One. 2014 Feb 20;9(2):e89283. doi: 10.1371/journal.pone.0089283 (PMC3930695; doi:10.1371/journal.pone.0089283)
Supplement: Table S4 — Comparison of lysine acetylated sites (Ac sites) and acetylated proteins (Ac proteins) in different organisms. (PDF) [file pone.0089283.s005.pdf]

Organism	Ac Sites	Ac Proteins	Purification	Reference
E. coli	125<br>138<br>1070	84<br>91<br>349	Immunoaffinity<br>Immunoaffinity<br>Immunoaffinity	Yu et al., 2008<br>Zhang et al., 2009<br>Zhang et al., 2013
Salmonella	235	191	Immunoaffinity	Wang et al., 2010
Toxoplasma	411	274	Immunoaffinity	Jeffers & Sullivan 2012
HeLa Cells & Mouse<br>Mouse	388<br>4623	195<br>1800	Immunoaffinity<br>Immunoaffinity	Kim et al., 2006<br>Chen et al., 2012
Human cell line<br>Human liver	3600<br>1300	1750<br>1047	Immunoaffinity<br>Immunoaffinity	Choudhary et al., 2009<br>Zhao et al., 2010
Drosophila	1981	1013	Immunoaffinity	Weinert et al., 2011
Arabidopsis<br><br>Grapevine	91<br>64<br>138	74<br>57<br>-	Immunoaffinity<br>Immunoaffinity<br>Immunoaffinity	Finkemeier et al., 2011<br>Wu et al., 2011<br>Melo-Braga et al., 2012
Rice	66	44	Immunoaffinity	Current Study
